# Continuous‐Flow Amide and Ester Reductions Using Neat Borane Dimethylsulfide Complex

**DOI:** 10.1002/cssc.201903459

**Published:** 2020-02-20

**Authors:** Sándor B. Ötvös, C. Oliver Kappe

**Affiliations:** ^1^ Institute of Chemistry University of Graz, NAWI Graz Heinrichstrasse 28 8010 Graz Austria; ^2^ Center for Continuous Synthesis and Processing (CCFLOW) Research Center Pharmaceutical Engineering (RCPE) Inffeldgasse 13 8010 Graz Austria

**Keywords:** amides, boranes, continuous flow, esters, reduction

## Abstract

Reductions of amides and esters are of critical importance in synthetic chemistry, and there are numerous protocols for executing these transformations employing traditional batch conditions. Notably, strategies based on flow chemistry, especially for amide reductions, are much less explored. Herein, a simple process was developed in which neat borane dimethylsulfide complex (BH_3_⋅DMS) was used to reduce various esters and amides under continuous‐flow conditions. Taking advantage of the solvent‐free nature of the commercially available borane reagent, high substrate concentrations were realized, allowing outstanding productivity and a significant reduction in E‐factors. In addition, with carefully optimized short residence times, the corresponding alcohols and amines were obtained in high selectivity and high yields. The synthetic utility of the inexpensive and easily implemented flow protocol was further corroborated by multigram‐scale syntheses of pharmaceutically relevant products. Owing to its beneficial features, including low solvent and reducing agent consumption, high selectivity, simplicity, and inherent scalability, the present process demonstrates fewer environmental concerns than most typical batch reductions using metal hydrides as reducing agents.

## Graphical abstract

**Go with the flow**: Neat borane dimethylsulfide is used to reduce various esters and amides under continuous‐flow conditions in a simple yet efficient process. The application of the neat reducing agent in combination with highly concentrated substrate solutions at short residence times enables highly productive reductions with fewer environmental concerns than classical metal‐hydride‐mediated batch reductions.

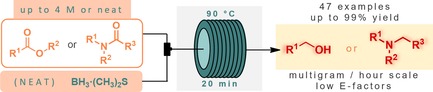

## Introduction

As a consequence of the ubiquity of alcohols and amines, carbonyl reductions are amongst the most crucial transformations in synthetic organic chemistry. In comparison with aldehydes and ketones, reductions of carboxylic acid derivatives are much more challenging, owing to the low electrophilicity of the carbonyl carbon and the intrinsic stability of the involved functional group.^1^ Because of their relevance in organic synthetic practice,^2^ reductions involving amides and esters are particularly important among the various carboxylic acid derivatives.

The most general route for the reduction of amides and esters is the application of hydride reagents such as LiAlH_4_ or NaBH_4_, with the latter typically used at higher temperatures.^3^ Although these reagents are effective and well‐established, major drawbacks include the formation of stoichiometric amounts of metallic waste, the need for laborious workup procedures, and the hazards involved in handling these highly reactive substances.^4^ In the case of amides, catalytic reductive hydrosilylations may represent a useful alternative, even if these reactions are generally much slower than aluminum‐ or boron‐hydride‐mediated reductions.^5^ Alternatively, catalytic hydrogenations may reduce environmental concerns, but they typically require harsh reaction conditions (particularly in the case of amides) and/or costly dedicated catalysts.^6^ From an environmental point of view, heterogeneous catalytic approaches are especially appealing. However, the demanding conditions, along with frequent selectivity issues, strongly limit their synthetic applicability in reductions of carboxylic acid derivatives.3b, 6a

Borane complexes have proven exceedingly useful for carbonyl reductions.3b, 7 They offer similar reactivity to diborane (B_2_H_6_) but have fewer storage and handling concerns than the pyrophoric gas itself. Of the available complexes, BH_3_⋅THF and BH_3_⋅DMS (DMS=dimethylsulfide) are the most popular reagents.^7^ Although BH_3_⋅THF is more reactive than BH_3_⋅DMS, it is commercially available only as 1 m THF solution. In contrast, the more stable BH_3_⋅DMS is available as a neat reagent. Borane complexes are prone to intensive thermal decomposition, and in contact with atmospheric moisture, water, or acids, pyrophoric B_2_H_6_ and H_2_ gases are evolved.7b, 8 Because of these hazards, both BH_3_⋅THF and BH_3_⋅DMS are generally employed in commercially available 1–2 m solutions.^7^ A notable drawback of the application of borane reagents is that they are often required in a significant excess.^7^ This is particularly because of the complex nature of the reduction itself. In the case of amides, for example, numerous hydride equivalents may be consumed through hydrogen evolution and/or multiple rounds of borane complexation. These effects and the amount of the consumed reagent are largely dependent on the electronic and steric properties of the substrate applied.^9^

Today, sustainability is becoming a key target in the fine chemical industry.^10^ However, the transfer from well‐established reactions and reagents to novel chemistries that tend to be more sustainable at the lab‐scale is not always straightforward. Even though the application of metal hydrides and borane reagents has limitations with respect to sustainability and process safety, these substances are routinely employed on manufacturing scales owing to their efficiency and simplicity.^11^

Not only novel chemistries, but also advanced reaction technologies may reduce the environmental impact of chemical processes. Flow chemistry and continuous processing have received an upsurge of interest in the context of green and sustainable syntheses.^12^ Useful properties such as enhanced heat and mass transfer, precise reaction parameter control, inherent scalability, and improved safety^13^ make continuous‐flow techniques especially appealing for the reduction of various functional groups.^14^ For the reduction of aldehydes and ketones, heterogeneous catalytic hydrogenations have been studied extensively under flow conditions.14c, 14d Because of solubility issues involving most hydride reagents, continuous‐flow hydridic reductions often involve solid sources in packed beds, such as supported borohydride species or solid NaBH_4_.^15^ Alternatively, liquid reductants such as LiBHEt_3_, (CH_3_)_4_NBH_4_, or diisobutylaluminum hydride (DIBALH) may be employed.^16^ In a few instances, borane complexes have also been applied in continuous‐flow carbonyl reductions.^17^ In these cases, borane reagents were used as 1–2 m solutions, involving low substrate concentrations and therefore inherently low productivities. Compared with those of aldehydes and ketones, continuous reductions of carboxylic acid derivatives have been less explored.^14^ This can be explained partly by the fact that LiAlH_4_ reductions are virtually incompatible with flow conditions, and that heterogeneous catalytic amide and ester hydrogenations are quite limited owing to the harsh conditions required.3b, 6a Flow reductions of esters to the corresponding aldehydes have been achieved using DIBALH as the reducing agent,^18^ but reports of reductions of amides under flow conditions are extremely scarce in the literature.17b, 19

We recently communicated a multistep flow process for the synthesis of the chiral key intermediate of a well‐known antidepressant and applied neat BH_3_⋅DMS as the reducing agent in the final step of the synthesis.^20^ On the basis of this preliminary result, we hypothesized that the direct application of neat BH_3_⋅DMS in flow mode may act as a general and robust strategy for the reduction of various esters and amides to the corresponding alcohols and amines. By taking advantage of the solvent‐free borane complex in a continuous manner, we anticipated higher productivities than in earlier flow carbonyl reductions and fewer environmental concerns than the most typical metal‐hydride‐mediated batch reductions. Our findings are presented herein.

## Results and Discussion

**Figure 1:**
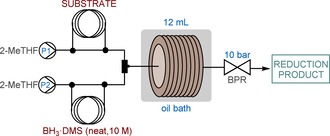
Continuous‐flow reactor setup for the BH_3_⋅DMS‐mediated reductions.

A simple setup was established for the flow reactions using commercially available components, as detailed in the Experimental Section and shown in Figure 1. The substrate and neat BH_3_⋅DMS as reducing agent were pumped as separate streams, and the combined mixture was reacted while passing through a 12 mL PFA coil (1.58 mm inner diameter), which was heated in an oil bath. A backpressure regulator (BPR) was also installed to eliminate solvent boil‐over and minimize gas formation during the reactions.

According to earlier literature findings, reductions of esters with borane reagents require more stringent conditions than those of amides.3b Methyl benzoate was therefore selected as a simple model substrate to investigate the effects of reaction conditions on the continuous‐flow reduction (Table 1). As a greener alternative to traditional solvents,^21^ 2‐methyltetrahydrofuran (2‐MeTHF) was selected as reaction medium and also as carrier solvent.

**Table 1:** Effects of various reaction parameters on the neat BH_3_⋅DMS‐mediated ester reduction under continuous‐flow conditions (see Figure 1).^[a]^

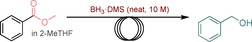							
Entry	*c*_substr._ [m]	*T* [°C]	Flow rate [μL min^−1^]		Substr./BH_3_⋅DMS ratio	*t*_r_ [min]	Conv.^[b]^ [%]
			P1	P2			
1	2	50	250	150	1:3.0	30	42
2	2	90	250	150	1:3.0	30	100
3	2	90	310	90	1:1.5	30	97
4	2	90	330	70	1:1.1	30	85
5	2	90	570	230	1:2.0	15	96
6	2	90	620	180	1:1.5	15	92
7	2	90	450	150	1:1.7	20	97
8	2	90	430	170	1:2.0	20	100
9	4	90	330	270	1:2.0	20	100 (99)^[c]^
10	8 (neat)	90	230	370	1:2.0	20	100 (99)^[c]^

[a] No side product formation, chemoselectivity was 100 % in all reactions. [b] Determined by ^1^H NMR spectroscopy of the crude product. [c] Yield of isolated product in parentheses.

By exploring the temperature dependence of the reaction, it was confirmed that heating is necessary for complete conversion (Table 1, entries 1 and 2). A temperature of 90 °C was selected as an optimum value, keeping in mind that thermal decomposition of the reducing agent may lead to intense gas formation and a decrease in reducing efficacy.7b The flow rate of the substrate and reducing agent stream was fine‐tuned to achieve the lowest possible borane excess at a reasonably short residence time, while maintaining quantitative reduction of the ester moiety (entries 3–8). A residence time of 20 min in combination with a substrate/BH_3_⋅DMS ratio of 1:2.0 was found optimal, and these conditions were subsequently employed to investigate the reaction at higher concentrations in an attempt to maximize synthetic productivity. We were pleased to find that the reaction was smooth and stable using a substrate concentration of 4 m and even employing neat methyl benzoate (8 m concentration). In both cases, the product stream was collected for 10 min, resulting in 1.41 g of pure benzyl alcohol at 4 m, and 1.97 g in the case of the neat substrate after extractive workup (entries 9 and 10). These gave isolated yields of 99 % (conversion and chemoselectivity was 100 % in both cases) and corresponded to productivities of 8.46 and 11.82 g h^−1^ of pure product, respectively. The E‐factor was calculated for both optimized reactions (entries 9 and 10), and also for a hypothetical reaction employing methyl benzoate at a concentration of 1 m and two equivalents of BH_3_⋅DMS as 1 m 2‐MeTHF solution instead of the neat reagent. For the reaction involving 4 m substrate concentration, a remarkable E‐factor of 2.7 was obtained, and in the case of neat methyl benzoate, it decreased further to 1.7. In contrast, for the hypothetical reaction under more dilute conditions (assuming quantitative conversion and complete selectivity toward the corresponding alcohol) a much higher E‐factor of 22.8 was calculated, which clearly indicates the importance of solvent‐free or highly concentrated conditions in sustainable process development.

After becoming familiar with the conditions of the flow ester reduction, we next investigated the scope and generality of the methodology (Table 2). All reactions were performed with a residence time of 20 min at 90 °C. 2‐MeTHF was used as solvent in all cases, at 4 m concentration when possible. In the case of lower substrate solubility, the concentration was reduced accordingly, and individual flow rates were adjusted to give the desired residence time and reducing agent excess.

**Table 2:** Substrate scope of the neat BH_3_⋅DMS‐mediated ester reduction under continuous‐flow conditions (see Figure 1 for the flow setup).^[a]^

Entry	Substrate	Product	*c*_substr._ [m]	Conv.^[b]^ [%]	Sel.^[b]^ [%]
1^[c]^	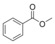	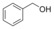	4	100 (99)^[d]^	100
2^[c]^	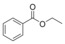	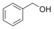	4	100	100
3^[c]^	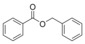	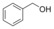	4	98	100
4^[e]^	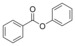	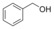	2	93	100
5^[c]^	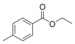	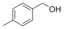	4	100 (99)^[d]^	100
6^[c]^	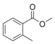	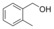	4	100	100
7^[c]^	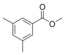	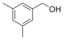	4	100	100
8^[c]^	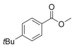	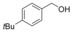	4	100	100
9^[c]^	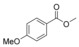	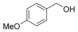	4	99	100
10^[c]^	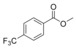	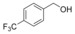	4	98	100
11^[c]^	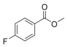	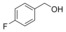	4	100 (98)^[d]^	100
12^[c]^	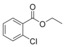	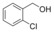	4	100	100
13^[c]^	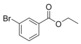	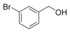	4	95	100
14^[c]^	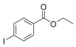	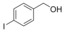	4	96	100
15^[c]^	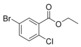	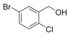	4	99	100
16^[f]^	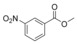	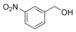	1	67	99^[g]^
17^[h]^	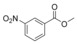	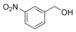	1	94	98^[g]^
18^[i]^	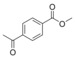	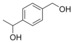	0.6	100	89^[j]^
19^[k]^	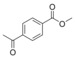	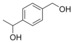	0.6	100	100
20^[e]^	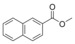	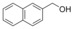	2	100 (97)^[d]^	100
21^[f]^	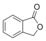	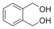	1	100	100
22^[c]^	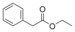	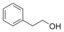	4	100	100
23^[e]^	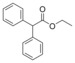	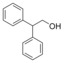	2	100	100
24^[e]^	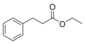	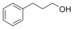	2	100 (99)^[d]^	100
25^[c]^	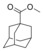	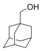	4	100	100
26^[c]^	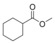	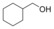	4	100 (95)^[d]^	100
27^[c]^	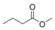	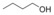	4	100	100
28^[c]^	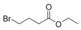	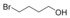	4	100	100

[a] Reaction conditions: substrate in 2‐MeTHF, *T=*90 °C, *t*_r_=20 min. [b] Determined by ^1^H NMR spectroscopy of the crude product. [c] P1 at 330 μL min^−1^, P2 at 270 μL min^−1^, substrate/BH_3_⋅DMS ratio 1:2.0. [d] Yield of isolated product in parentheses. [e] P1 at 430 μL min^−1^, P2 at 170 μL min^−1^, substrate/BH_3_⋅DMS ratio 1:2.0. [f] P1 at 500 μL min^−1^, P2 at 100 μL min^−1^, substrate/BH_3_⋅DMS ratio 1:2.0. [g] Minor product: 3‐aminobenzyl alcohol. [h] P1 at 430 μL min^−1^, P2 at 170 μL min^−1^, substrate/BH_3_⋅DMS ratio 1:4.0. [i] P1 at 510 μL min^−1^, P2 at 90 μL min^−1^, substrate/BH_3_⋅DMS ratio 1:2.9. [j] Minor product: methyl 4‐(1‐hydroxyethyl)benzoate. [k] P1 at 490 μL min^−1^, P2 at 110 μL min^−1^, substrate/BH_3_⋅DMS ratio 1:3.7.

Not only methyl benzoate but also ethyl, benzyl, and even phenyl benzoate gave high conversions (93–100 %) and complete chemoselectivity (Table 2, entries 1–4). In the case of phenyl benzoate, the slight decrease in conversion may be explained by the extensive conjugation involving both aromatic rings. Methyl and ethyl benzoates exhibiting diverse substitution patterns were also subjected to the optimized reaction conditions (entries 5–20). We were delighted to find excellent reactivities in the cases of various electron‐donating and electron‐withdrawing substituents, irrespective of their position on the aromatic ring. Even *ortho*‐ and multisubstituted compounds were transformed smoothly to the corresponding alcohols and gave conversions of ≥99 % and complete chemoselectivities (entries 6, 7, 12, and 15). Notably, substrates that contained halogen‐substituted benzene rings were reduced with these functional groups remaining intact (entries 11–15). Although methyl 3‐nitrobenzoate showed somewhat lower reactivity, a satisfactory conversion of 94 % was achieved by doubling the reducing agent excess (entries 16 and 17). The nitro group was not reduced under these conditions, and only traces of 3‐aminobenzyl alcohol were detected as a side product. In the reaction of methyl 4‐acetylbenzoate, both the acetyl and ester moieties were reduced, and the corresponding diol was obtained selectively upon using a substrate/BH_3_⋅DMS ratio of 1:3.7 (entries 18 and 19). Phthalide, an aromatic γ‐lactone, was converted quantitatively and selectively to the corresponding diol (entry 21). Benzylic esters (either branched or nonbranched) and ethyl hydrocinnamate underwent flow reduction with perfect results (entries 22–24). Of the aliphatic esters investigated, not only linear ones, but also bulkier adamantane‐ and cyclohexanecarboxylates were well tolerated, providing the corresponding alcohols in quantitative and selective reactions (entries 25–28). In representative examples, the alcohol products were isolated to give yields of 95–99 % (Table 2, shown in parentheses).

The flow conditions used for the esters were next applied to neat BH_3_⋅DMS‐mediated reductions of amides (Table 3). Owing to their significantly lower solubility, the highest concentration employed for the amide substrates was 1 m, and in some cases, 2‐MeTHF was replaced by THF to provide clean solutions. Excellent results were obtained in reductions of acetanilide and its substituted derivatives (entries 1–12). Functional groups such as methyl, methoxy, trifluoromethyl, amino, and halogen groups were well tolerated on the aromatic rings, and reactions proceeded smoothly with conversions of ≥96 % and 100 % chemoselectivity in all instances (isolated yields were 96–98 % in representative examples). Upon reaction of methyl 2‐acetamidobenzoate, a reducing agent amount of four equivalents was sufficient to reduce both ester and amide moieties quantitatively and selectively, and to give the corresponding amino alcohol in an isolated yield of 95 % (entry 13). From a series of aromatic amides, formanilide, benzanilide, and *N*‐benzylbenzamide were also tested, besides acetanilides (entries 14–16). Formanilide gave quantitative conversion and complete chemoselectivity (entry 14). However, in the case of benzanilide, conversion was somewhat lower owing to the extensive conjugation of the amide bond (entry 15), similar to phenyl benzoate in the ester reductions. In the case of *N*‐benzylbenzamide, conversion was 98 %, but apart from dibenzylamine, benzylamine was formed as side product to an extent of 15 % as a result of undesired C−N bond cleavage (entry 16). Tertiary amides such as *N*‐methyl‐*N*‐phenylacetamide, *N*,*N*‐diphenylacetamide, and *N*‐acetylindoline were reduced quantitatively to the corresponding amines (entries 17–19). Aliphatic amides performed comparably well to the aromatic ones, with conversions of 98 and 84 % in the cases of 1‐acetamidoadamantane and laurolactam, respectively (entries 20 and 21). Despite the applied backpressure, in most amide reductions (entries 1–16), some visible gas formation (presumably H_2_) occurred in the reaction coil, which did not influence the system stability. In a few instances, some precipitation also occurred in the heated reactor zone (e.g., entries 14, 17–19) which could be handled by the BPR applied (see the Experimental Section).

**Table 3:** Reduction of various amides with neat BH_3_⋅DMS under continuous‐flow conditions (see Figure 1 for the flow setup).^[a]^

Entry	Substrate	Product	*c*_substr._^[b]^ [m]	Conv.^[c]^ [%]	Sel.^[c]^ [%]
1^[d]^	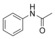	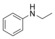	1 (2‐MeTHF)	100 (98)^[e]^	100
2^[f]^	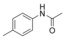	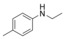	0.6 (THF)	96	100
3^[f]^	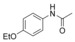	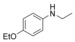	0.6 (THF)	98	100
4^[d]^	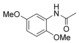	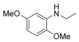	1 (2‐MeTHF)	100	100
5^[d]^	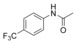	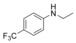	1 (THF)	100	100
6^[f]^	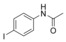	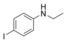	0.6 (THF)	100	100
7^[f]^	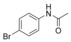	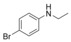	0.6 (THF)	100 (96)^[e]^	100
8^[d]^	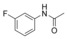	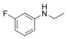	1 (2‐MeTHF)	100	100
9^[d]^	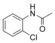	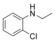	1 (2‐MeTHF)	100 (96)^[e]^	100
10^[f]^	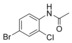	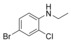	0.6 (THF)	100	100
11^[g]^	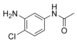	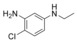	0.3 (2‐MeTHF)	100	100
12^[g]^	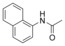	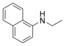	0.3 (THF)	100	100
13^[h]^	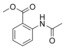	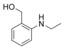	1 (2‐MeTHF)	100 (95)^[e]^	100
14^[d]^	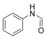	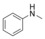	1 (2‐MeTHF)	100	100
15^[f]^	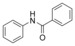	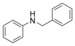	0.6 (THF)	88 (81)^[e]^	100
16^[d]^	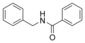	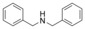	1 (2‐MeTHF)	98	85^[i]^
17^[d]^	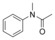	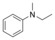	1 (2‐MeTHF)	100	100
18^[f]^	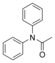	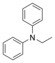	0.6 (2‐MeTHF)	100	100
19^[f]^	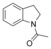	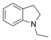	0.6 (THF)	100	100
20^[f]^	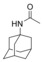	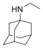	0.6 (2‐MeTHF)	98	100
21^[f]^	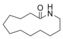	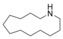	0.6 (THF)	84	100

[a] Reaction conditions: *T=*90 °C, *t*_r_=20 min. [b] Solvent used is shown in parentheses. [c] Determined by ^1^H NMR spectroscopy of the crude product. [d] P1 at 500 μL min^−1^, P2 at 100 μL min^−1^, substrate/BH_3_⋅DMS ratio 1:2.0. [e] Yield of isolated product. [f] P1 at 530 μL min^−1^, P2 at 70 μL min^−1^, substrate/BH_3_⋅DMS ratio 1:2.2. [g] P1 at 560 μL min^−1^, P2 at 40 μL min^−1^, substrate/BH_3_⋅DMS ratio 1:2.4. [h] P1 at 430 μL min^−1^, P2 at 170 μL min^−1^, substrate/BH_3_⋅DMS ratio 1:4.0. [i] Minor product: benzylamine.

**Figure 2:**
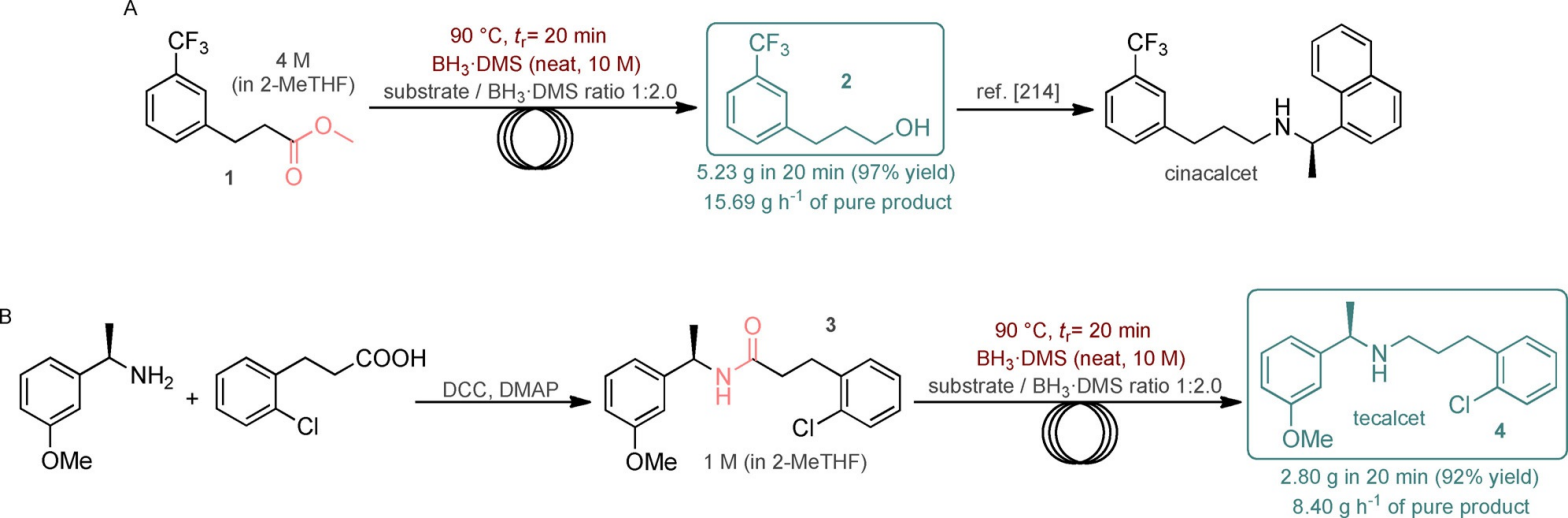
Continuous‐flow synthesis of pharmaceutically relevant products. (Substrate feed: 330 μL min^−1^ for A and 500 μL min^−1^ for B. Reducing agent feed: 270 μL min^−1^ for A and 100 μL min^−1^ for B. See also Figure 1).

Finally, to demonstrate the practical utility of this novel method, we attempted the multigram‐scale synthesis of pharmaceutically relevant products by applying neat BH_3_⋅DMS‐mediated flow reductions. Cinacalcet is a first‐in‐class drug acting as calcimimetic. It is sold under the brand names Sensipar and Mimpara and used for the treatment of hyperparathyroidism and parathyroid carcinoma.^22^ There are numerous procedures reported for its synthesis, such as approaches involving reductive amination in which alcohol **2** acts as an important intermediate.^23^ To synthesize **2** in a simple and scalable manner, we attempted the flow reduction of the corresponding ester (Figure 2 A). Ester **1**, employed as a 4 m solution in 2‐MeTHF, was reduced quantitatively and selectively to alcohol **2** under standard flow conditions. The product stream was collected for 20 min to obtain 5.23 g of pure **2** (97 % yield) after workup, which ensured a productivity of 15.69 g h^−1^. Notably, the process involved a low amount of waste formation, as indicated by an E‐factor of only 1.1. Alcohol **2** can be transformed readily to the actual drug according to literature procedures,^23^ such as a one‐pot oxidation–reductive amination cascade as reported recently by Cossy and co‐workers.^24^

The compound named R‐568 or tecalcet was originally investigated for its similar biological effects to those of cinacalcet.^25^ More recently, it has shown potential in related areas, for example as a vasculotrope agent.^26^ We attempted its synthesis by means of *N*,*N′*‐dicyclohexylcarbodiimide (DCC)‐mediated amidation and subsequent amide reduction under standard flow conditions, employing amide **3** in a 1 m 2‐MeTHF solution (Figure 2 B). Gratifyingly, amide **3** was reduced quantitatively and selectively to the corresponding amine, and after workup, 2.80 g of pure tecalcet was obtained, corresponding to 92 % isolated yield. The productivity of the process was 8.40 g h^−1^ of pure product, and the E‐factor was 2.9.

## Conclusion

A continuous‐flow protocol utilizing neat BH_3_⋅DMS (DMS=dimethylsulfide) was developed for amide and ester reductions. The process did not require any costly special flow equipment but only a simple heated coil reactor from commercially available components. The effects of the reaction temperature and the individual flow rates were optimized carefully to minimize reducing agent excess while maintaining a reasonably short residence time. Ester reductions were completed in 2‐MeTHF as solvent, at concentrations typically as high as 4 m. In the case of methyl benzoate, the flow reduction was successful even without any solvent present, that is, by pumping the neat substrate and reducing agent streams. Owing to their lower solubility, amide reductions were performed at concentrations as high as 1 m, using 2‐MeTHF or THF as solvent. The scope and generality of the flow protocol was demonstrated through reductions of a diverse set of aromatic and aliphatic amide and ester model substrates (47 examples). In most examples, excellent conversions and selective reductions were observed, and the corresponding amine or alcohol products were isolated in pure form, typically without chromatographic purification. The preparative utility of the flow process was verified by the multigram‐scale synthesis of the calcimimetic tecalcet and also by that of a key intermediate of the blockbuster drug cinacalcet. The application of the neat borane complex together with the highly concentrated substrate solutions and short residence times enabled significant chemical intensification and ensured productivities on the multigram‐per‐hour scale. Importantly, owing to the low amounts of solvent utilized, remarkably low E‐factors were reached, and the risks associated with the handling of the potentially dangerous borane reagent were reduced by the inherently safer flow process. As a result of its simplicity, low solvent and reducing agent consumption, high selectivity, and scalability, the present protocol has improved sustainability compared with standard batch procedures. We therefore believe that our flow method may find applications in sustainable industrial production.

## Experimental Section

### General information

All solvents and chemicals were obtained from commercial vendors (Sigma–Aldrich, TCI, Alfa Aesar, or VWR) and were used as received, without further purification. Chromatographic purification was performed by using a Biotage Isolera automated flash chromatography system with cartridges packed with KP‐SIL (60 Å, 32–63 μm particle size). Analytical thin‐layer chromatography (TLC) was performed on Merck silica gel 60 GF254 plates. Compounds were visualized by means of UV or KMnO_4_. ^1^H and ^13^C NMR spectra were recorded on a Bruker Avance III 300 MHz instrument at ambient temperature, in CDCl_3_ as solvent, at 300 and 75 MHz, respectively. Chemical shifts (*δ*) are reported in ppm using trimethylsilane (TMS) as internal standard. Coupling constants are given in Hz units. Liquid chromatography (LC)–MS analyses were performed on a Shimadzu HPLC system (DGU20A degasser, SIL‐20A autosampler, CTO20A column oven, LC‐20AD pumps) using a Macherey–Nagel Nucleodur C18 HTec column (150×4.6 mm, particle size 5 μm) at 37 °C with mobile phases A [H_2_O/acetonitrile, 9:1 *v*/*v* + 0.1 % trifluoroacetic acid (TFA)] and B (acetonitrile + 0.1 % TFA) at a flow rate of 0.6 mL min^−1^. Compounds were detected with a diode array detector (SPDM20A) prior to electrospray ionization (ESI) using a Shimadzu LCMS‐QP2020 instrument. The ESI‐MS was operated in positive mode with a scan range of 50–450 *m*/*z*. GC–MS spectra were recorded using a Shimadzu GCMS‐QP2010 SE mass spectrometer (EI, 70 V). An Rtx‐5MS column (30 m×0.25 mm×0.25 μm) was used, with helium as carrier gas (40 cm s^−1^ linear velocity). The injector temperature was set to 280 °C. After 1 min at 50 °C, the temperature was increased by 25 °C min^−1^ to 300 °C and kept at 300 °C for 3 min. Optical rotation was measured in CHCl_3_ (HPLC‐grade) at 20 °C against the sodium d‐line (*λ*=589 nm) on a PerkinElmer Polarimeter 341 using a cell of 10 cm pathlength. The E‐factor was calculated by dividing the mass of waste generated by the mass of product formed, not including the reaction workup. The mass of the waste did not include water.

### General procedure for flow reactions

A solution of the appropriate ester or amide substrate was prepared in dry 2‐MeTHF or dry THF (under Ar atmosphere). Commercially available solvent‐free BH_3_⋅DMS (10 m) was used as reducing agent. Starting materials were pumped as separate feeds by using a UNIQSIS Binary Pump Module equipped with two high‐pressure HPLC pumps (P1: substrate solution; P2: reducing agent), two injection valves with sample loops [perfluoroalkoxy alkane (PFA) tubing, 1/16“ outer diameter, 0.8 mm inner diameter] and a pressure sensor to monitor the system pressure. Dry 2‐MeTHF was used as carrier solvent. The liquid streams were combined in a Y‐mixer at room temperature, and the resulting solution was directed through a 12 mL reaction coil (PFA tubing, 1/8” outer diameter, 1.58 mm inner diameter), which was heated in an oil bath. The flow system was pressurized by applying an adjustable BPR assembly from Vapourtec (part no.: 50‐1315) at 10 bar (see Figure 1 and the Supporting Information for a photograph of the flow setup.) After reaching steady state, the product stream was collected for 5 or 10 min in smaller‐scale model reactions, and for 20 min in the syntheses of **2** and **4**. For safe decomposition of the unreacted reducing agent, the stream exiting the reactor was collected in a flask containing a well‐stirred mixture of 3 m HCl and 2‐MeTHF (1:1). After the collection period, the mixture was treated with NaOH solution (2 m) until it reached pH 10. The resultant mixture was extracted three times with EtOAc. The combined organic layers were washed with brine, dried over Na_2_SO_4_, and concentrated under reduced pressure. In most of the reactions, pure products were achieved after extractive workup and evaporation. If conversion and/or chemoselectivity was <98 %, chromatographic purification was performed using mixtures of ethyl acetate/40–60 petroleum ether as eluent. The reaction products were characterized by means of NMR and MS techniques. For the synthesis of **4**, amide **3** was prepared from the corresponding amine and carboxylic acid in the presence of DCC and 4‐dimethylaminopyridine (DMAP) according to the literature procedure.^27^

**Caution**: BH_3_⋅DMS decomposes thermally or in the presence of atmospheric moisture, water, and acids, resulting in flammable gases (B_2_H_6_ and H_2_) and boric acid. Extreme care must therefore be taken when handling. Dry conditions must be ensured during experimentation and all equipment must be set up in a well‐ventilated fume hood. A thorough safety assessment should be made before conducting any experiments.

## Conflict of interest

*The authors declare no conflict of interest*.

## Supporting information

As a service to our authors and readers, this journal provides supporting information supplied by the authors. Such materials are peer reviewed and may be re‐organized for online delivery, but are not copy‐edited or typeset. Technical support issues arising from supporting information (other than missing files) should be addressed to the authors.

Supplementary
