# Supplementary material for: Continuous‐Flow Amide and Ester Reductions Using Neat Borane Dimethylsulfide Complex
Source: ChemSusChem. 2020 Feb 20;13(7):1800–7. doi: 10.1002/cssc.201903459 (PMC7187139; doi:10.1002/cssc.201903459)
Supplement: Supplementary file 1 — Supplementary [file CSSC-13-1800-s001.pdf]

CHEMISTRY & SUSTAINABILITY
CHEMSUSCHEM
ENERGY & MATERIALS

Supporting Information

Continuous-Flow Amide and Ester Reductions Using Neat Borane Dimethylsulfide Complex

Sándor B. Ötvös*[a] and C. Oliver Kappe*[a, b]

cssc_201903459_sm_miscellaneous_information.pdf

Table of Contents

1. Picture of the continuous flow set-up S2

2. Analytical data S3

3. Collection of NMR Spectra S12

4. References S64

1. Picture of the continuous flow set-up

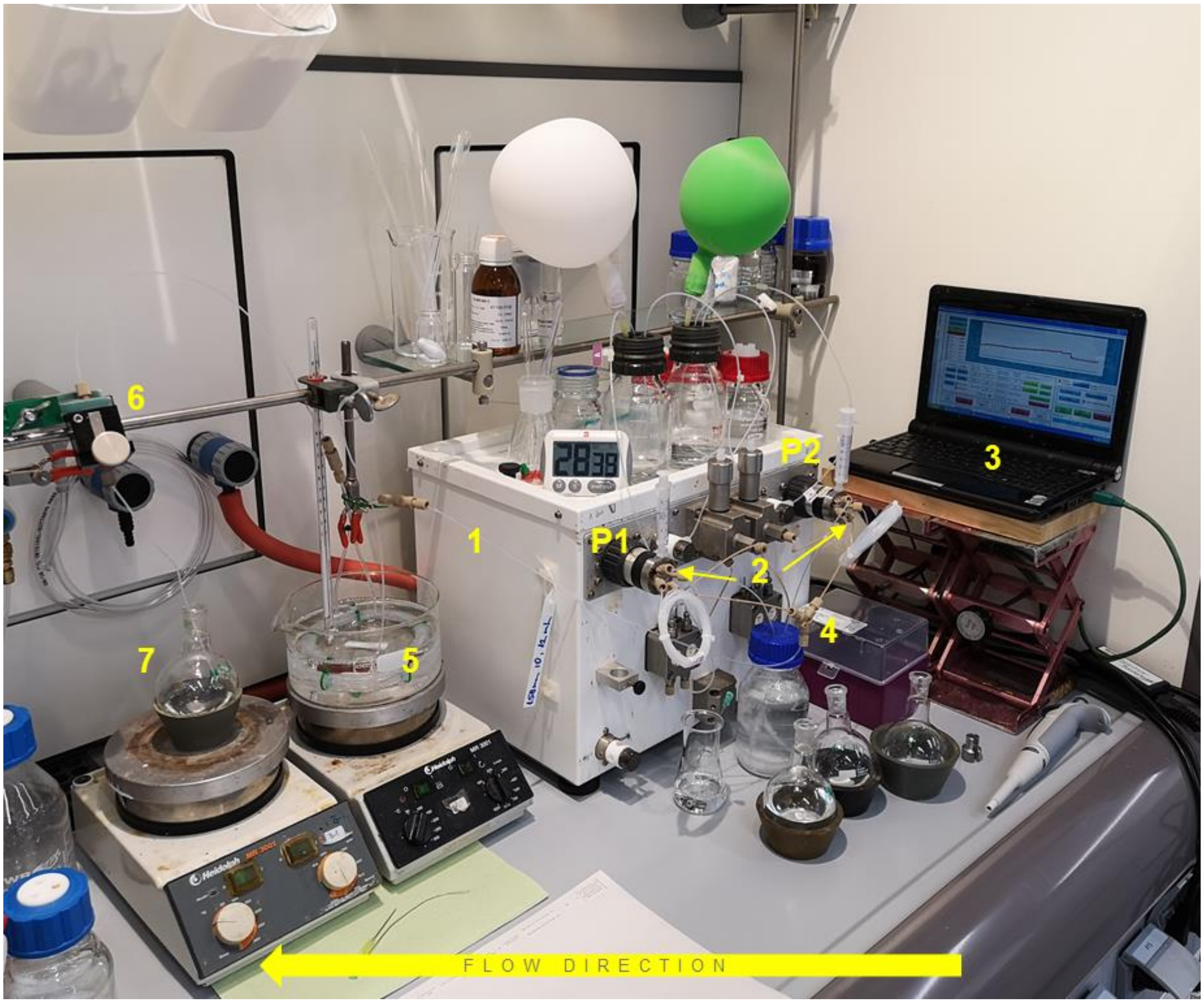

1: UNIQSIS Binary Pump Module

2: 6-port injection valves with sample loops

3: control software for the pump module

4: Y-shaped mixer

5: 12 mL reaction coil in heated oil bath

6: Vapourtec adjustable BPR

7: product collection

2. Analytical data

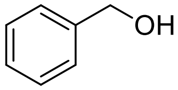

benzyl alcohol
NMR data is in agreement with the literature reference.[1]
MS (EI): m/z = 108.10 (M^+), 91.10, 79.10, 65.05, 51.05

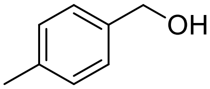

p-tolylmethanol
NMR data is in agreement with the literature reference.[1]
MS (EI): m/z = 122.15 (M^+), 107.10, 91.10, 79.10, 65.05, 51.05

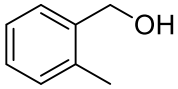

o-tolylmethanol
NMR data is in agreement with the literature reference.[2]
MS (EI): m/z = 122.15 (M^+), 107.05, 104.10, 91.10, 79.10, 65.05, 51.05

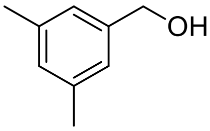

(3,5-dimethylphenyl)methanol
NMR data is in agreement with the literature reference.[3]
MS (EI): m/z = 136.15 (M^+), 121.10, 118.15, 107.10, 91.10, 77.05, 65.05, 51.05

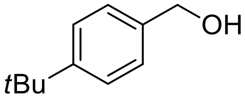

(4-(*tert*-butyl)phenyl)methanol
NMR data is in agreement with the literature reference.[1]
MS (EI): m/z = 164.20 (M^+), 149.15, 131.20, 121.15, 105.10, 91.10, 79.10, 65.00, 51.05

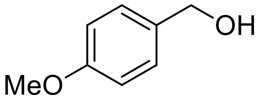

(4-methoxyphenyl)methanol

NMR data is in agreement with the literature reference.[1]
MS (EI): m/z = 138.15 (M^+), 121.10, 107.10, 91.05, 77.05, 65.05, 51.00

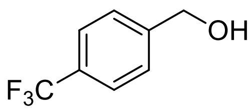
(4-(trifluoromethyl)phenyl)methanol
NMR data is in agreement with the literature reference.[2]
MS (EI): m/z = 176.10 (M^+), 157.10, 145.05, 127.10, 107.10, 95.05, 89.05, 79.10, 69.00, 63.05, 61.05

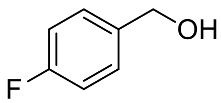
(4-fluorophenyl)methanol
NMR data is in agreement with the literature reference.[1]
MS (EI): m/z = 126.10 (M^+), 105.10, 97.10, 83.05, 77.05, 69.05, 63.10, 57.05, 51.05

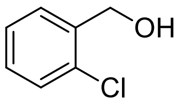
(2-chlorophenyl)methanol
NMR data is in agreement with the literature reference.[4]
MS (EI): m/z = 142.10 (M^+), 125.05, 113.10, 107.10, 89.05, 77.05, 75.00, 63.05, 51.05

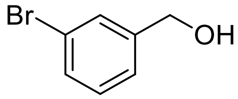
(3-bromophenyl)methanol
NMR data is in agreement with the literature reference.[5]
MS (EI): m/z = 187.00 (M^+), 157.00, 107.10, 79.10, 74.05, 63.05, 51.05

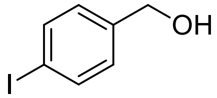
(4-iodophenyl)methanol
NMR data is in agreement with the literature reference.[6]
MS (EI): m/z = 134.00 (M^+), 126.95, 107.10, 89.10, 79.10, 74.05, 63.05, 51.05

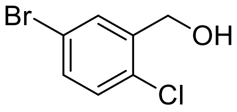

(5-bromo-2-chlorophenyl)methanol
NMR data is in agreement with the literature reference.[7]
MS (EI): m/z = 222.00 (M^+), 185.10, 157.05, 141.05, 123.10, 112.05, 89.05, 77.05, 63.10, 51.05

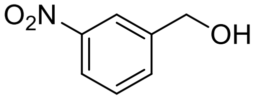

(3-nitrophenyl)methanol
NMR data is in agreement with the literature reference.[8]
MS (EI): m/z = 153.15 (M^+), 136.10, 121.10, 107.10, 89.05, 77.10, 63.10, 51.10

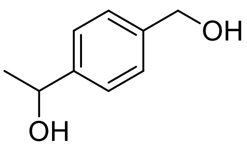

1-(4-(hydroxymethyl)phenyl)ethanol
NMR data is in agreement with the literature reference.[9]
MS (EI): m/z = 152.10 (M^+), 137.15, 134.00, 121.00, 107.05, 91.10, 79.10, 65.10, 51.05

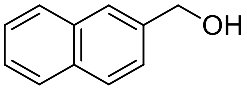

naphthalen-2-ylmethanol
NMR data is in agreement with the literature reference.[2]
MS (EI): m/z = 158.10 (M^+), 141.15, 129.15, 115.10, 102.15, 87.10, 77.10, 63.05, 51.00

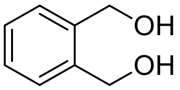

1,2-phenylenedimethanol
NMR data is in agreement with the literature reference.[4]
MS (EI): m/z = 120.10, 105.10, 91.10, 77.05, 65.10, 51.05

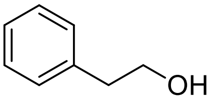

2-phenylethanol
NMR data is in agreement with the literature reference.[10]
MS (EI): m/z = 122.15 (M^+), 91.10, 77.05, 65.10, 51.05

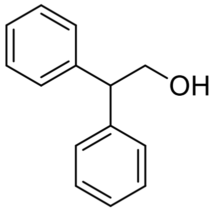

2,2-diphenylethanol
NMR data is in agreement with the literature reference.[10]
MS (EI): m/z = 198.15 (M^+), 167.15, 152.10, 139.05, 128.05, 115.10, 91.10, 77.05, 63.00, 51.05

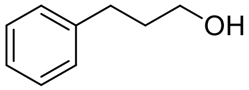

3-phenylpropan-1-ol
NMR data is in agreement with the literature reference.[1]
MS (EI): m/z = 136.15 (M^+), 117.15, 103.10, 91.10, 77.05, 65.05, 51.05

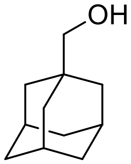

(3*r*,5*r*,7*r*)-adamantan-1-ylmethanol
NMR data is in agreement with the literature reference.[2]
MS (EI): m/z = 166.30 (M^+), 135.20, 107.15, 93.10, 79.10, 67.10, 55.10

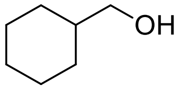

cyclohexylmethanol
NMR data is in agreement with the literature reference.[1]
MS (EI): m/z = 96.15, 83.10, 67.10, 55.10, 51.05

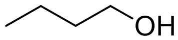

butan-1-ol
NMR data is in agreement with the literature reference.[11]

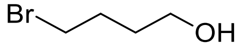

4-bromobutan-1-ol
NMR data is in agreement with the literature reference.[12]
MS (EI): m/z = 134.05, 106.00, 92.95, 71.10, 55.10

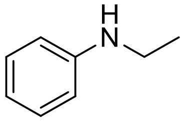

N-ethylaniline
NMR data is in agreement with the literature reference.[13]
MS (ESI): m/z = 163.10 $(M+MeCN+H)^+$, 122.15 $(M+H)^+$

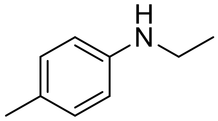

N-ethyl-4-methylaniline
NMR data is in agreement with the literature reference.[13]
MS (ESI): m/z = 177.10 $(M+MeCN+H)^+$, 136.10 $(M+H)^+$

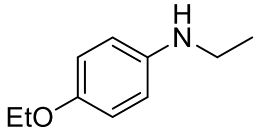

4-ethoxy-*N*-ethylaniline
NMR data is in agreement with the literature reference.[14]
MS (ESI): m/z = 207.10 $(M+MeCN+H)^+$, 166.10 $(M+H)^+$

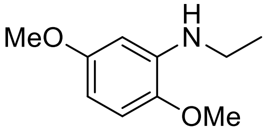

N-ethyl-2,5-dimethoxyaniline
^{1}H-NMR (300 MHz, $CDCl_3$) δ 6.70 (d, *J*= 8.61 Hz, 1H; Ar-H), 6.28 (d, *J*= 2.99 Hz, 1H; Ar-H), 6.22–6.16 (m, 1H; Ar-H), 4.15 (s, 1H; NH), 3.84 (s, 3H; OCH_3), 3.80 (s, 3H, OCH_3), 3.19 (q, *J*= 7.30 Hz, 2H; CH_2), 1.33 (t, *J*= 7.17 Hz, 3H; CH_3); ^{13}C-NMR (75 MHz, $CDCl_3$) δ 154.9, 141.5, 139.5, 109.8, 98.4, 98.0, 55.9, 55.5, 38.1, 14.8.
MS (ESI): m/z = 223.10 $(M+MeCN+H)^+$, 182.10 $(M+H)^+$

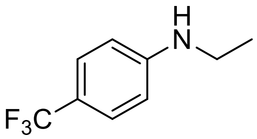

N-ethyl-4-(trifluoromethyl)aniline
NMR data is in agreement with the literature reference.[13]
MS (ESI): m/z = 272.05 $(M+2MeCN+H)^+$, 231.05 $(M+MeCN+H)^+$, 190.10 $(M+H)^+$

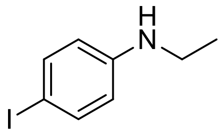

N-ethyl-4-iodoaniline
NMR data is in agreement with the literature reference.[15]
MS (ESI): m/z = 289.00 $(M+MeCN+H)^+$, 247.95 $(M+H)^+$

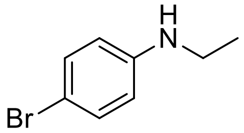

4-bromo-*N*-ethylaniline
NMR data is in agreement with the literature reference.[13]
MS (ESI): m/z = 283.00 $(M+2MeCN+H)^+$, 241.95 $(M+MeCN+H)^+$, 201.00 $(M+H)^+$

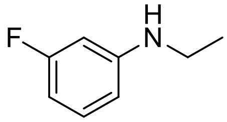

3-fluoro-*N*-ethylaniline
NMR data is in agreement with the literature reference.[13]
MS (ESI): m/z = 222.10 $(M+2MeCN+H)^+$, 181.10 $(M+MeCN+H)^+$, 140.10 $(M+H)^+$

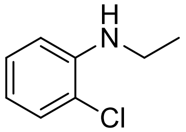

2-chloro-*N*-ethylaniline
NMR data is in agreement with the literature reference.[16]
MS (ESI): m/z = 197.05 $(M+MeCN+H)^+$, 156.05 $(M+H)^+$

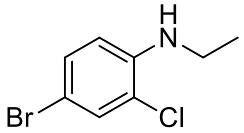

4-bromo-2-chloro-*N*-ethylaniline
^{1}H-NMR (300 MHz, $CDCl_3$) δ 7.39 (d, *J*= 2.16 Hz, 1H; Ar-H), 7.28–7.20 (m, 1H; Ar-H), 6.53 (d, *J*= 8.68 Hz, 1H; Ar-H), 4.20 (s, 1H; NH), 3.19 (q, *J*= 7.29 Hz, 2H; CH_2), 1.32 (t, *J*= 7.20 Hz, 3H; CH_3); ^{13}C-NMR (75 MHz, $CDCl_3$) δ 143.3, 131.2, 130.6, 119.5, 112.1, 107.3, 38.3, 14.6.
MS (ESI): m/z = 276.95 $(M+MeCN+H)^+$, 235.95 $(M+H)^+$

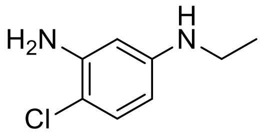

4-chloro-N^1-ethylbenzene-1,3-diamine
^{1}H-NMR (300 MHz, $CDCl_3$) δ 7.02 (d, *J*= 8.15 Hz, 1H; Ar-H), 6.06–5.99 (m, 2H; Ar-H), 3.72 (s, 3H; NH), 3.11 (q, *J*= 7.29 Hz, 2H; CH_2), 1.25 (t, *J*= 7.16 Hz, 3H; CH_3); ^{13}C-NMR (75 MHz, $CDCl_3$) δ 148.1, 143.4, 129.7, 108.2, 105.0, 99.7, 38.7, 14.8.
MS (ESI): m/z = 212.05 $(M+MeCN+H)^+$, 171.05 $(M+H)^+$

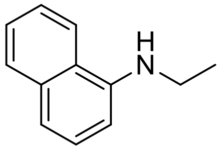

N-ethylnaphthalen-1-amine
NMR data is in agreement with the literature reference.[13]
MS (ESI): m/z = 213.10 $(M+MeCN+H)^+$, 172.10 $(M+H)^+$

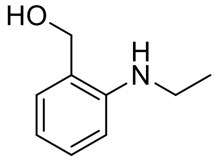

(2-(ethylamino)phenyl)methanol
NMR data is in agreement with the literature reference.[17]
MS (ESI): m/z = 152.10 $(M+H)^+$

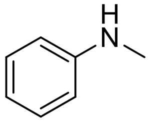

N-methylaniline
NMR data is in agreement with the literature reference.[13]
MS (ESI): m/z = 149.10 $(M+MeCN+H)^+$, 108.15 $(M+H)^+$

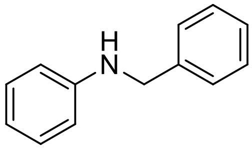

N-benzylaniline
NMR data is in agreement with the literature reference.[13]
MS (ESI): m/z = 225.10 $(M+MeCN+H)^+$, 184.10 $(M+H)^+$

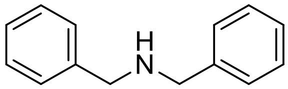

dibenzylamine

NMR data is in agreement with the literature reference.[16]

MS (ESI): m/z = 239.10 $(M+MeCN+H)^+$, 198.10 $(M+H)^+$

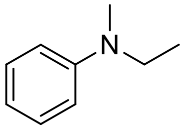

N-ethyl-*N*-methylaniline

NMR data is in agreement with the literature reference.[18]

MS (ESI): m/z = 177.10 $(M+MeCN+H)^+$, 136.10 $(M+H)^+$

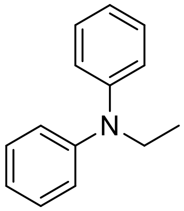

N-ethyl-*N*-phenylaniline

NMR data is in agreement with the literature reference.[19]

MS (ESI): m/z = 239.10 $(M+MeCN+H)^+$, 198.10 $(M+H)^+$

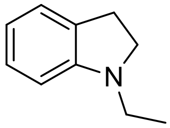

1-ethylindoline

NMR data is in agreement with the literature reference.[18]

MS (ESI): m/z = 189.10 $(M+MeCN+H)^+$, 148.10 $(M+H)^+$

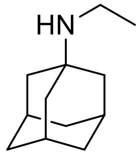

(3*s*,5*s*,7*s*)-*N*-ethyladamantan-1-amine

^{1}H-NMR (300 MHz, $CDCl_3$) δ 2.60 (q, *J*= 7.10 Hz, 2H; CH_2), 2.08–1.99 (m, 3H), 1.70–1.47 (m, 13H), 1.06 (t, *J*= 7.15 Hz, 3H; CH_3); ^{13}C-NMR (75 MHz, $CDCl_3$) δ 50.4, 42.7, 36.8, 34.5, 29.6, 16.2.

MS (ESI): m/z = 221.5 $(M+MeCN+H)^+$, 180.15 $(M+H)^+$

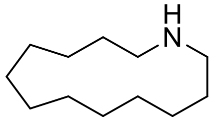

azacyclotridecane
NMR data is in agreement with the literature reference.[20]
MS (ESI): m/z = 225.20 (M+MeCN+H)$^+$, 184.20 (M+H)$^+$

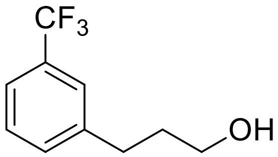

3-(3-(trifluoromethyl)phenyl)propan-1-ol
NMR data is in agreement with the literature reference.[21]
MS (EI): m/z = 186.00, 159.15, 133.10, 117.15, 109.10, 103.10, 91.10, 77.05, 63.10, 51.05

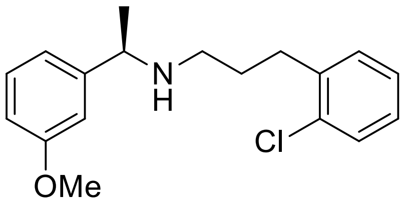

(*R*)-3-(2-chlorophenyl)-*N*-(1-(3-methoxyphenyl)ethyl)propan-1-amine *(tecalcet)*
NMR data is in agreement with the literature reference.[22]
MS (ESI): m/z = 304.15 (M+H)$^+$
$[\alpha]_D^{20}$= +40.2 (c= 1.00, $CHCl_3$) {lit.[23] $[\alpha]_D^{20}$= +39.4 (c= 1.00, $CHCl_3$)}.

3. Collection of NMR Spectra

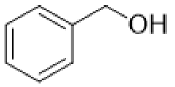

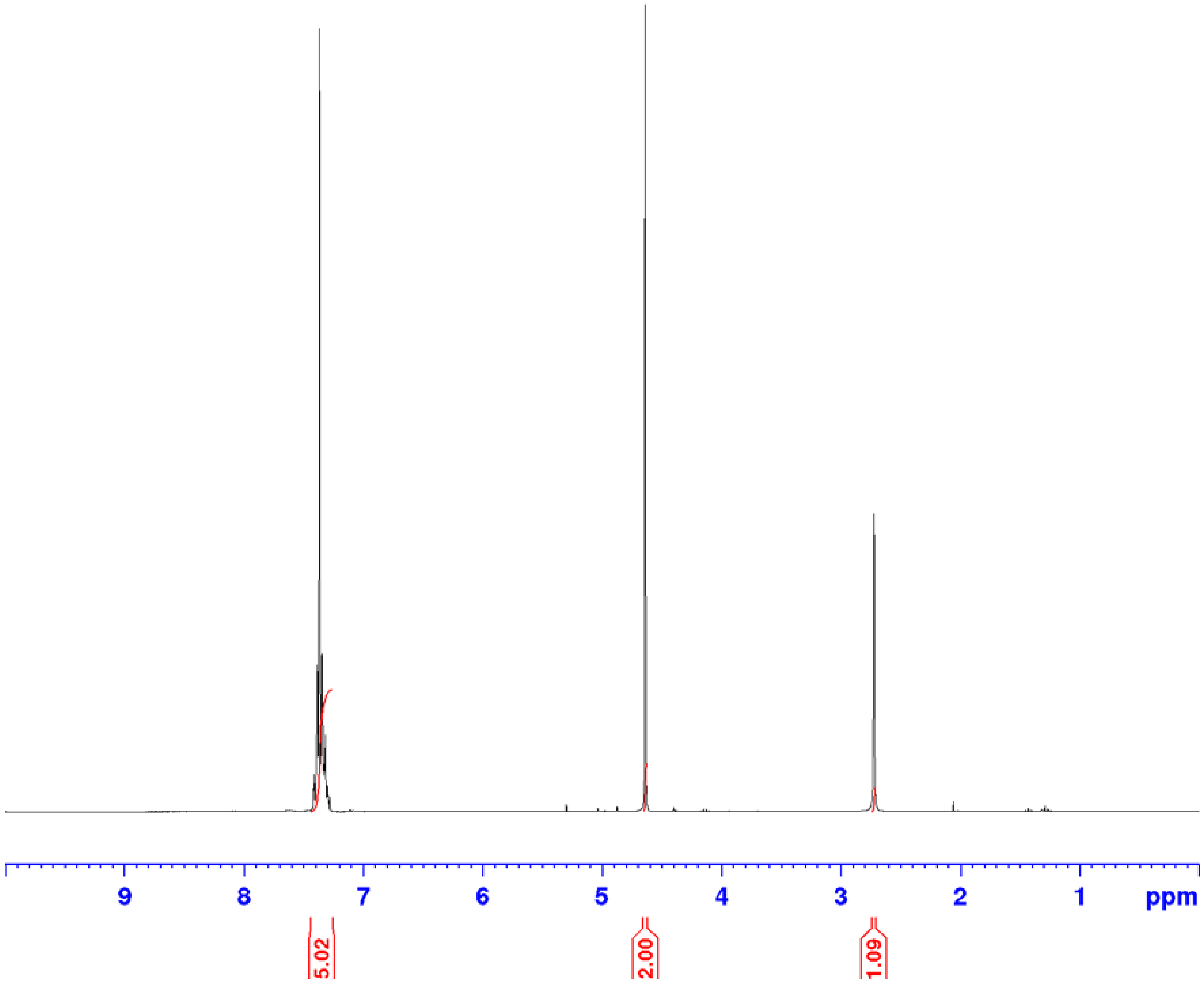

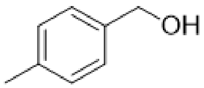

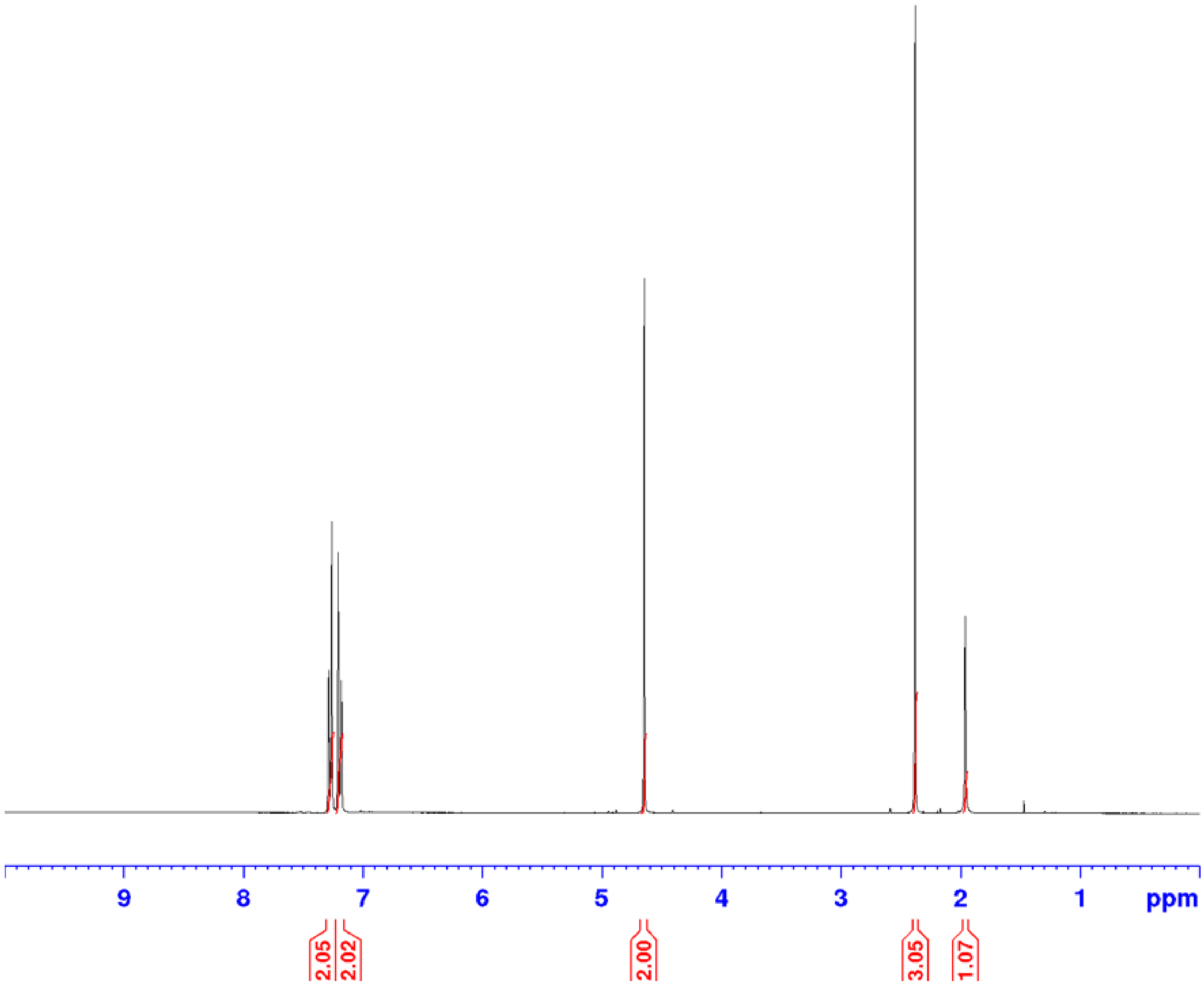

9
8
7
6
5
4
3
2
1
ppm
2.05
2.02
2.00
3.05
1.07

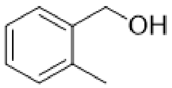
OH

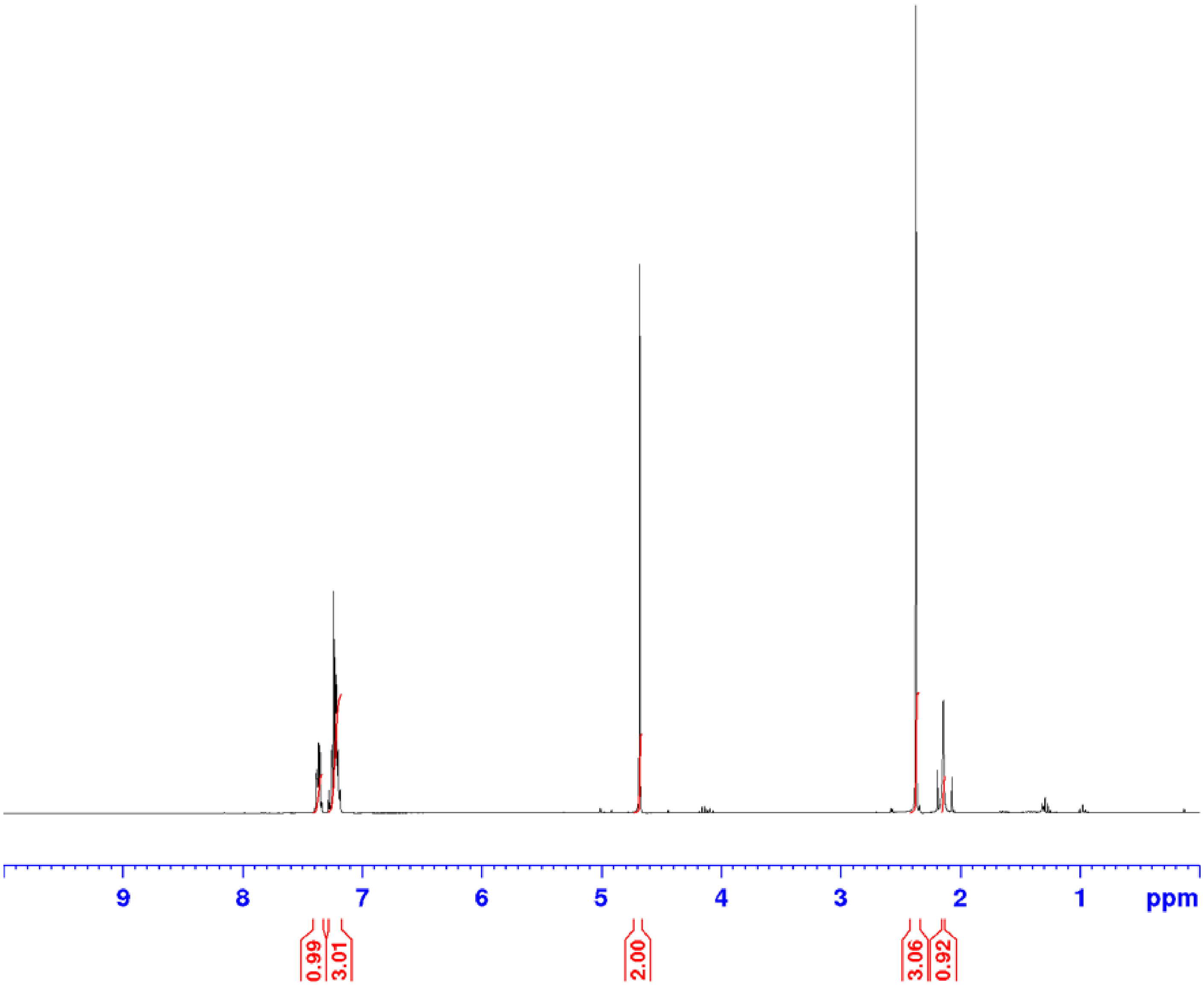
9
8
7
6
5
4
3
2
1
ppm
0.99
3.01
2.00
3.06
0.92

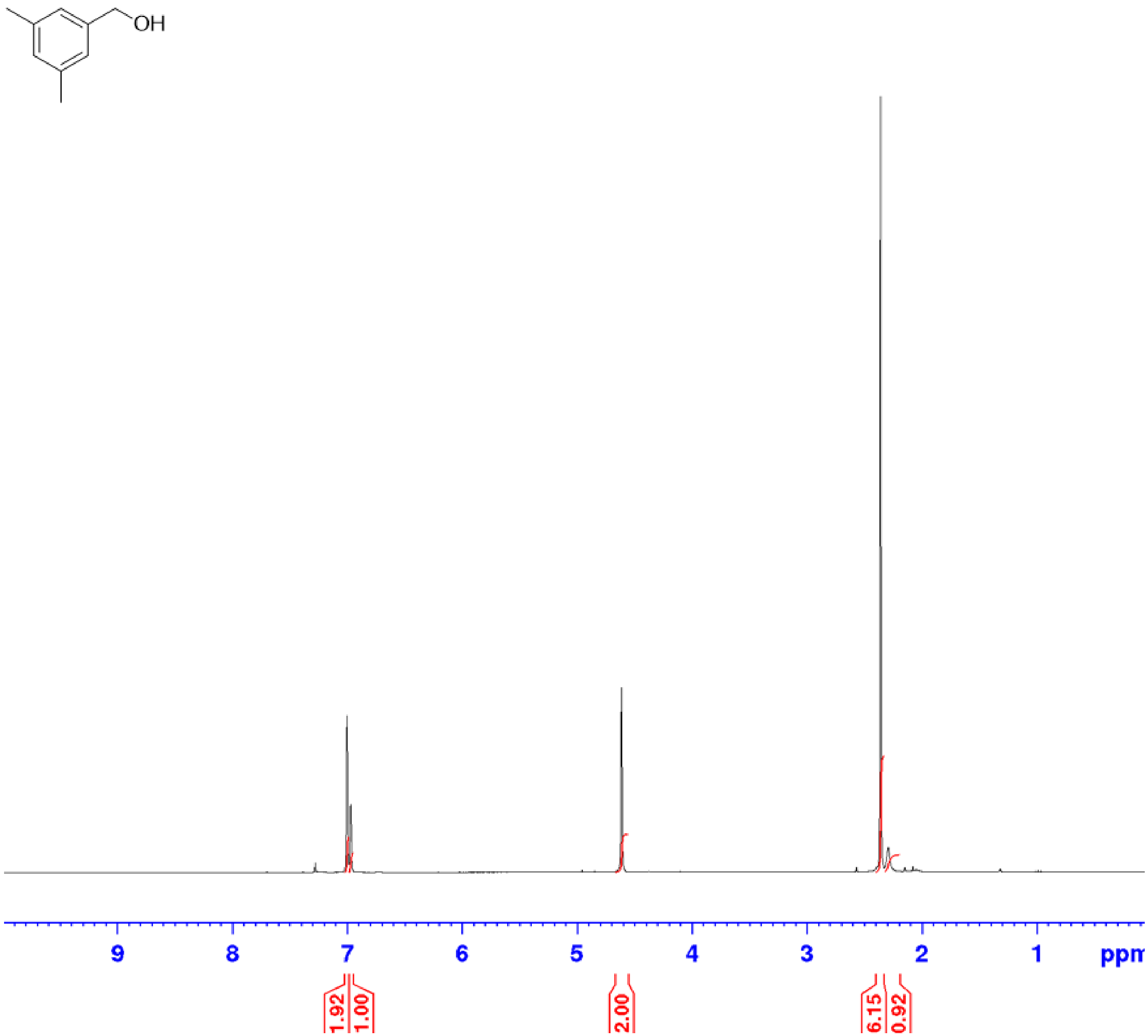
OH
9
8
7
6
5
4
3
2
1
ppm
1.92
1.00
2.00
6.15
0.92

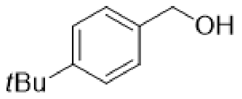
OH
tBu

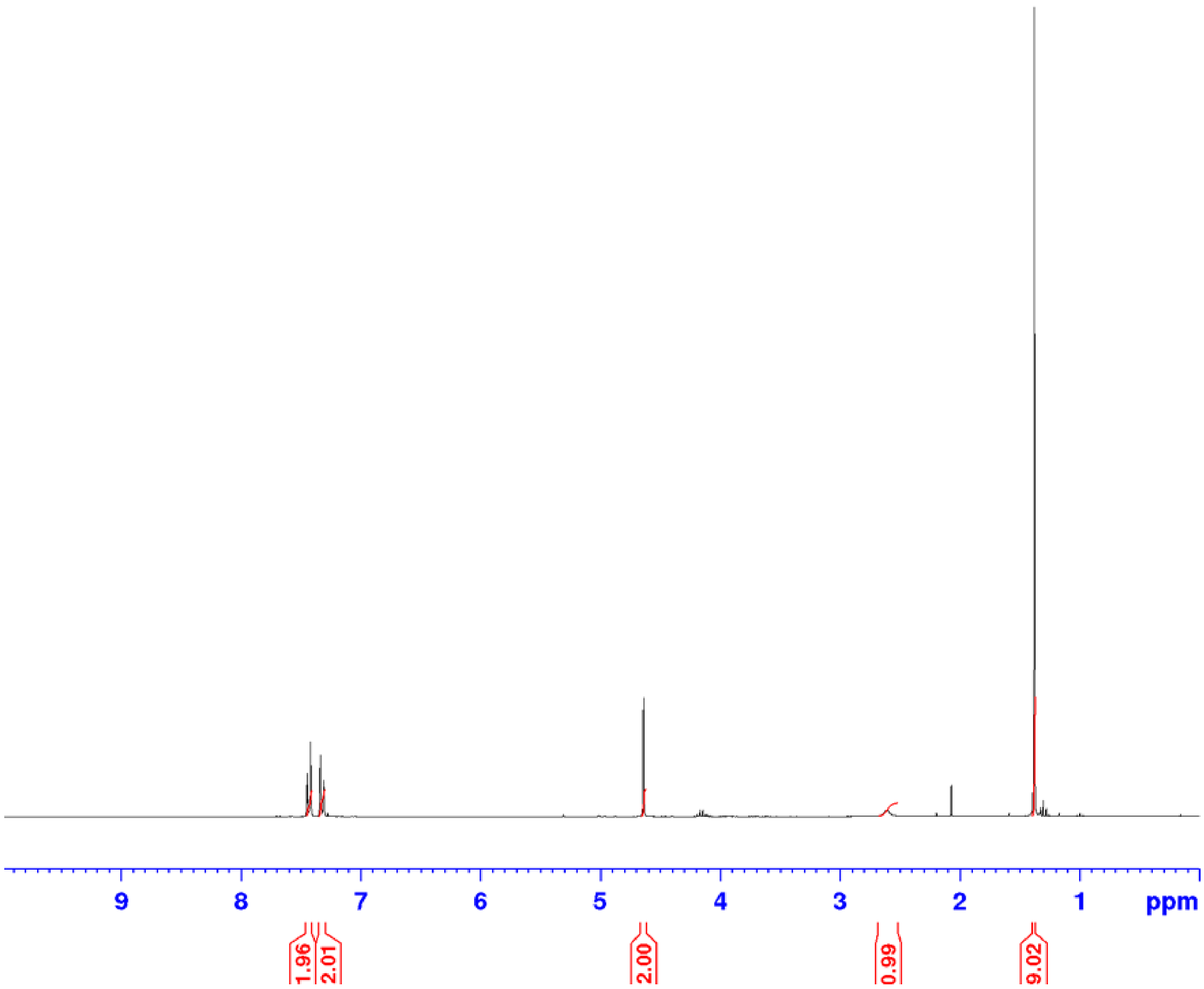
9
8
7
6
5
4
3
2
1
ppm
1.96
2.01
2.00
0.99
9.02

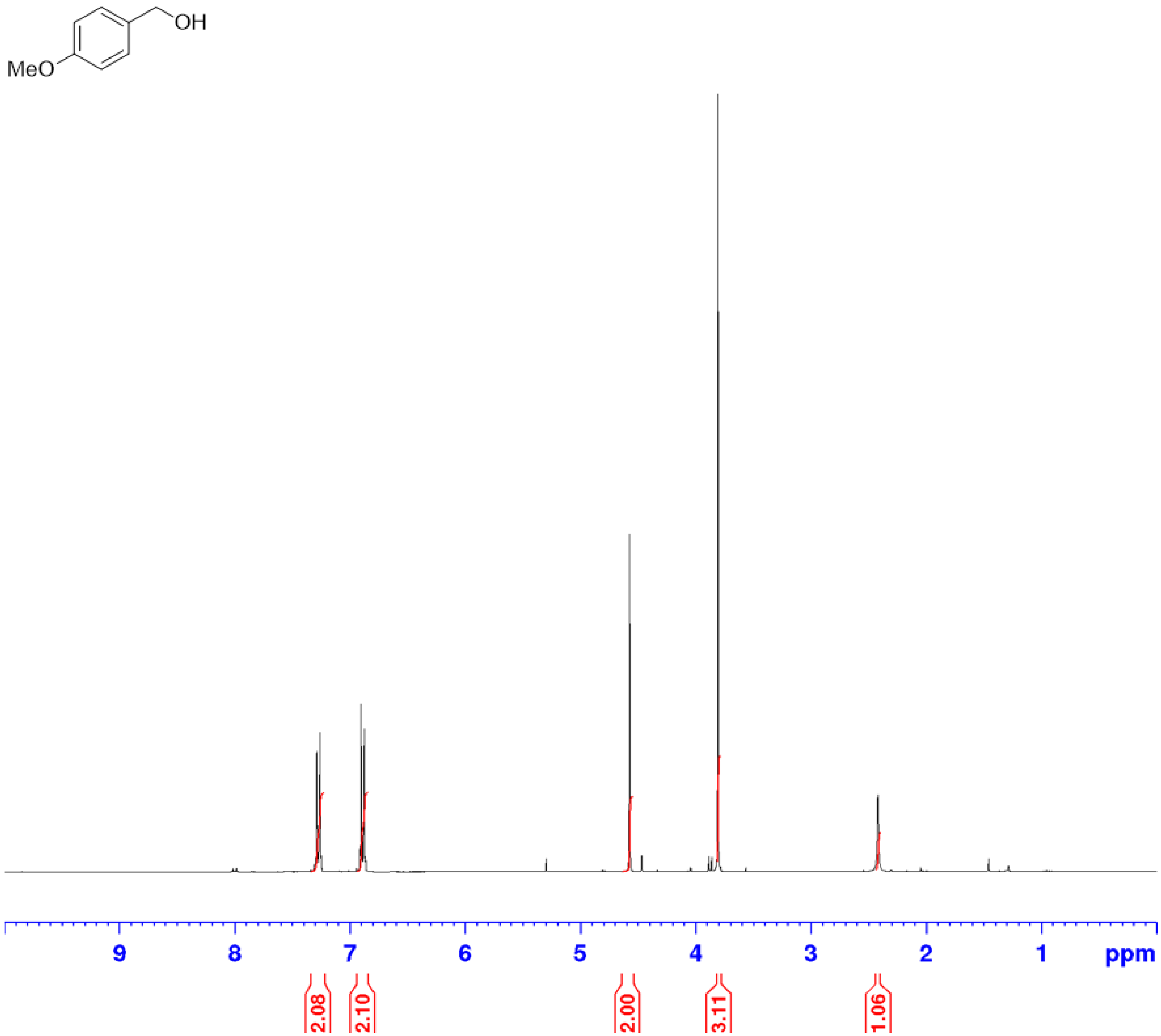
MeO
OH
9
8
7
6
5
4
3
2
1
ppm
2.08
2.10
2.00
3.11
1.06

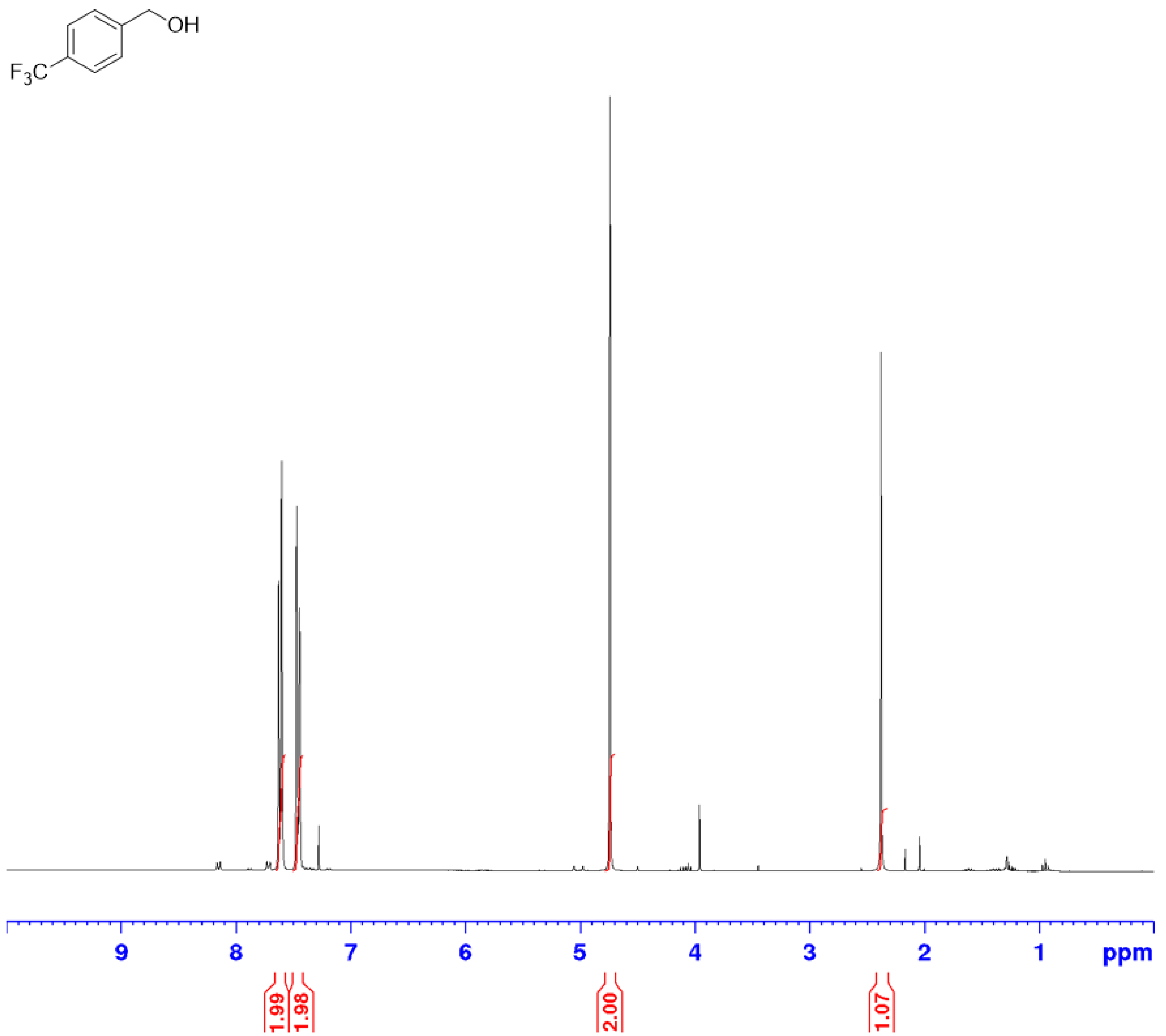
OH
F3C
9
8
7
6
5
4
3
2
1
ppm
1.99
1.98
2.00
1.07

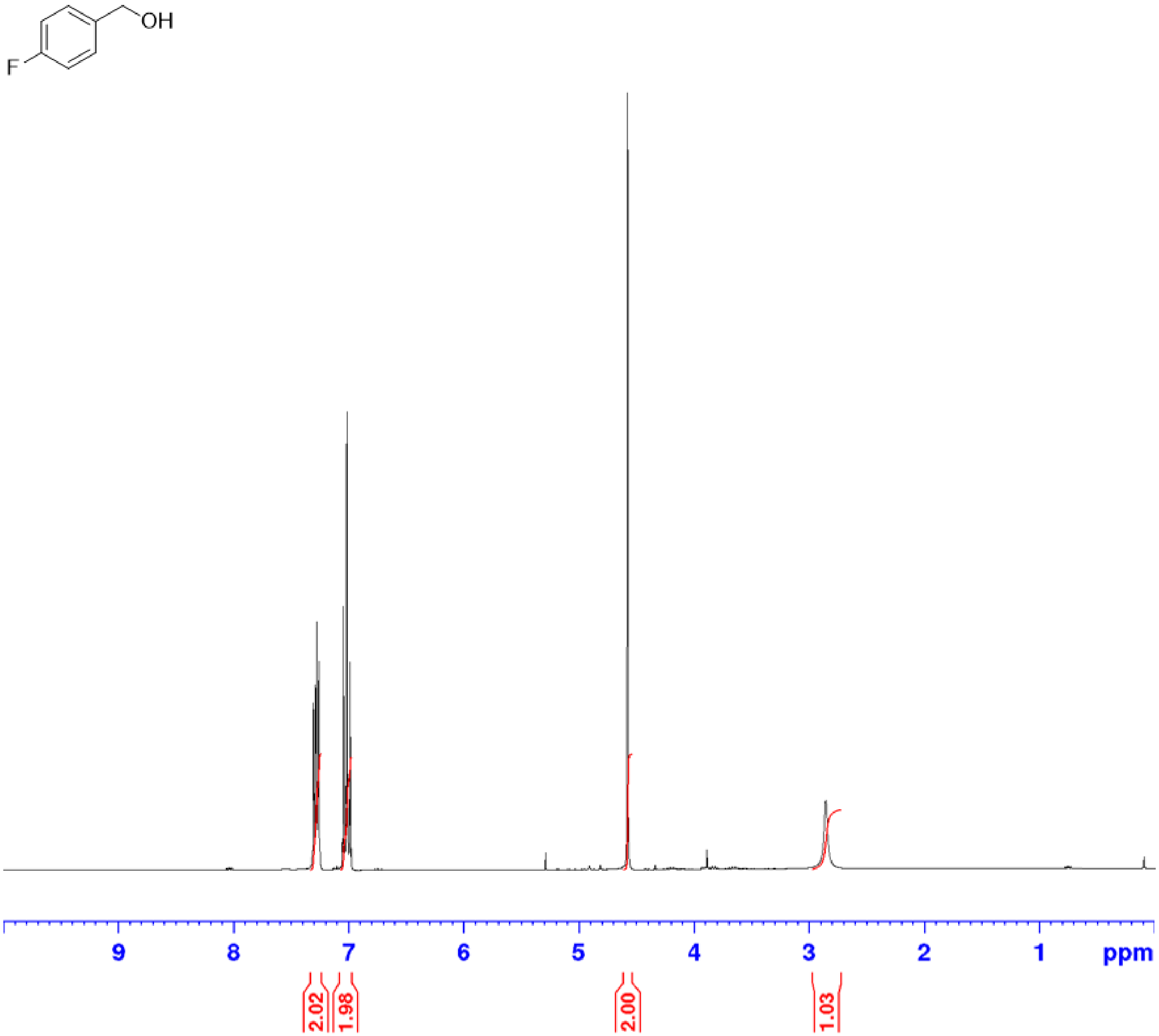

OH
F
9
8
7
6
5
4
3
2
1
ppm
2.02
1.98
2.00
1.03

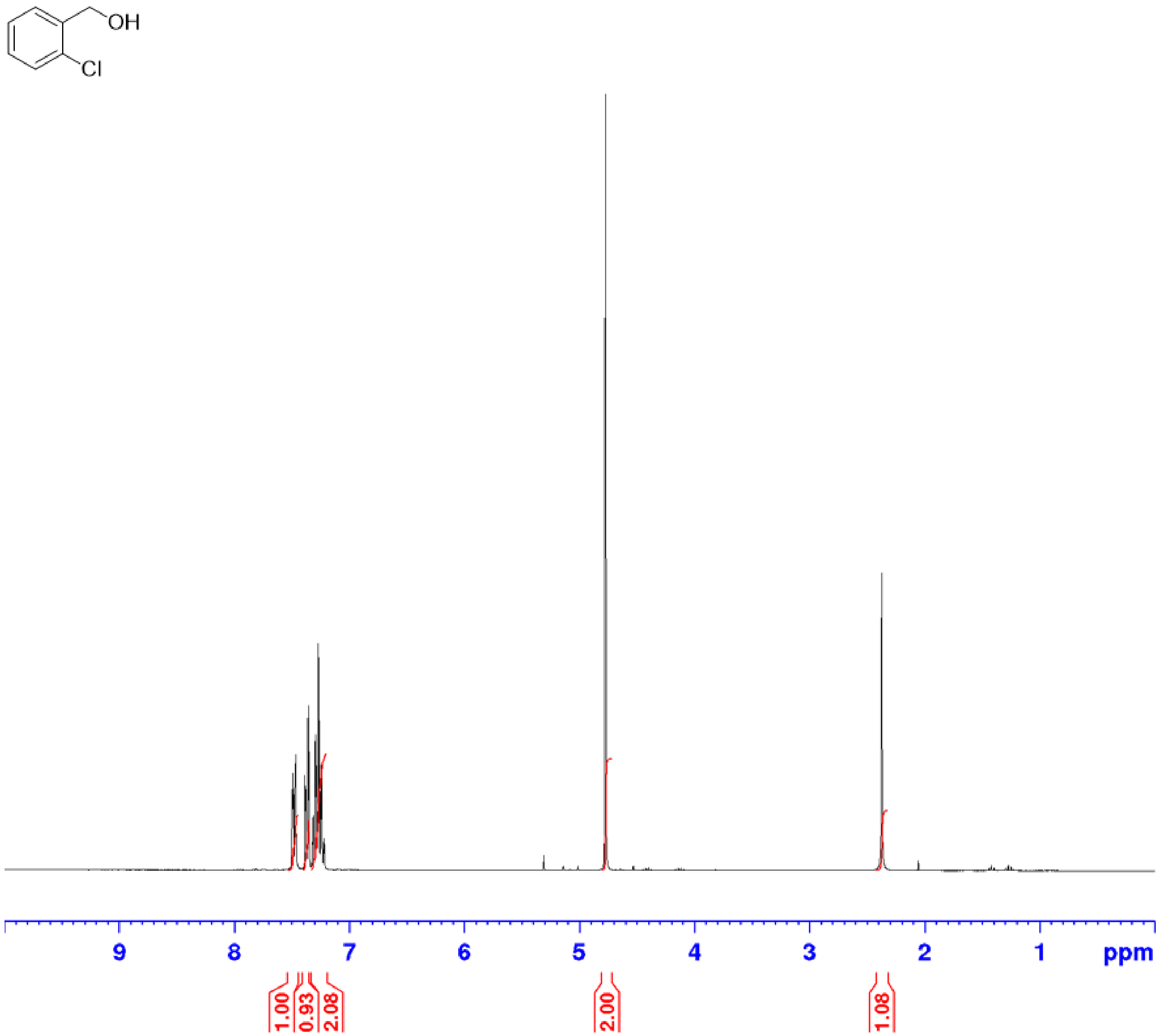

OH
Cl
9
8
7
6
5
4
3
2
1
ppm
1.00
0.93
2.08
2.00
1.08

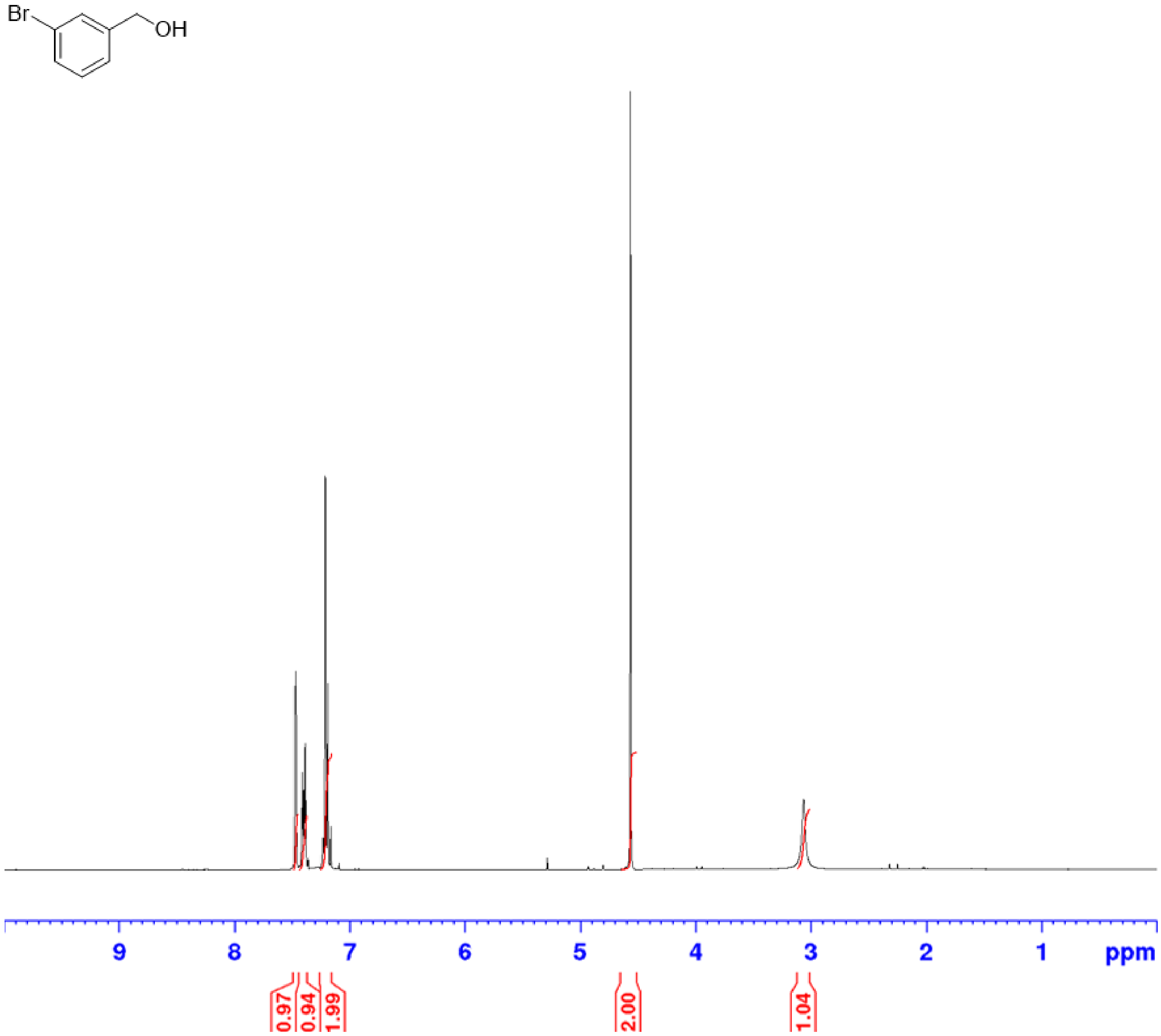
Br
OH
9
8
7
6
5
4
3
2
1
ppm
0.97
0.94
1.99
2.00
1.04

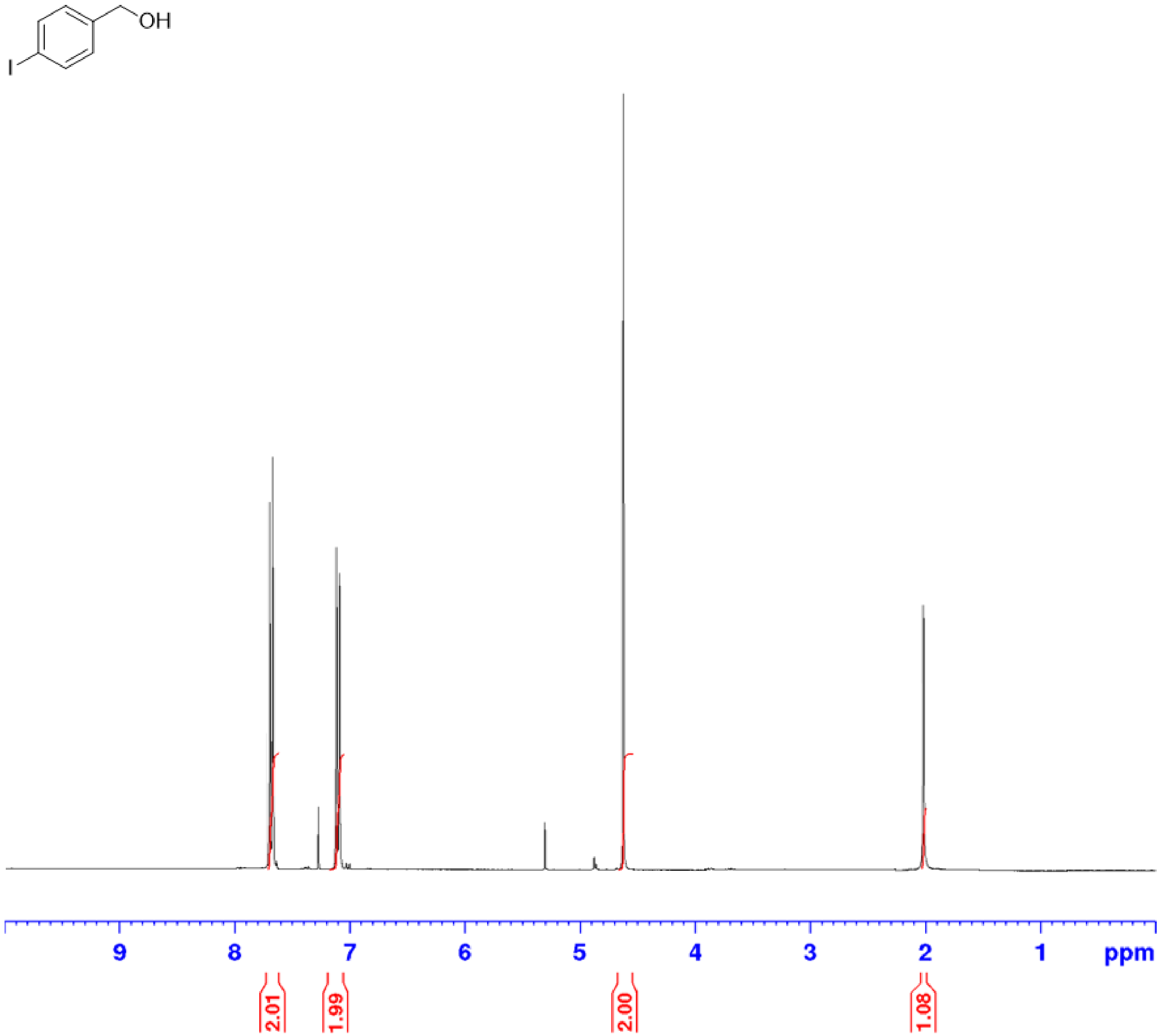
OH
I
9
8
7
6
5
4
3
2
1
ppm
2.01
1.99
2.00
1.08

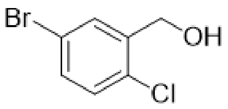
Br
OH
Cl

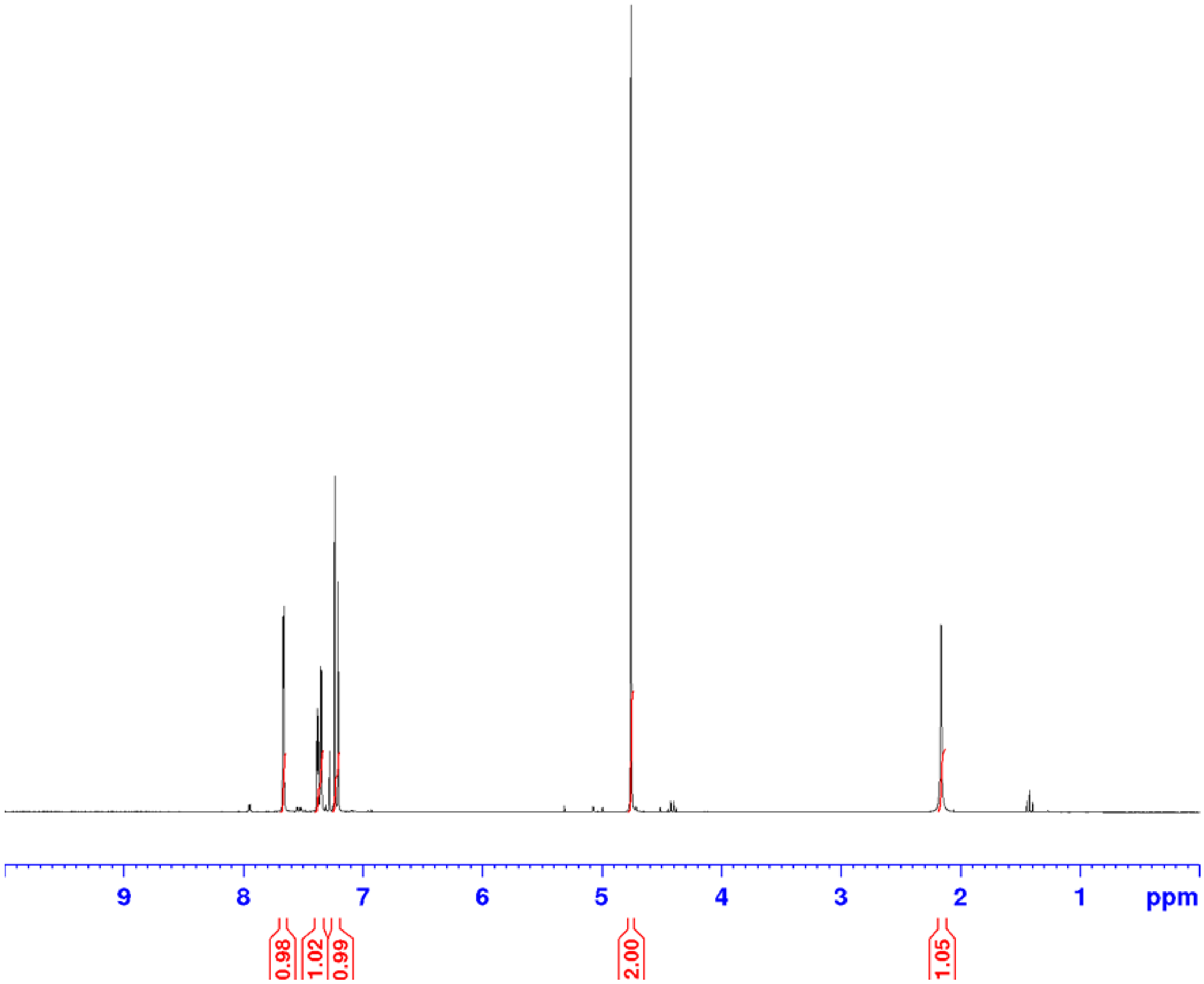
9
8
7
6
5
4
3
2
1
ppm
0.98
1.02
0.99
2.00
1.05

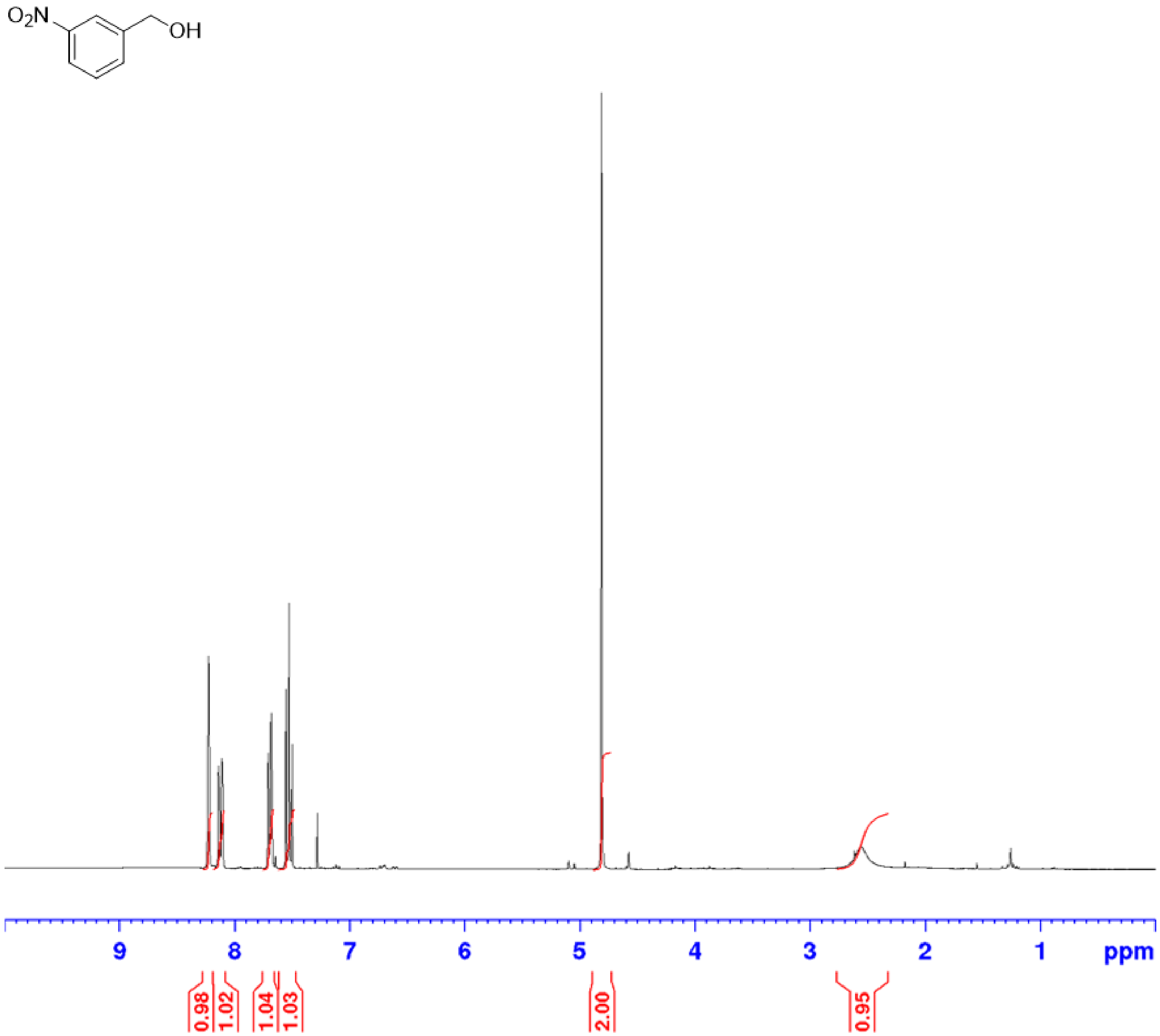
O2N
OH
9
8
7
6
5
4
3
2
1
ppm
0.98
1.02
1.04
1.03
2.00
0.95

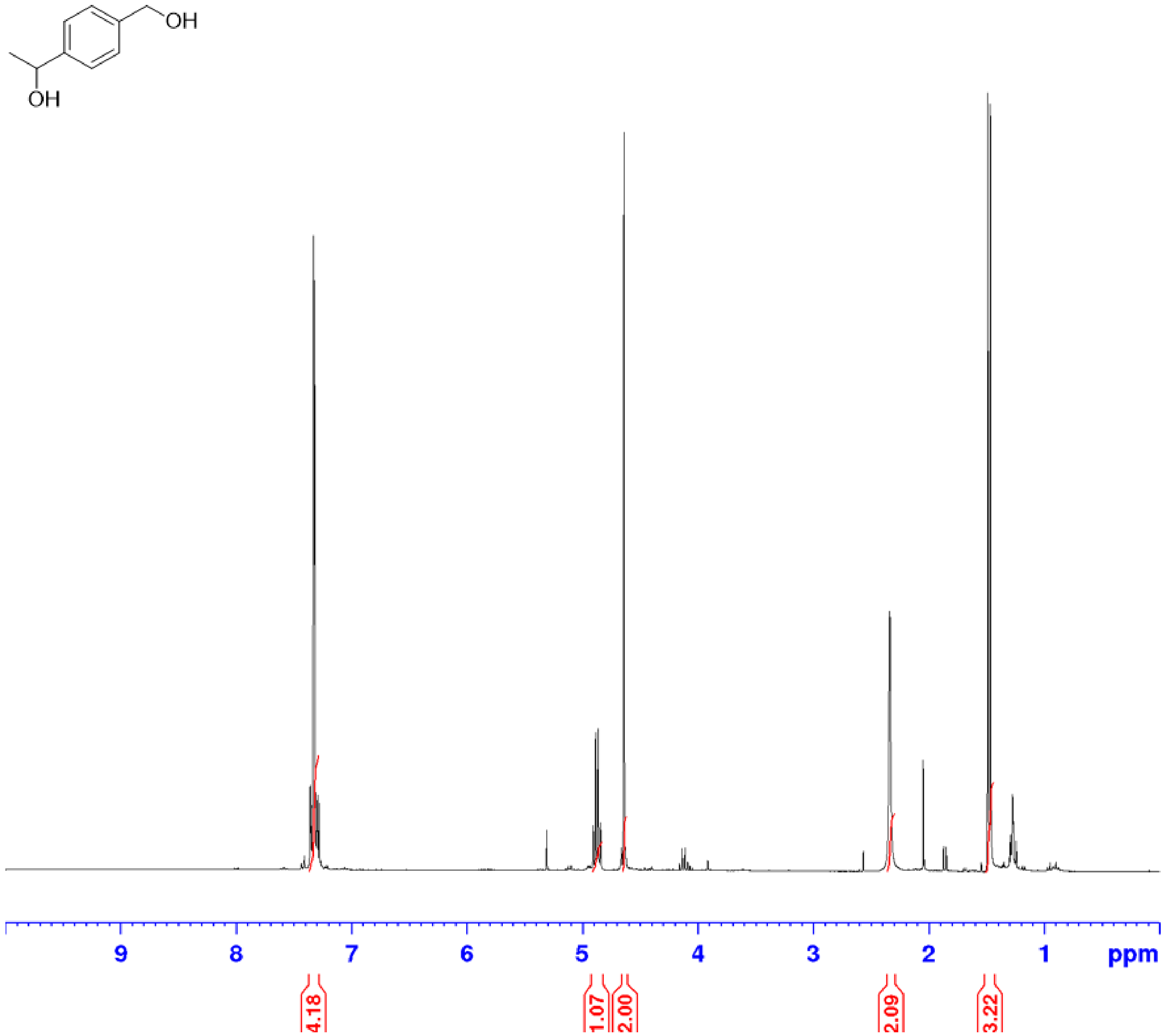
OH
OH
9
8
7
6
5
4
3
2
1
ppm
4.18
1.07
2.00
2.09
3.22

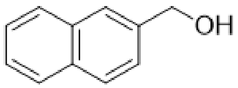
OH

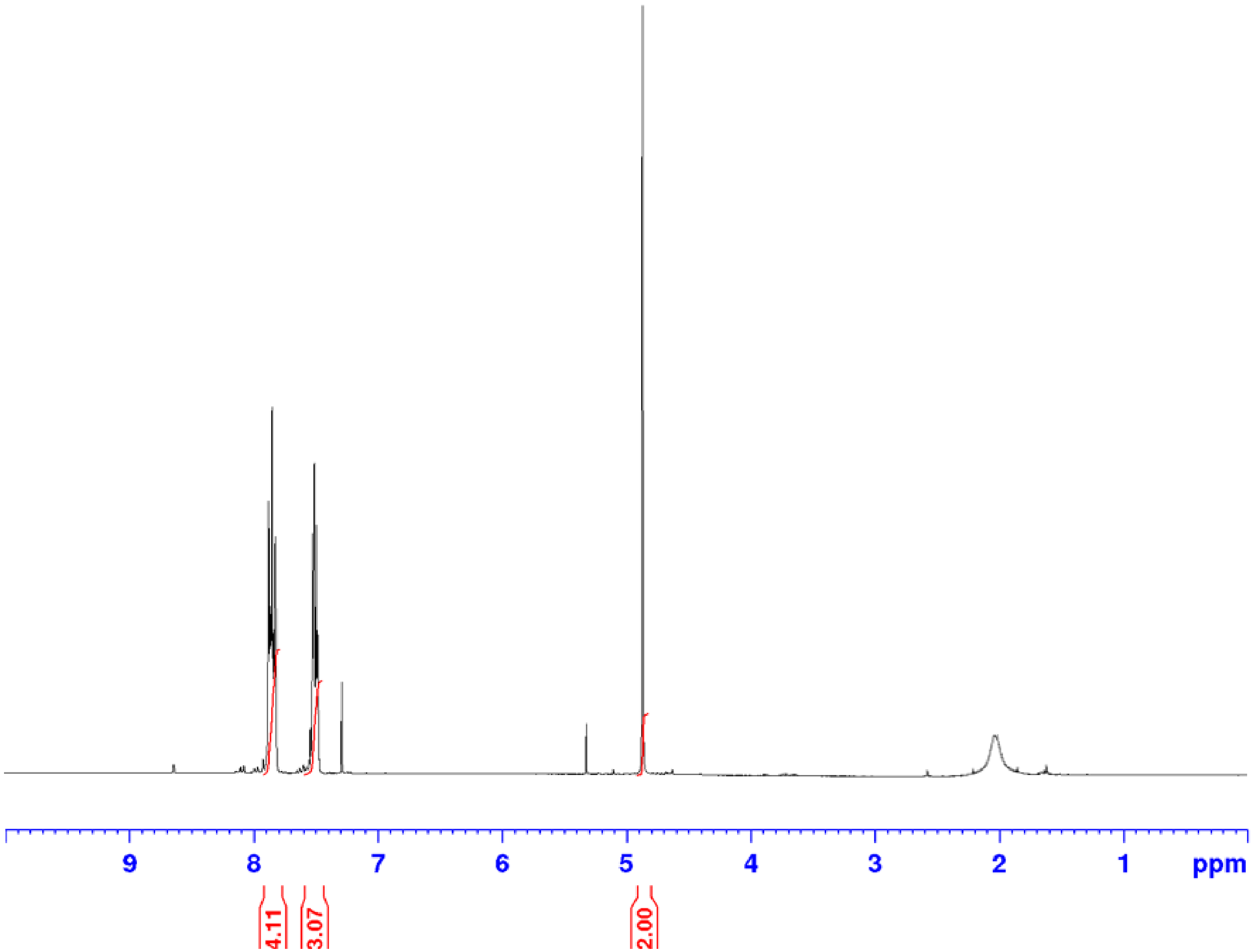
9
8
7
6
5
4
3
2
1
ppm
4.11
3.07
2.00

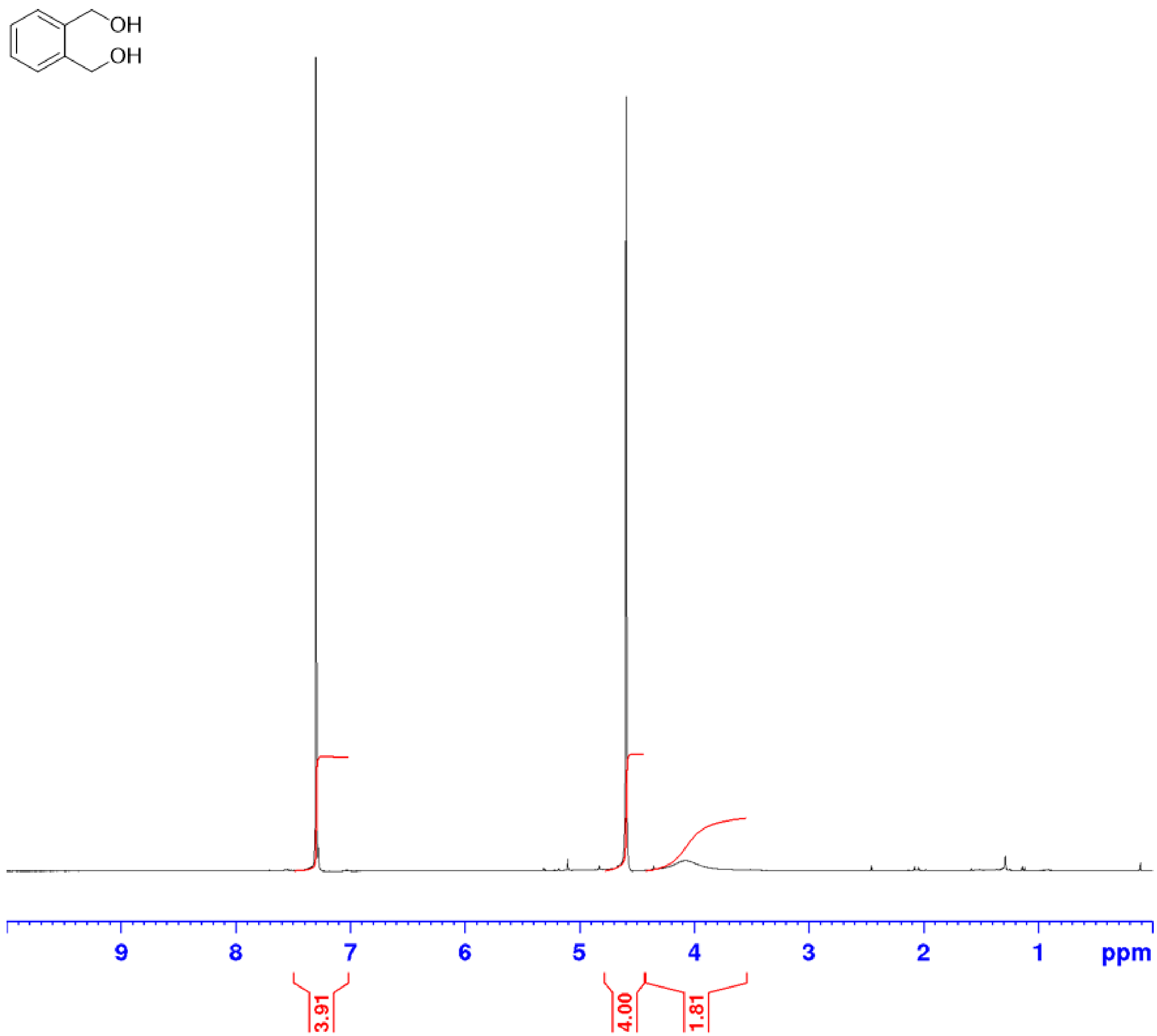
OH
OH
9
8
7
6
5
4
3
2
1
ppm
3.91
4.00
1.81

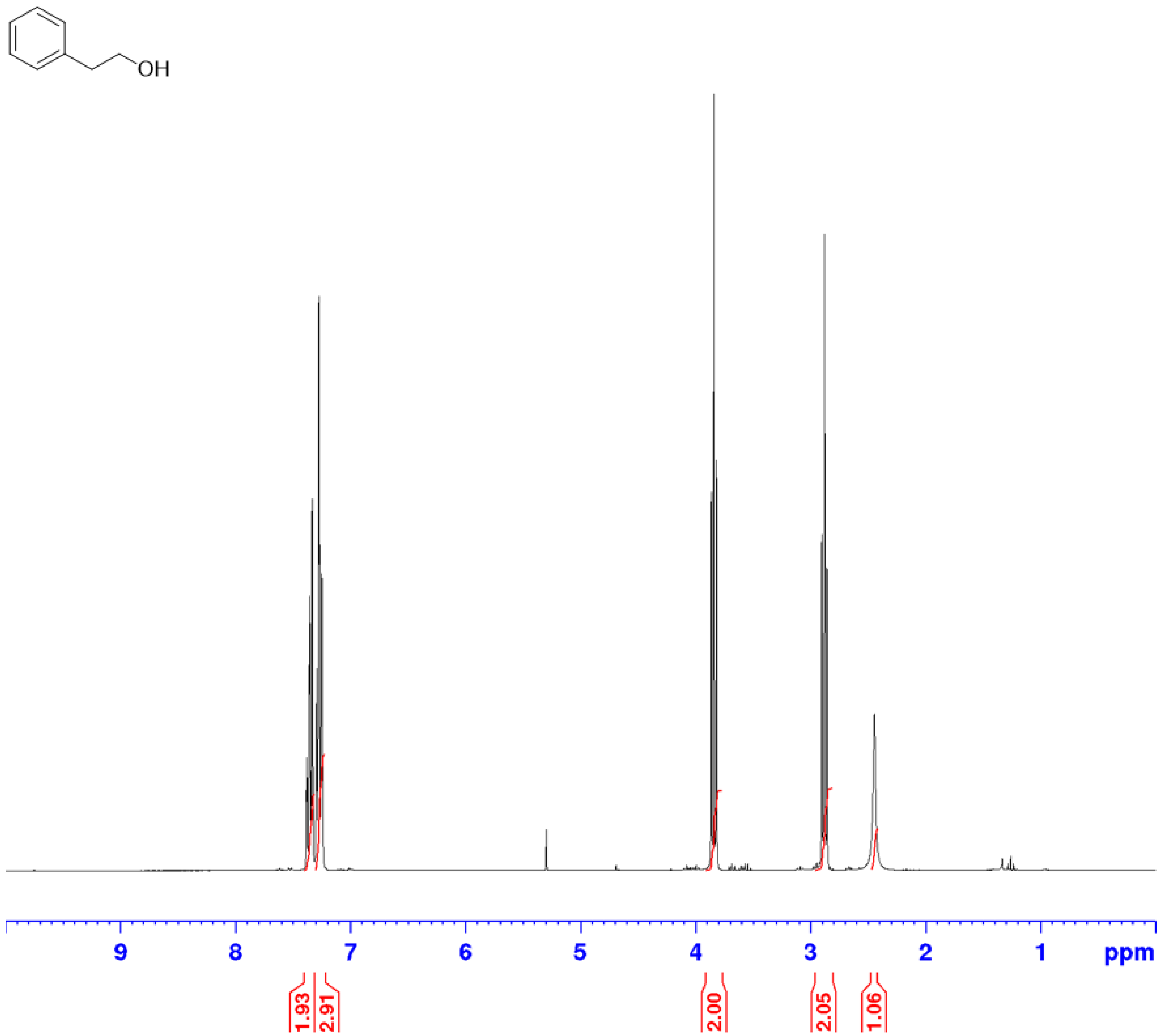
OH
9
8
7
6
5
4
3
2
1
ppm
1.93
2.91
2.00
2.05
1.06

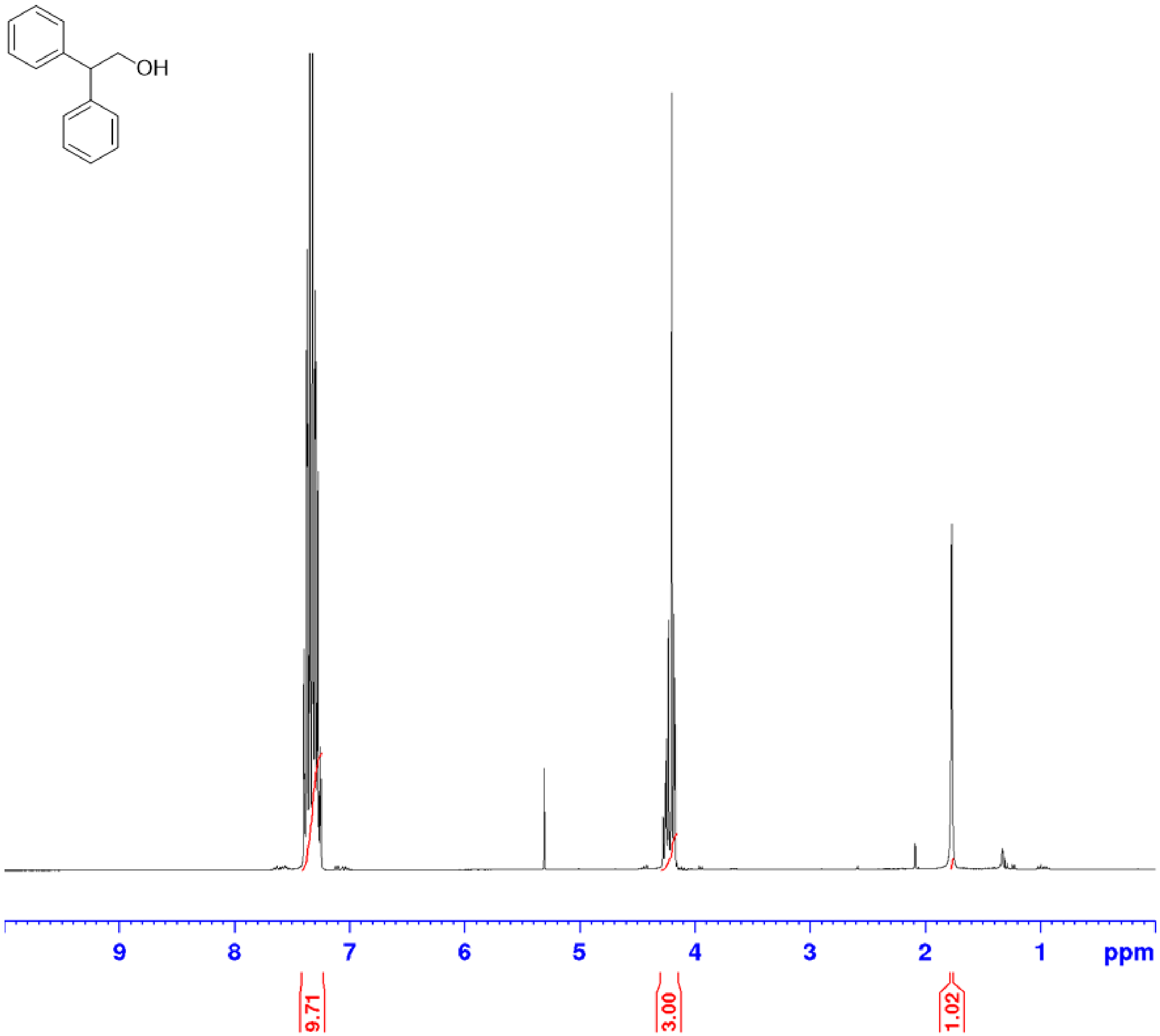
OH
9
8
7
6
5
4
3
2
1
ppm
9.71
3.00
1.02

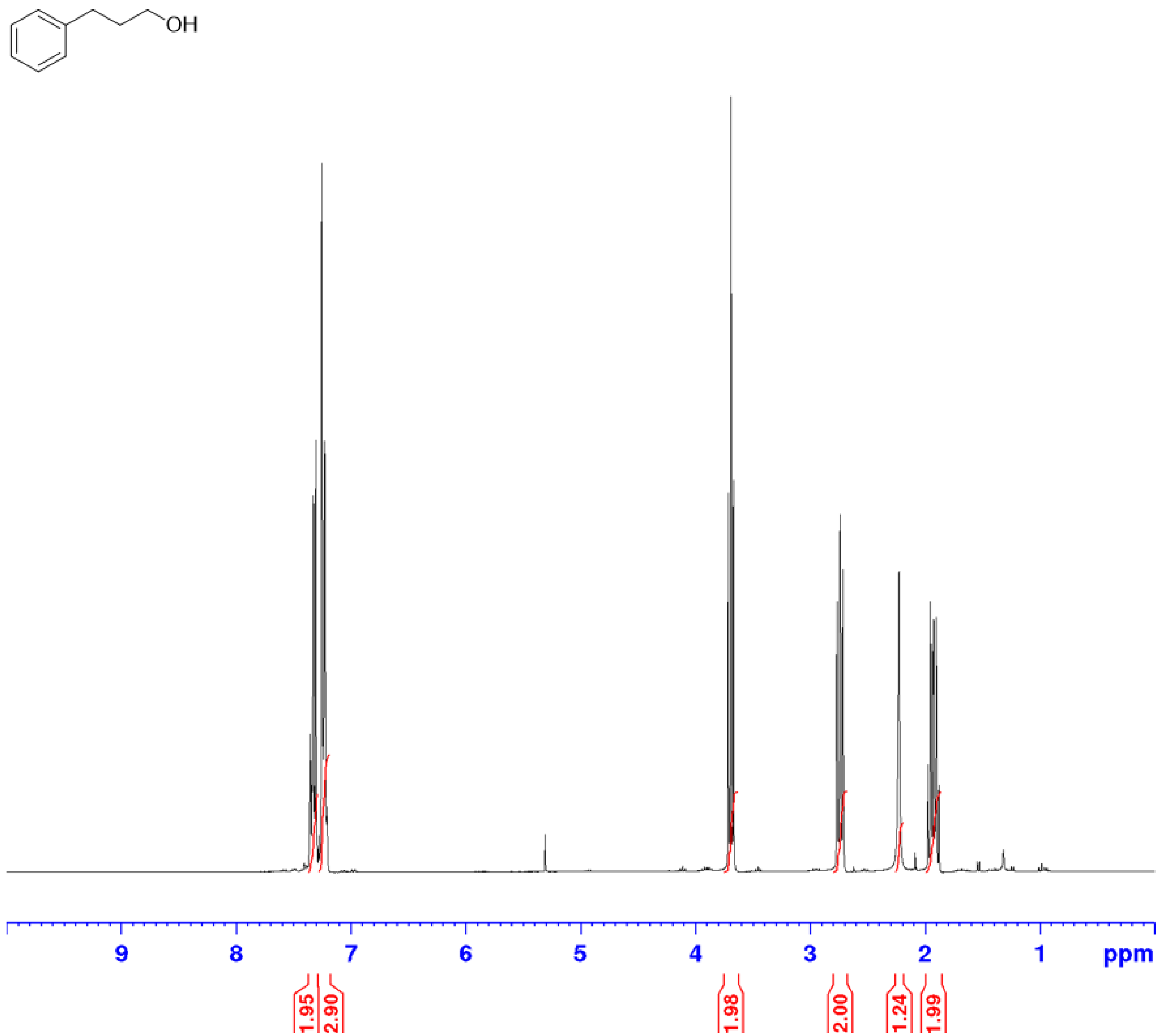
OH
9
8
7
6
5
4
3
2
1
ppm
1.95
2.90
1.98
2.00
1.24
1.99

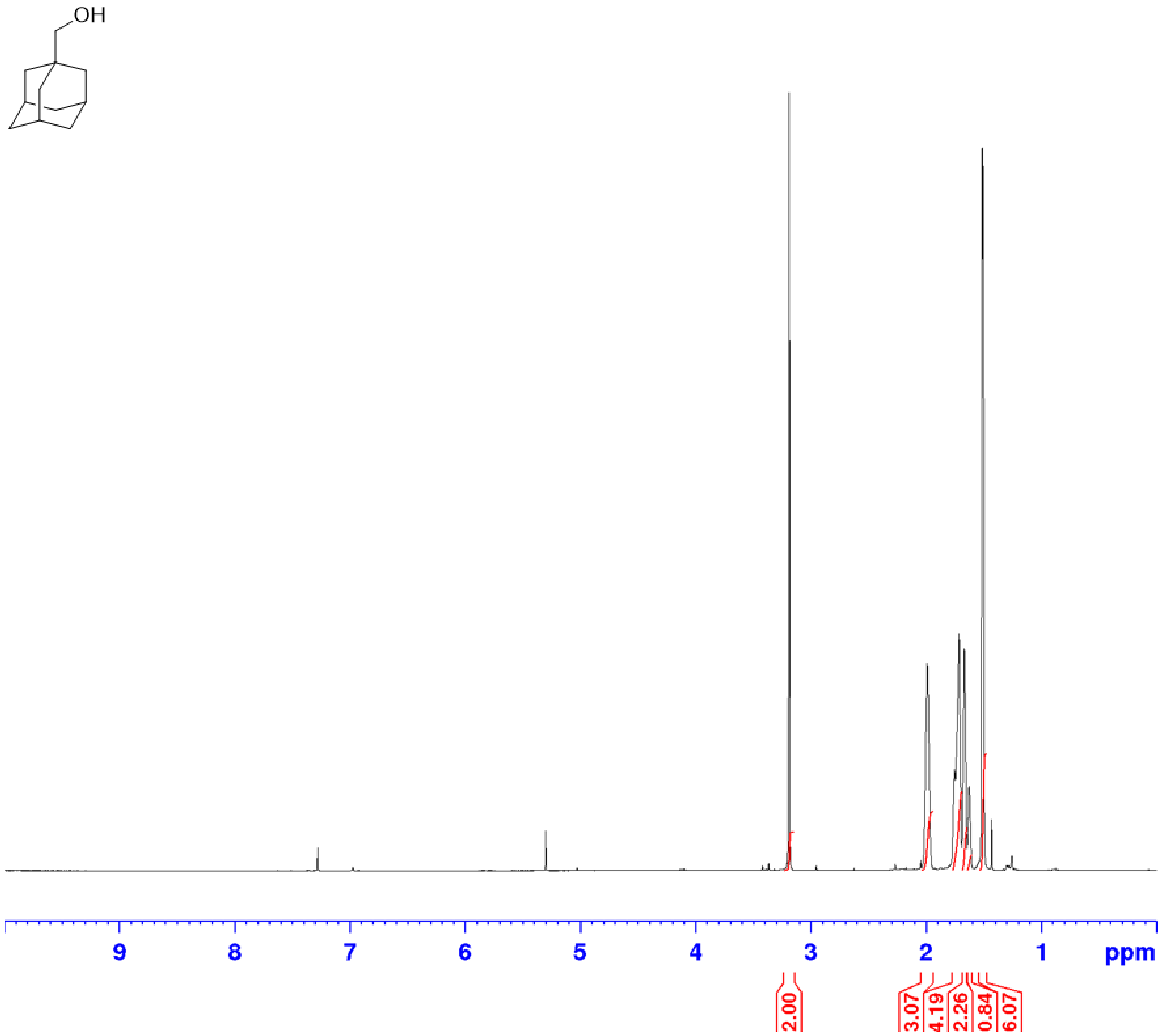
OH
9
8
7
6
5
4
3
2
1
ppm
2.00
3.07
4.19
2.26
0.84
6.07

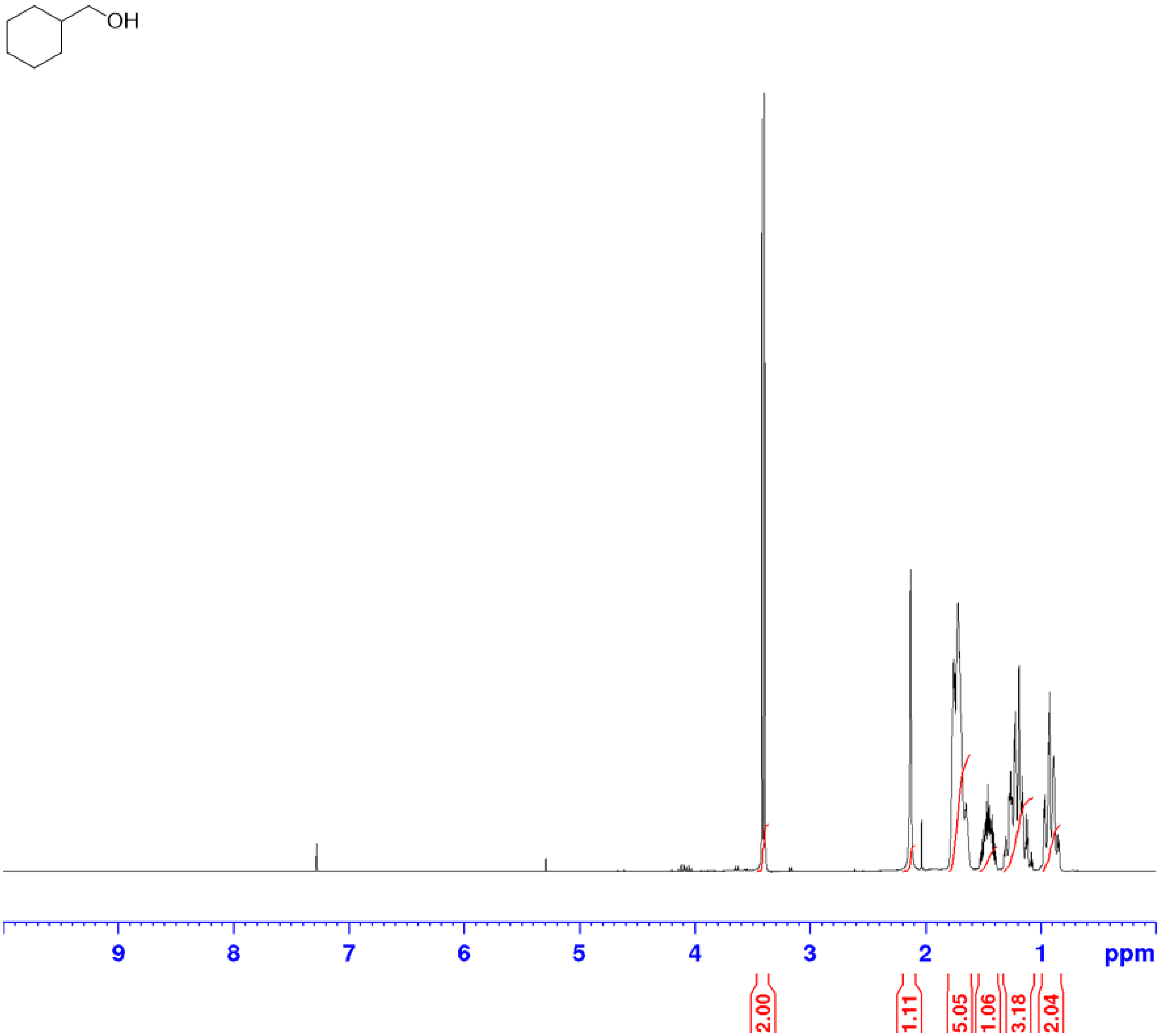
OH
9
8
7
6
5
4
3
2
1
ppm
2.00
1.11
5.05
1.06
3.18
2.04

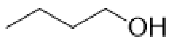

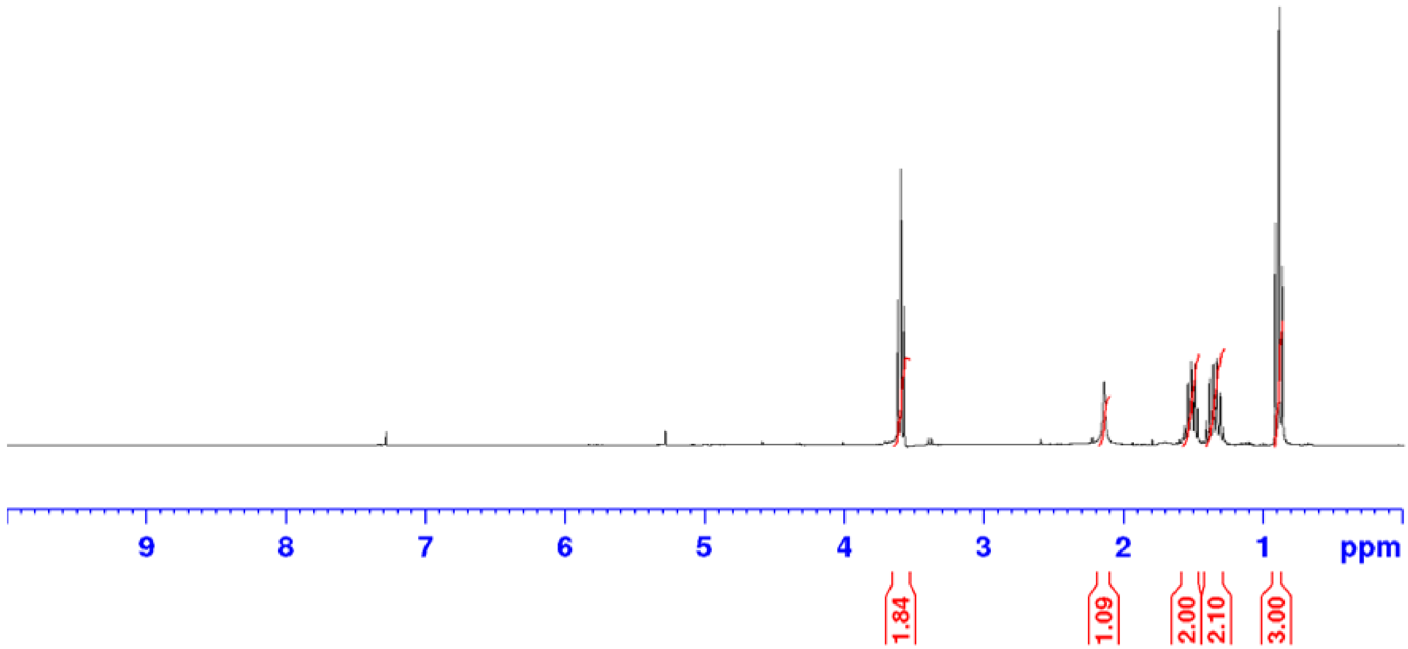
9
8
7
6
5
4
3
2
1
ppm
1.84
1.09
2.00
2.10
3.00

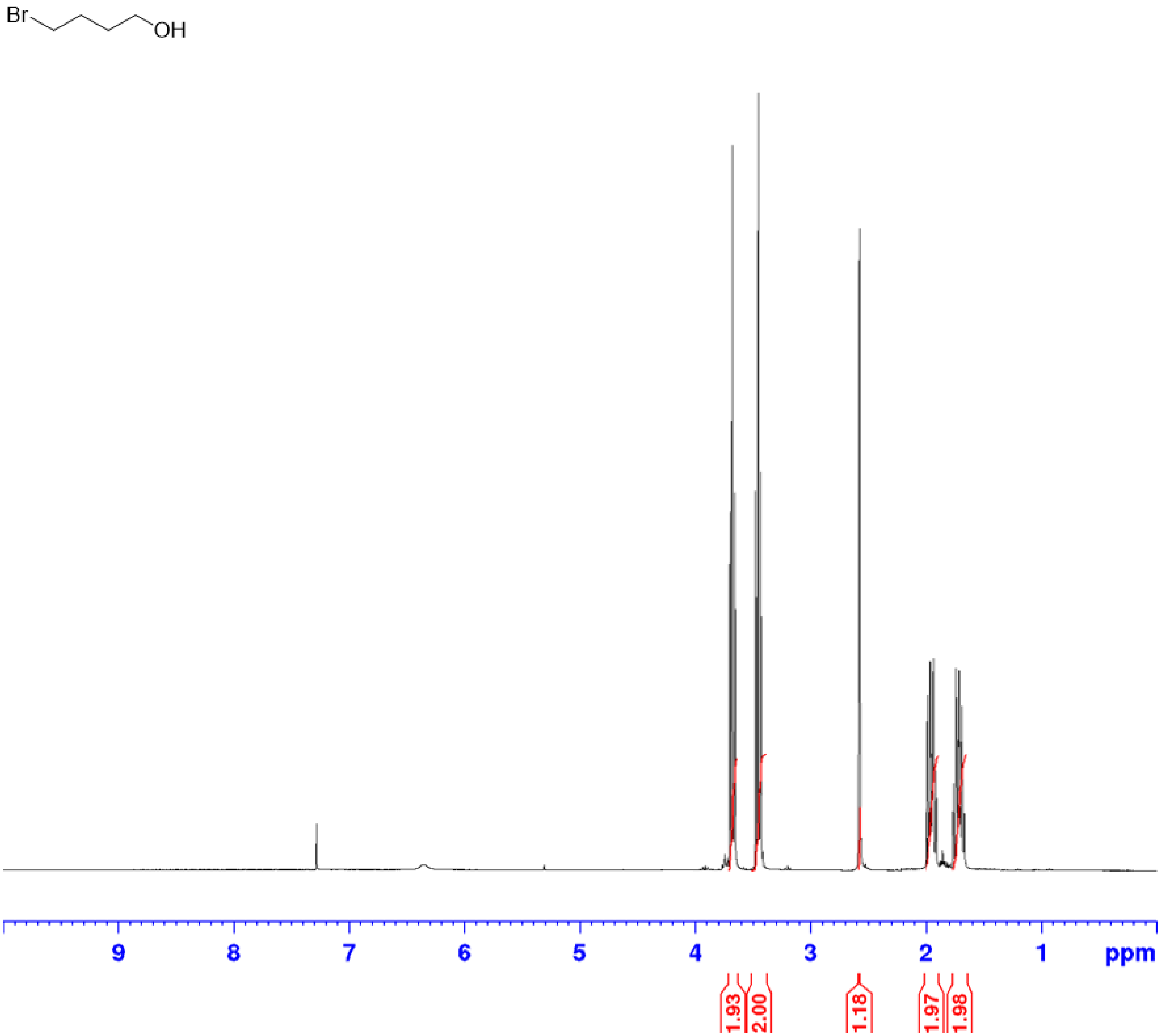

Br
OH
9
8
7
6
5
4
3
2
1
ppm
1.93
2.00
1.18
1.97
1.98

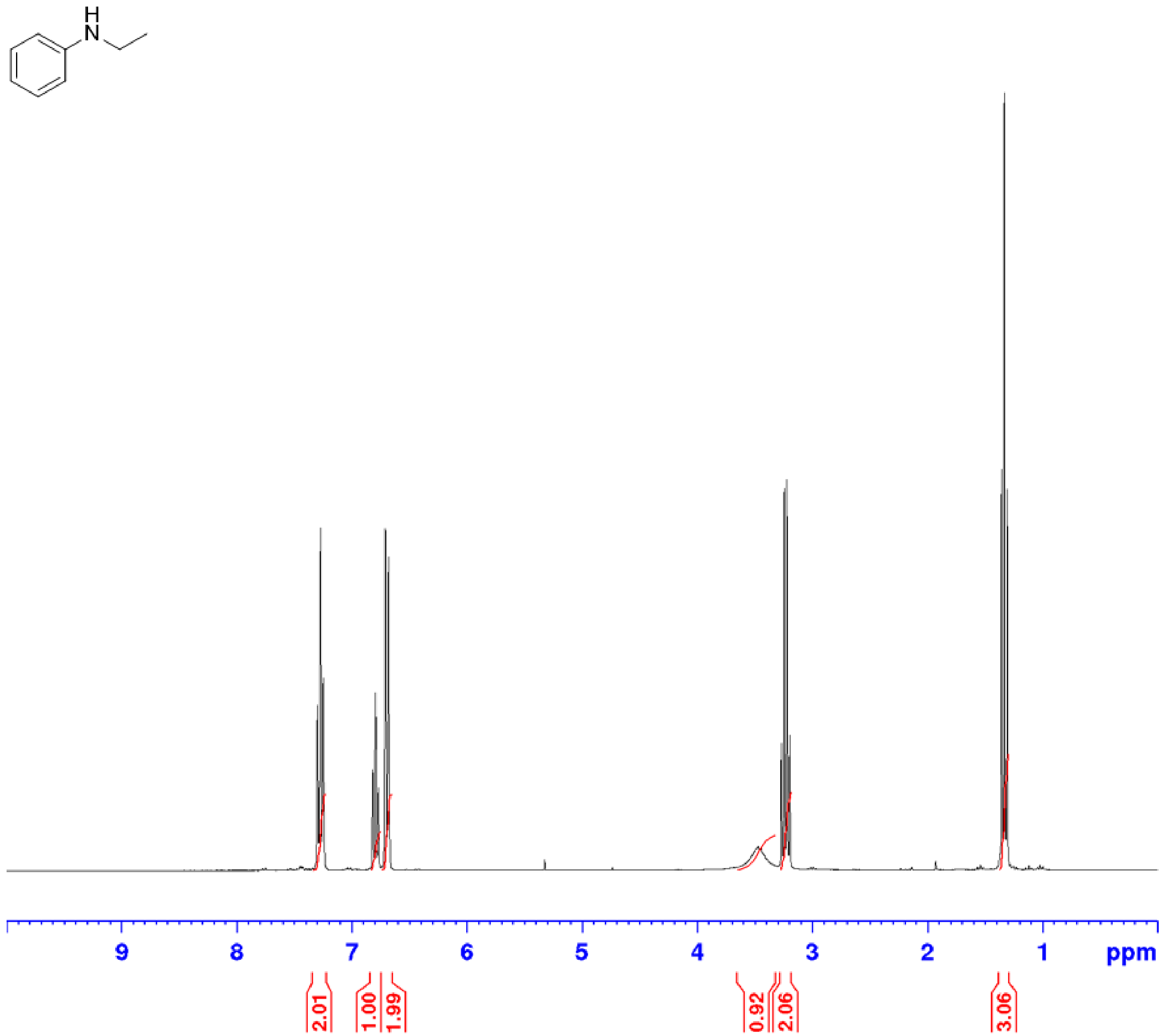

H
N
9
8
7
6
5
4
3
2
1
ppm
2.01
1.00
1.99
0.92
2.06
3.06

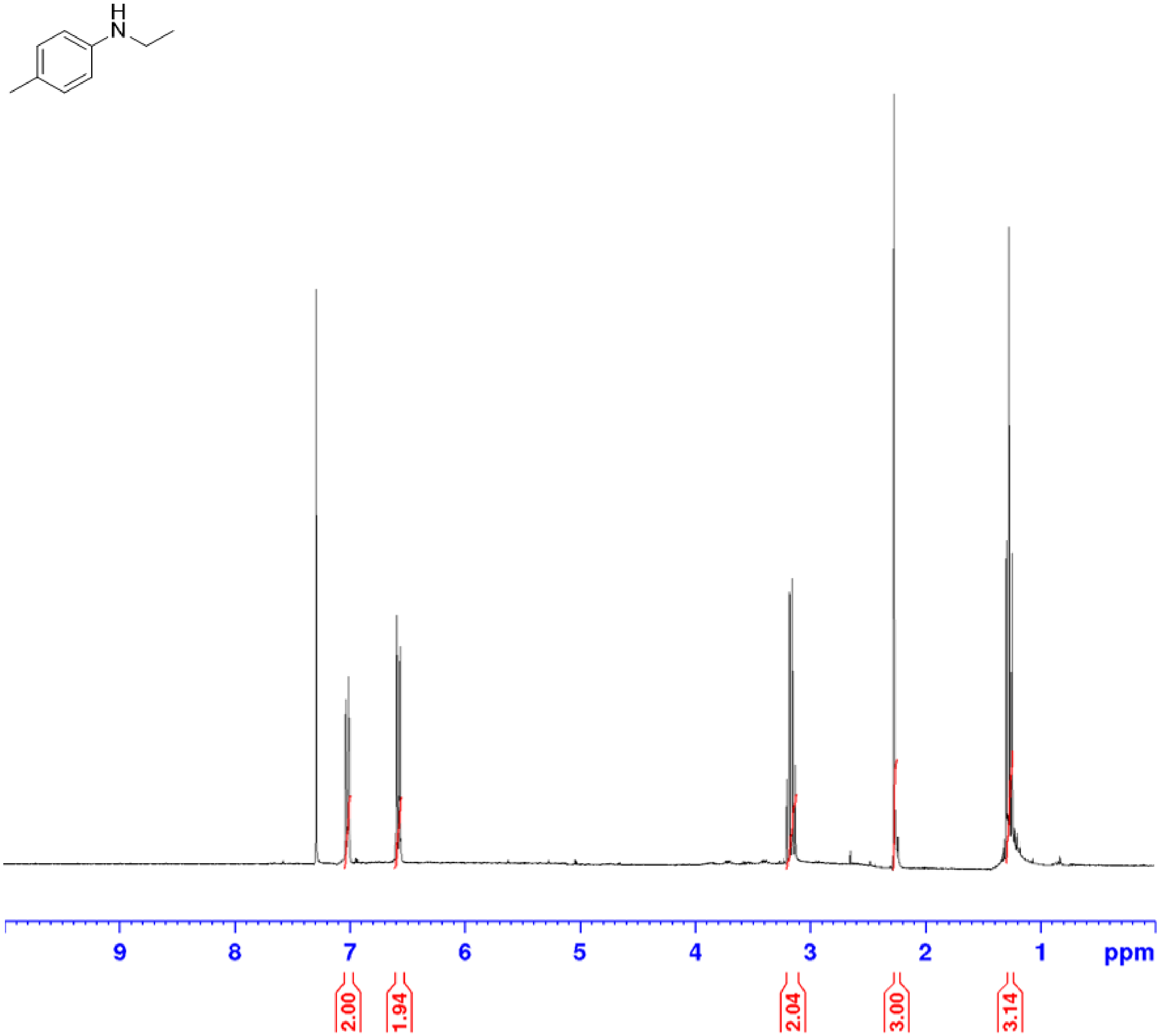
H
N
9
8
7
6
5
4
3
2
1
ppm
2.00
1.94
2.04
3.00
3.14

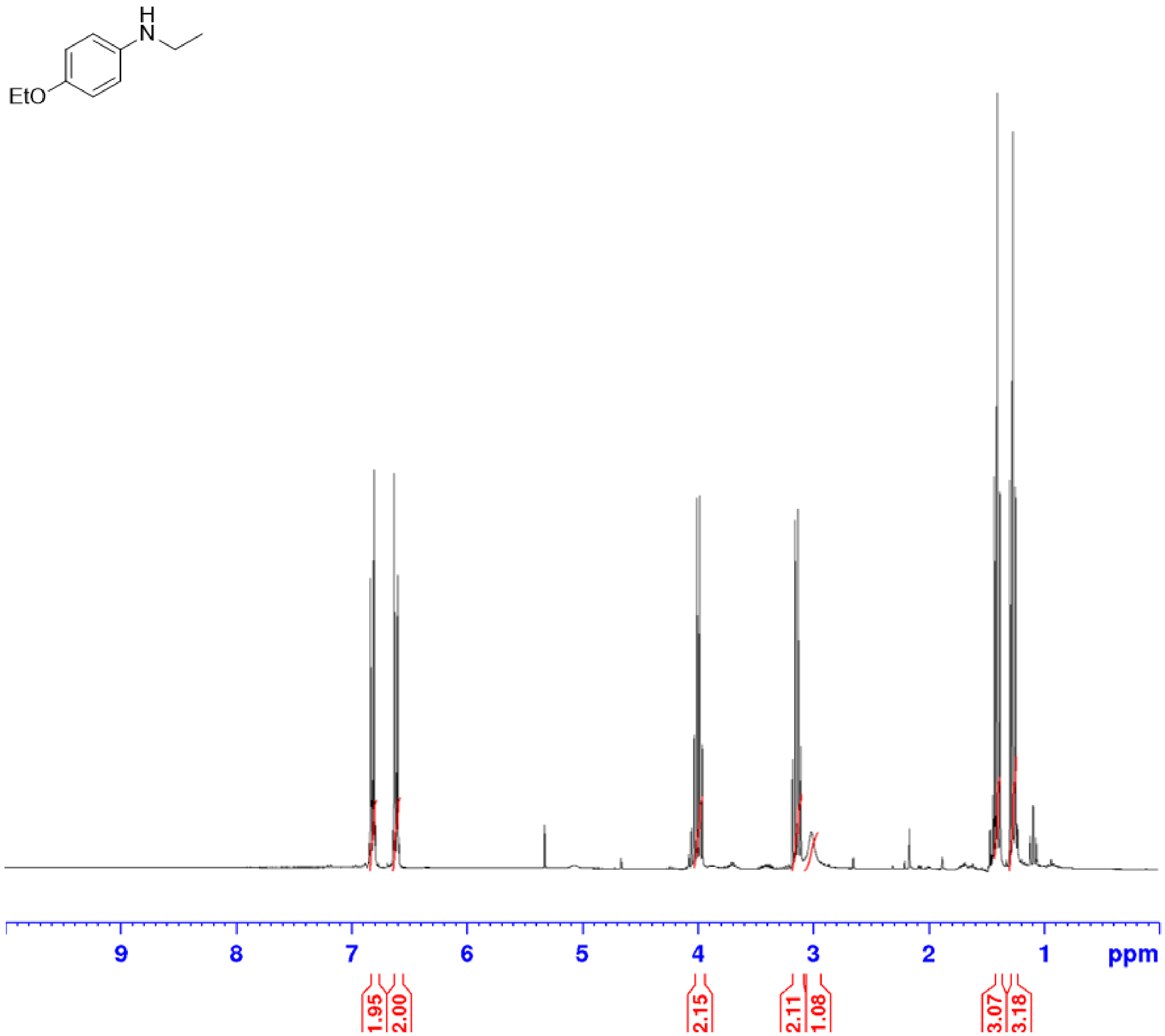
EtO
H
N
9
8
7
6
5
4
3
2
1
ppm
1.95
2.00
2.15
2.11
1.08
3.07
3.18

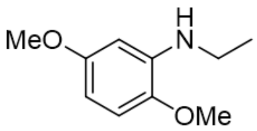
MeO
H
N
OMe

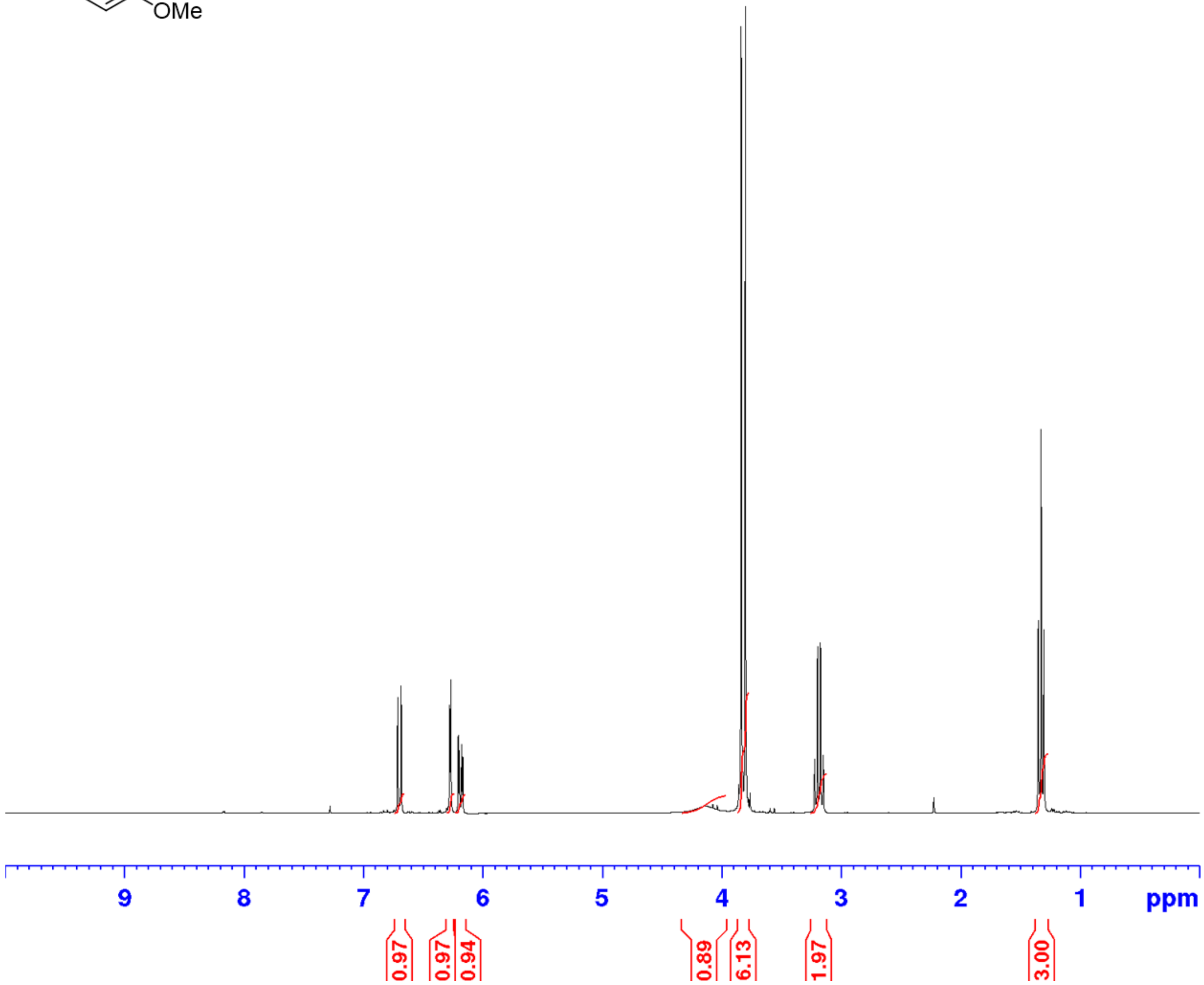
9 8 7 6 5 4 3 2 1 ppm
0.97 0.97 0.94 0.89 6.13 1.97 3.00

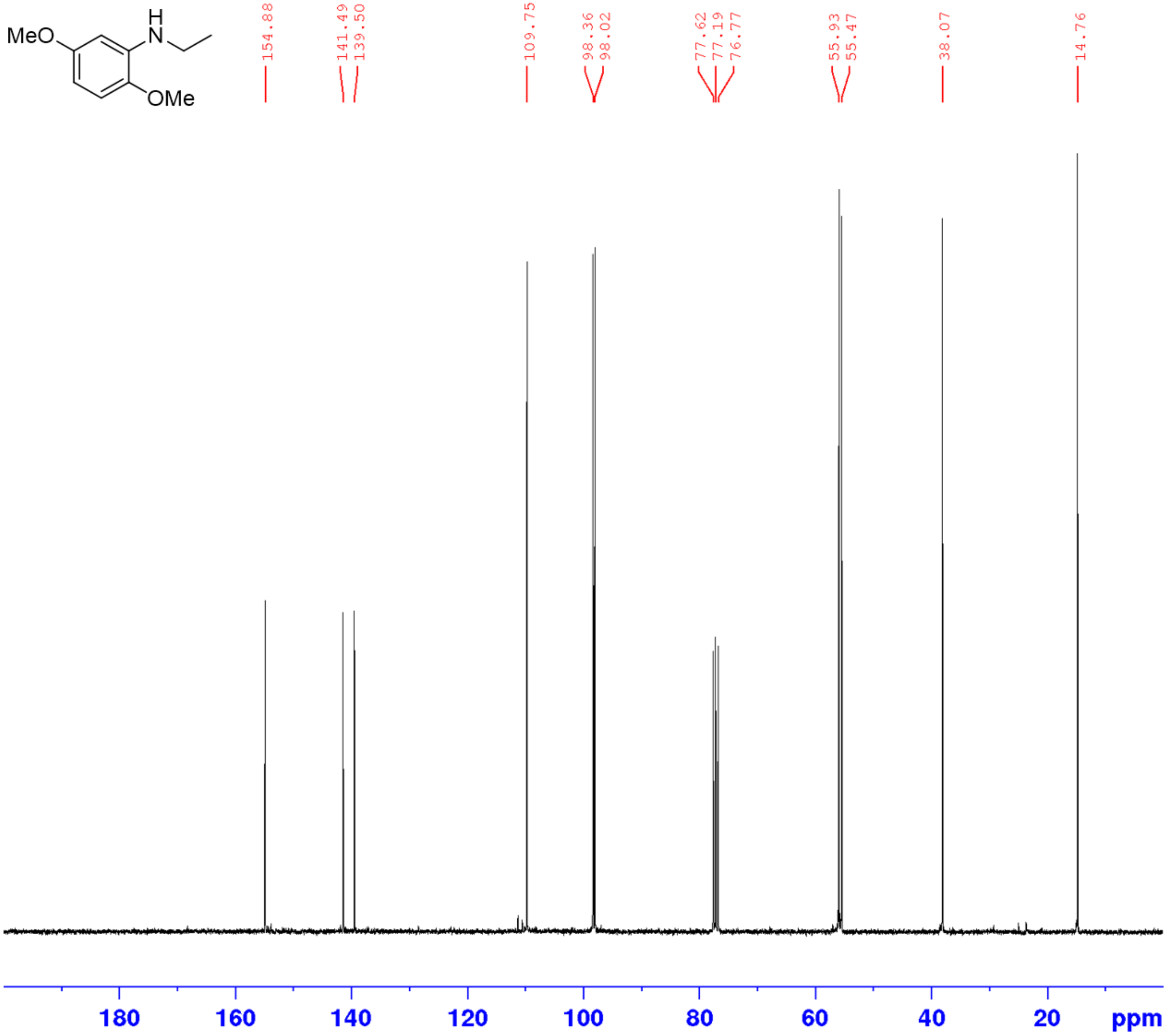

MeO
H
N
OMe
154.88
141.49
139.50
109.75
98.36
98.02
77.62
77.19
76.77
55.93
55.47
38.07
14.76
180
160
140
120
100
80
60
40
20
ppm

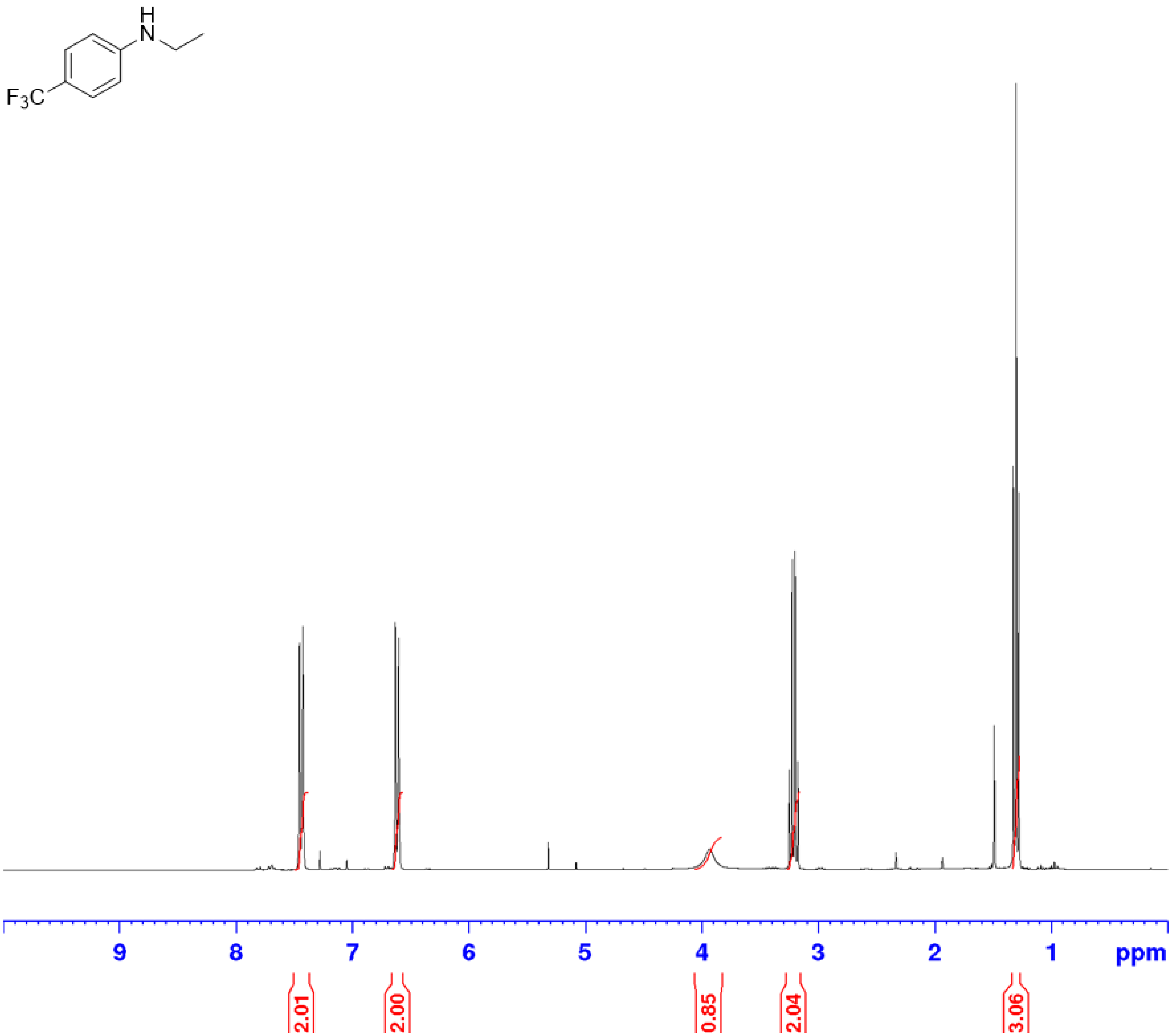

F3C
H
N
9
8
7
6
5
4
3
2
1
ppm
2.01
2.00
0.85
2.04
3.06

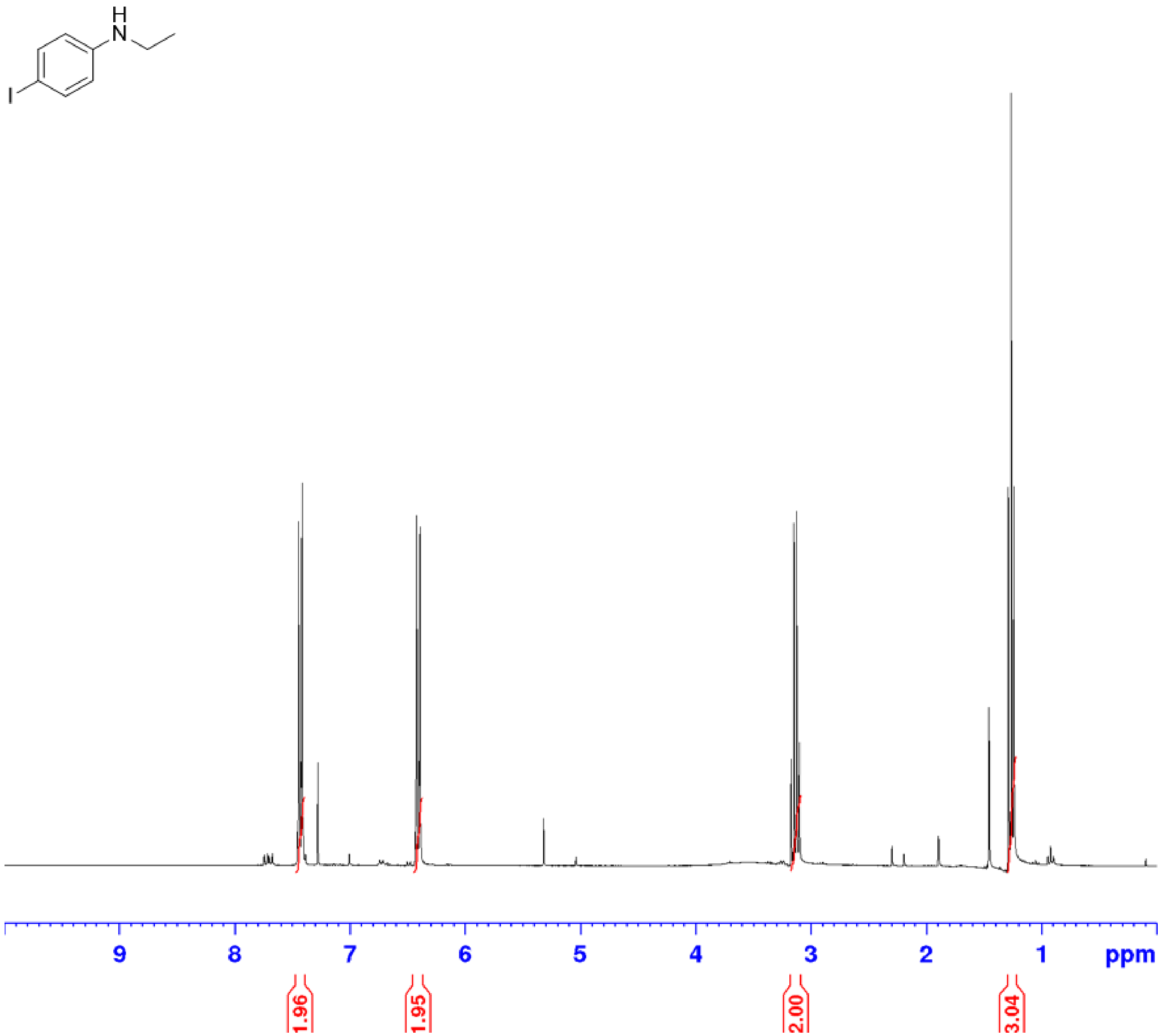
H
N
I
9
8
7
6
5
4
3
2
1
ppm
1.96
1.95
2.00
3.04

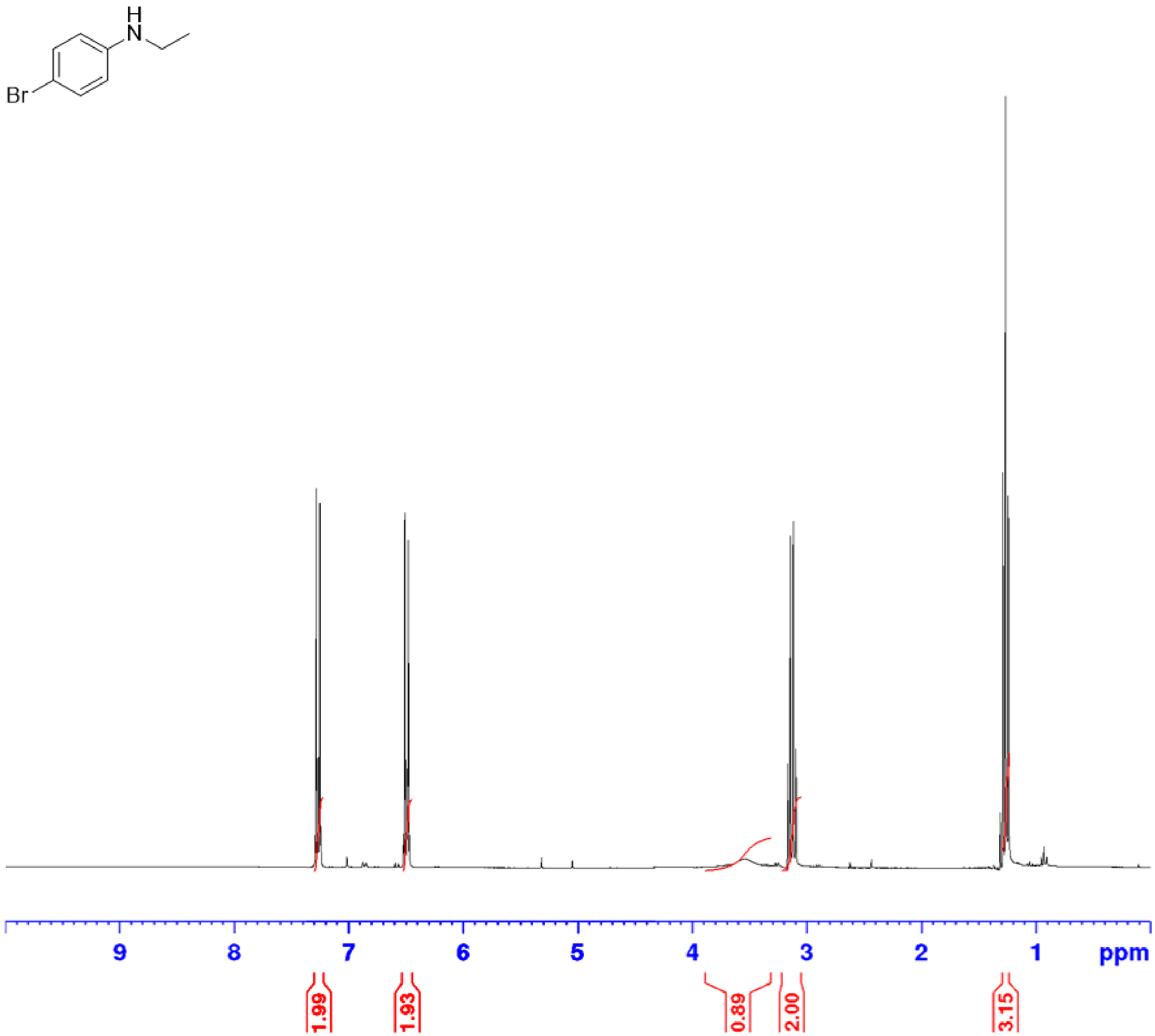

Br
H
N
9
8
7
6
5
4
3
2
1
ppm
1.99
1.93
0.89
2.00
3.15

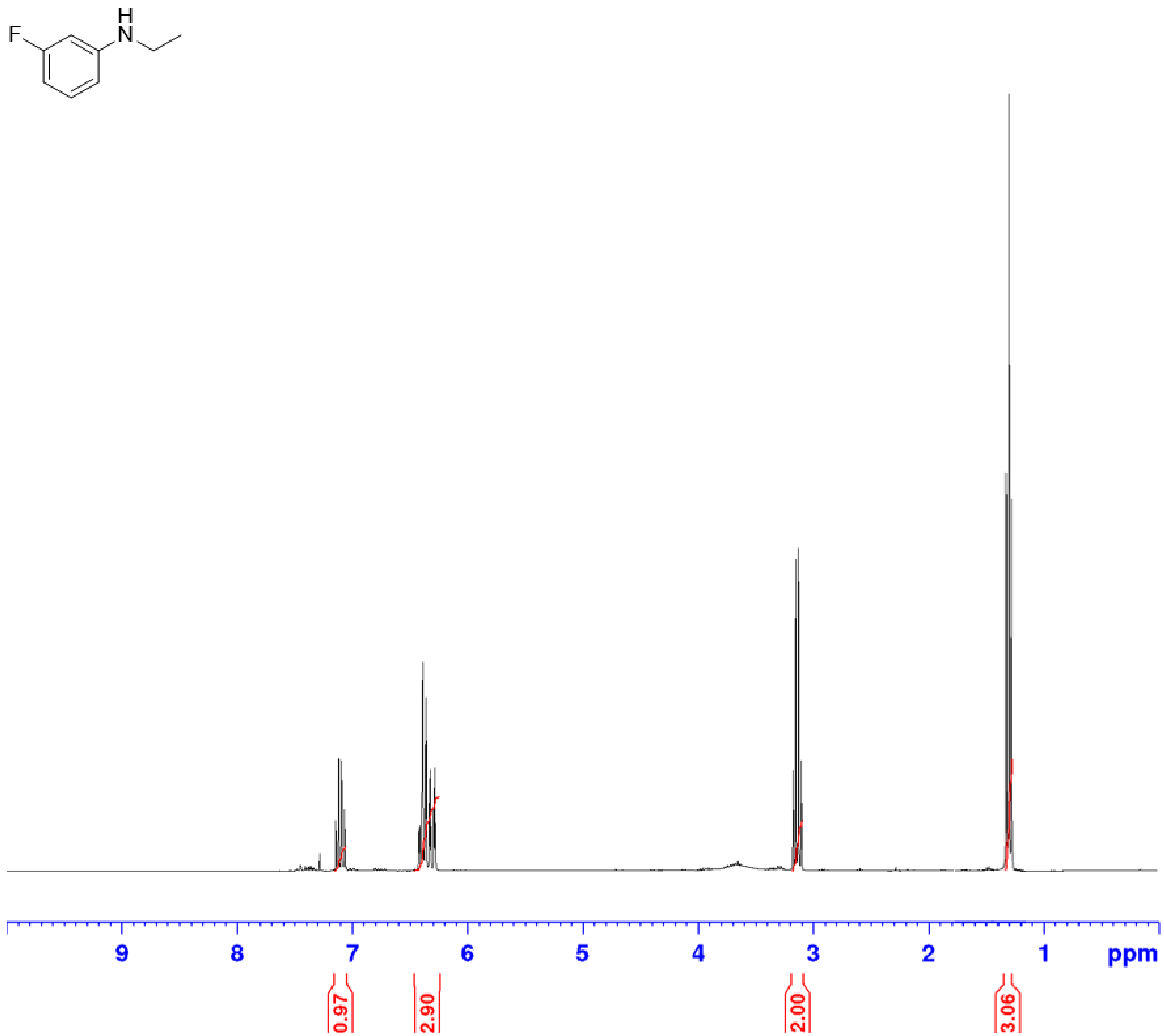

F
H
N
9
8
7
6
5
4
3
2
1
ppm
0.97
2.90
2.00
3.06

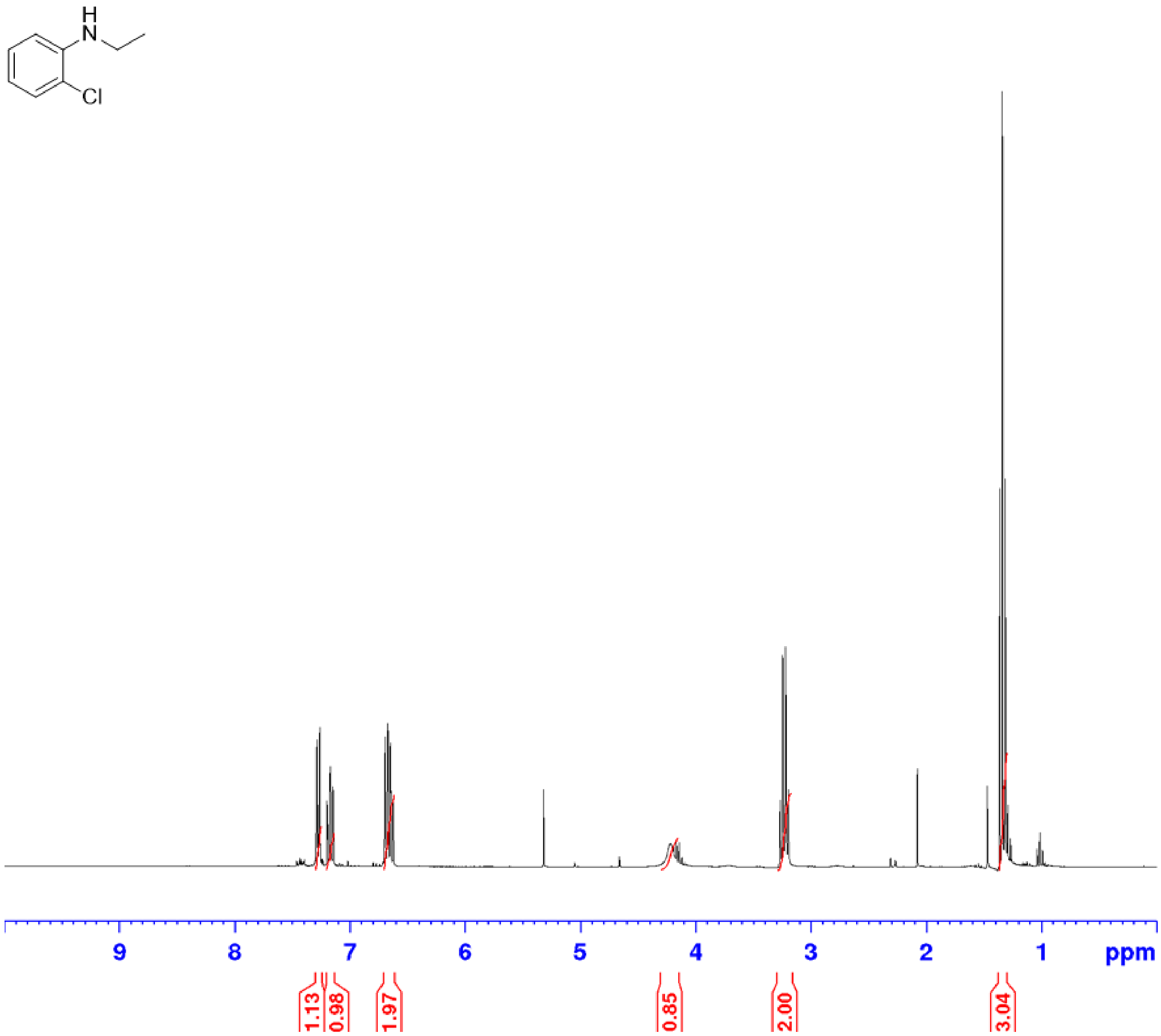

H
N
Cl
9
8
7
6
5
4
3
2
1
ppm
1.13
0.98
1.97
0.85
2.00
3.04

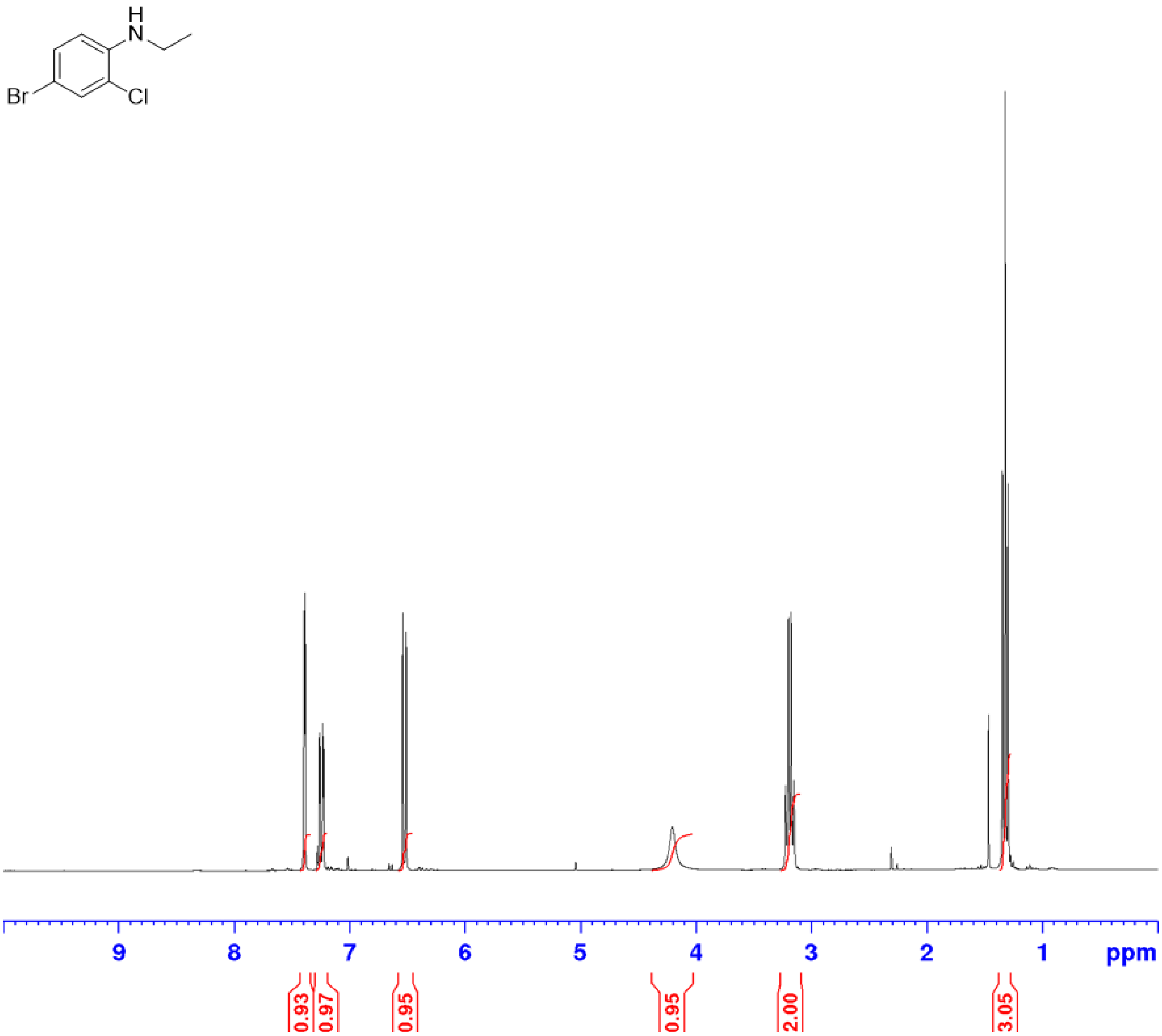

H
N
Br
Cl
9
8
7
6
5
4
3
2
1
ppm
0.93
0.97
0.95
0.95
2.00
3.05

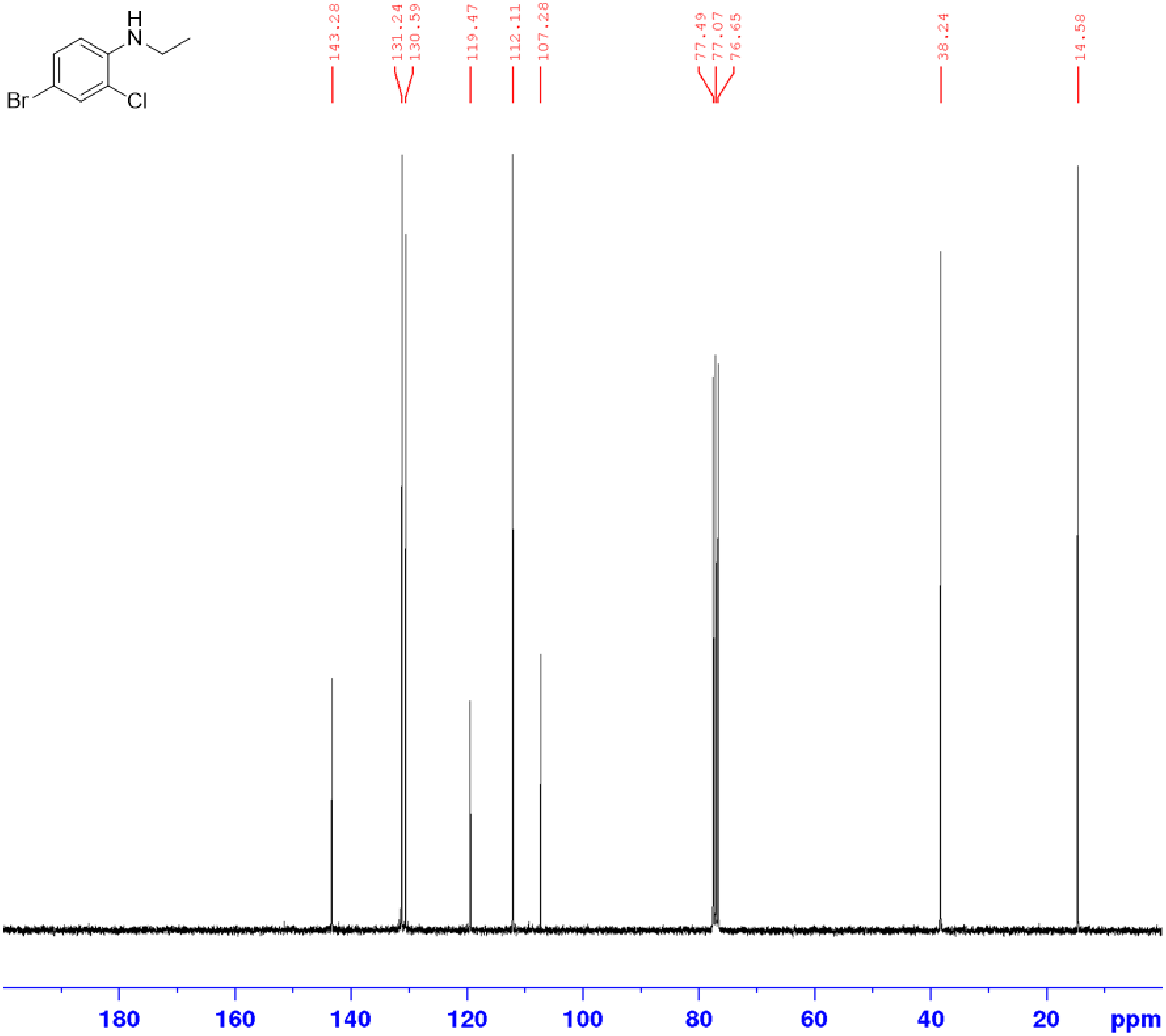
143.28
131.24
130.59
119.47
112.11
107.28
77.49
77.07
76.65
38.24
14.58
180
160
140
120
100
80
60
40
20
ppm

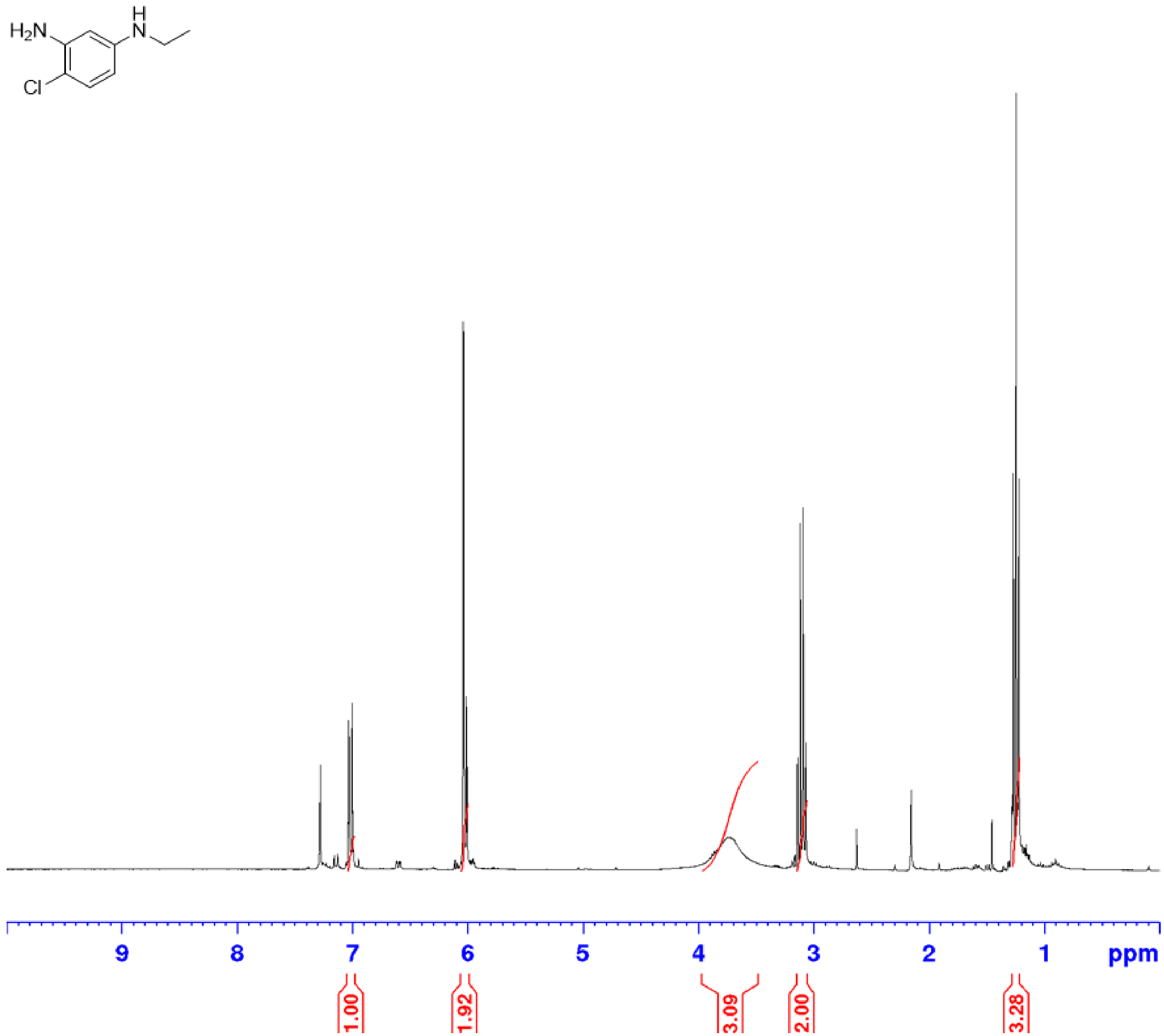

H
N
H2N
Cl
9
8
7
6
5
4
3
2
1
ppm
1.00
1.92
3.09
2.00
3.28

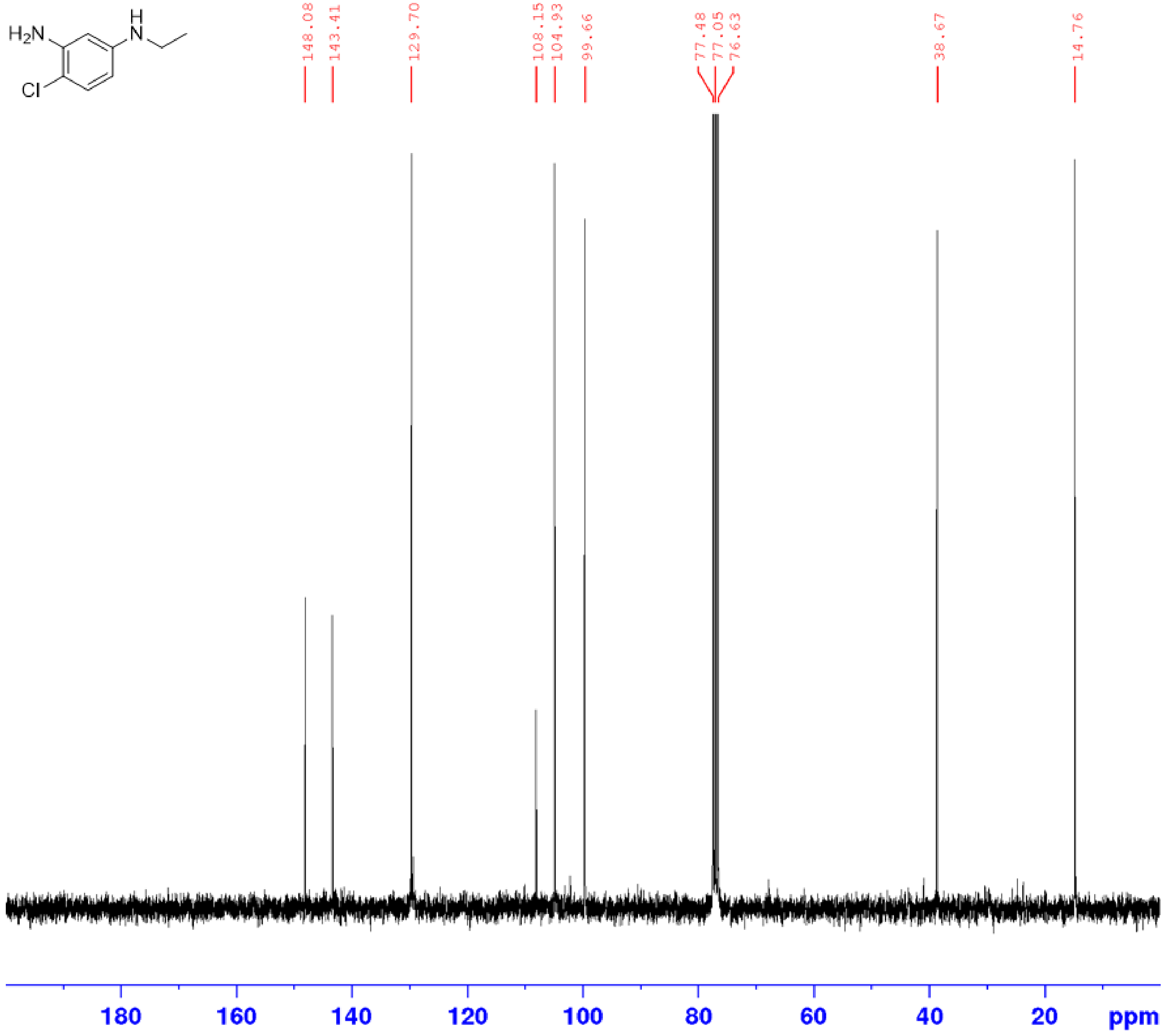

H2N
H
N
Cl
148.08
143.41
129.70
108.15
104.93
99.66
77.48
77.05
76.63
38.67
14.76
180
160
140
120
100
80
60
40
20
ppm

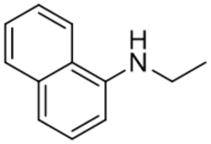
H
N

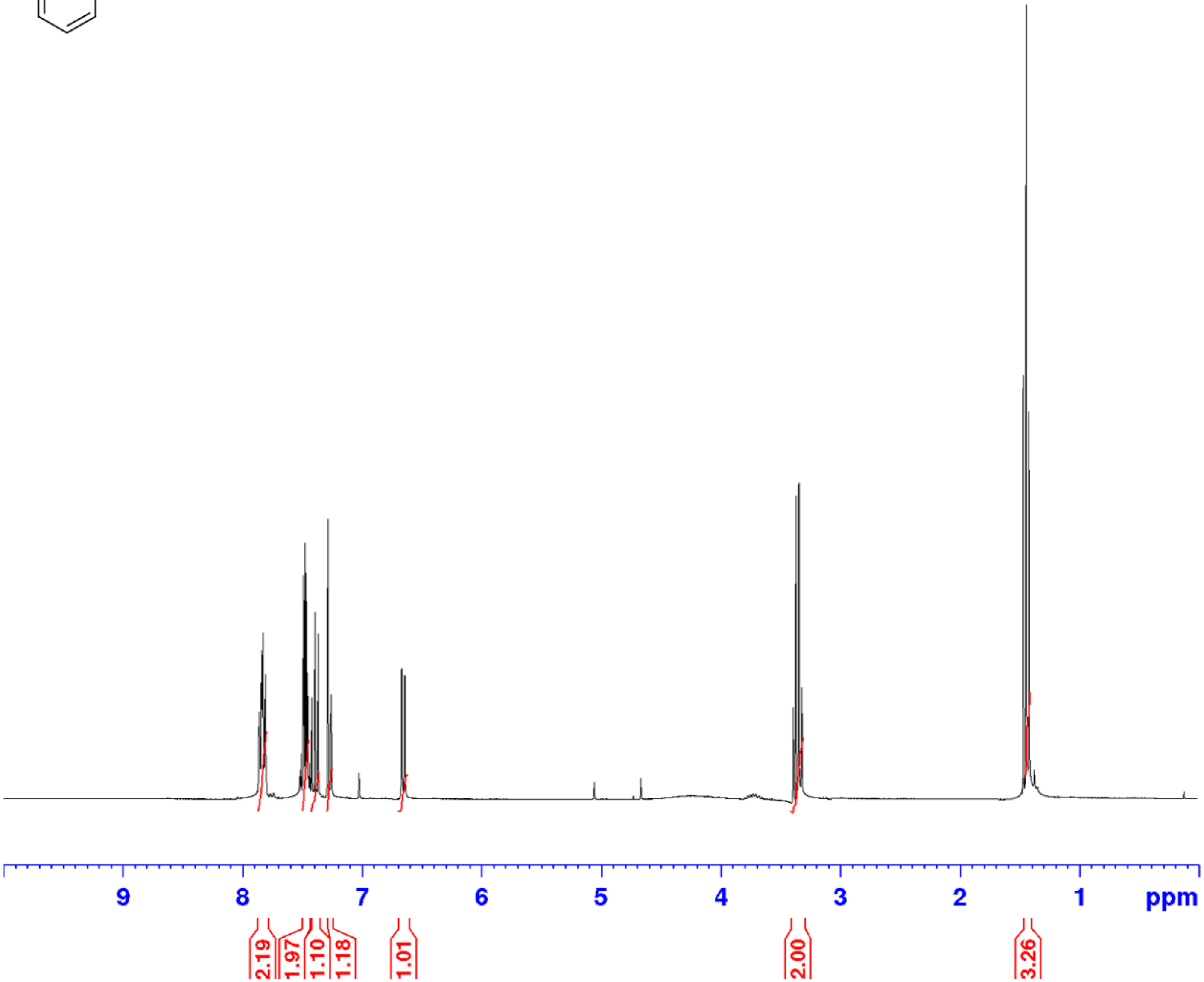
9
8
7
6
5
4
3
2
1
ppm
2.19
1.97
1.10
1.18
1.01
2.00
3.26

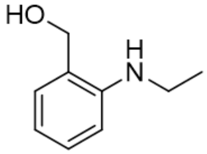
HO
H
N

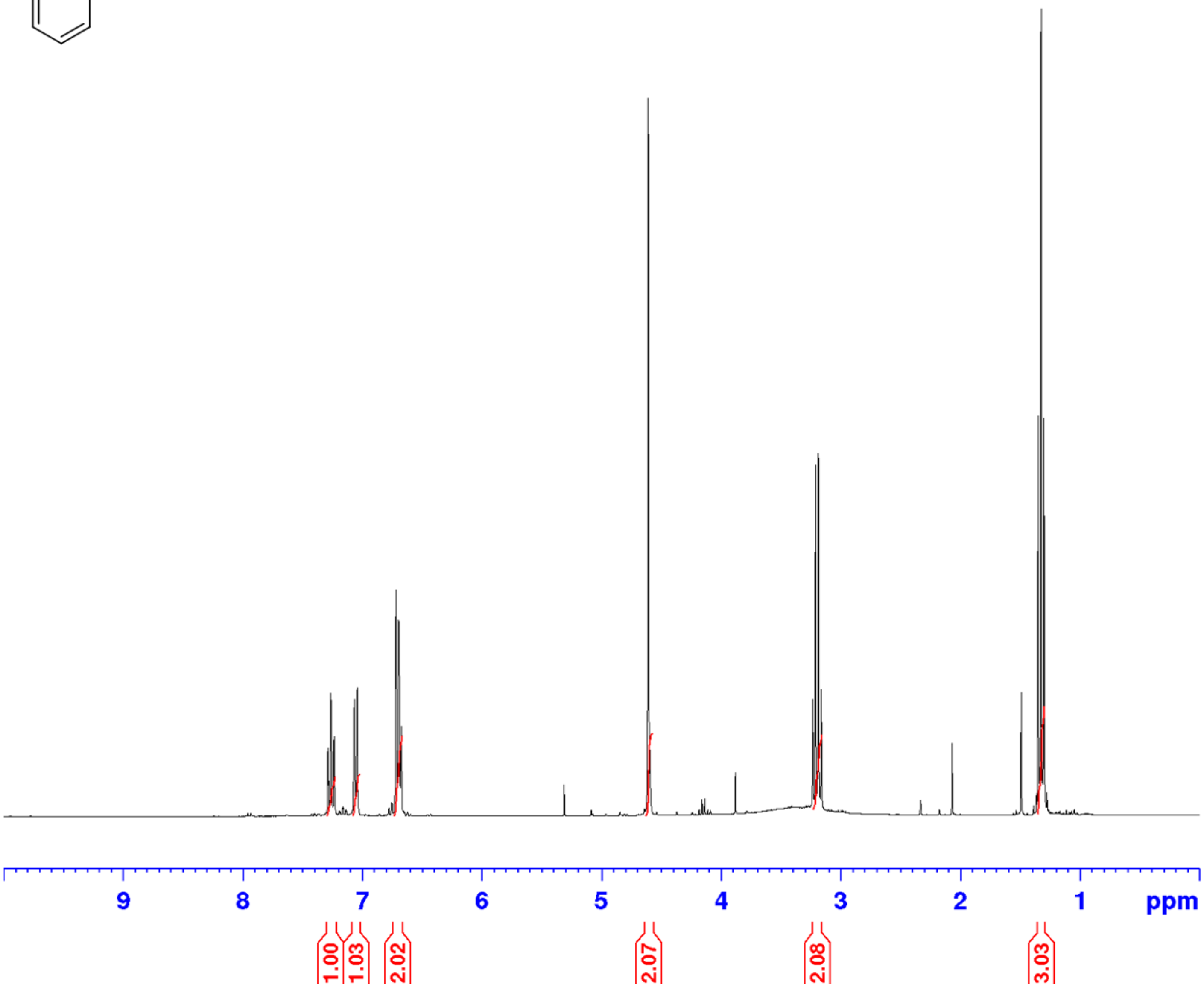
9
8
7
6
5
4
3
2
1
ppm
1.00
1.03
2.02
2.07
2.08
3.03

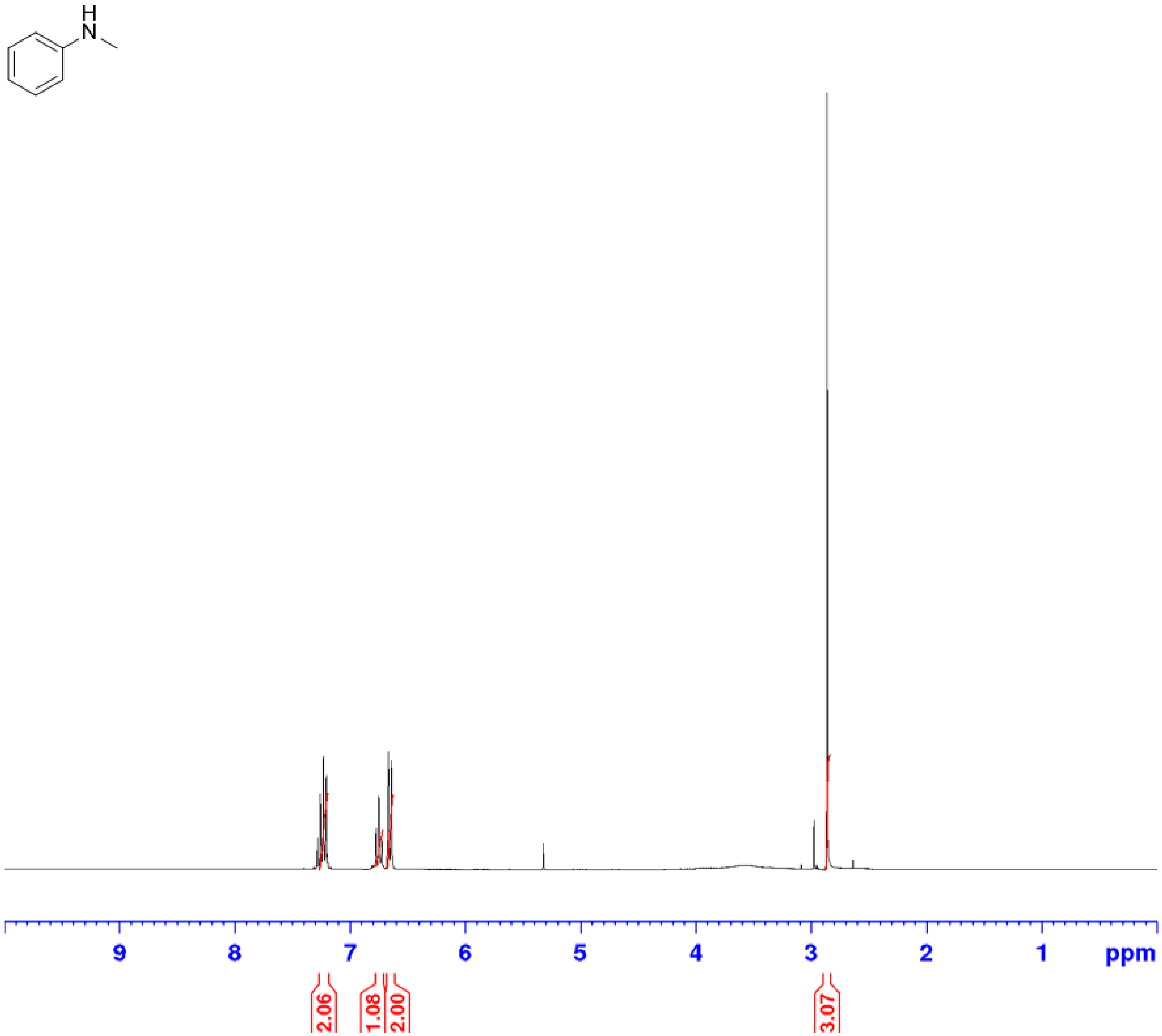

H
N
9
8
7
6
5
4
3
2
1
ppm
2.06
1.08
2.00
3.07

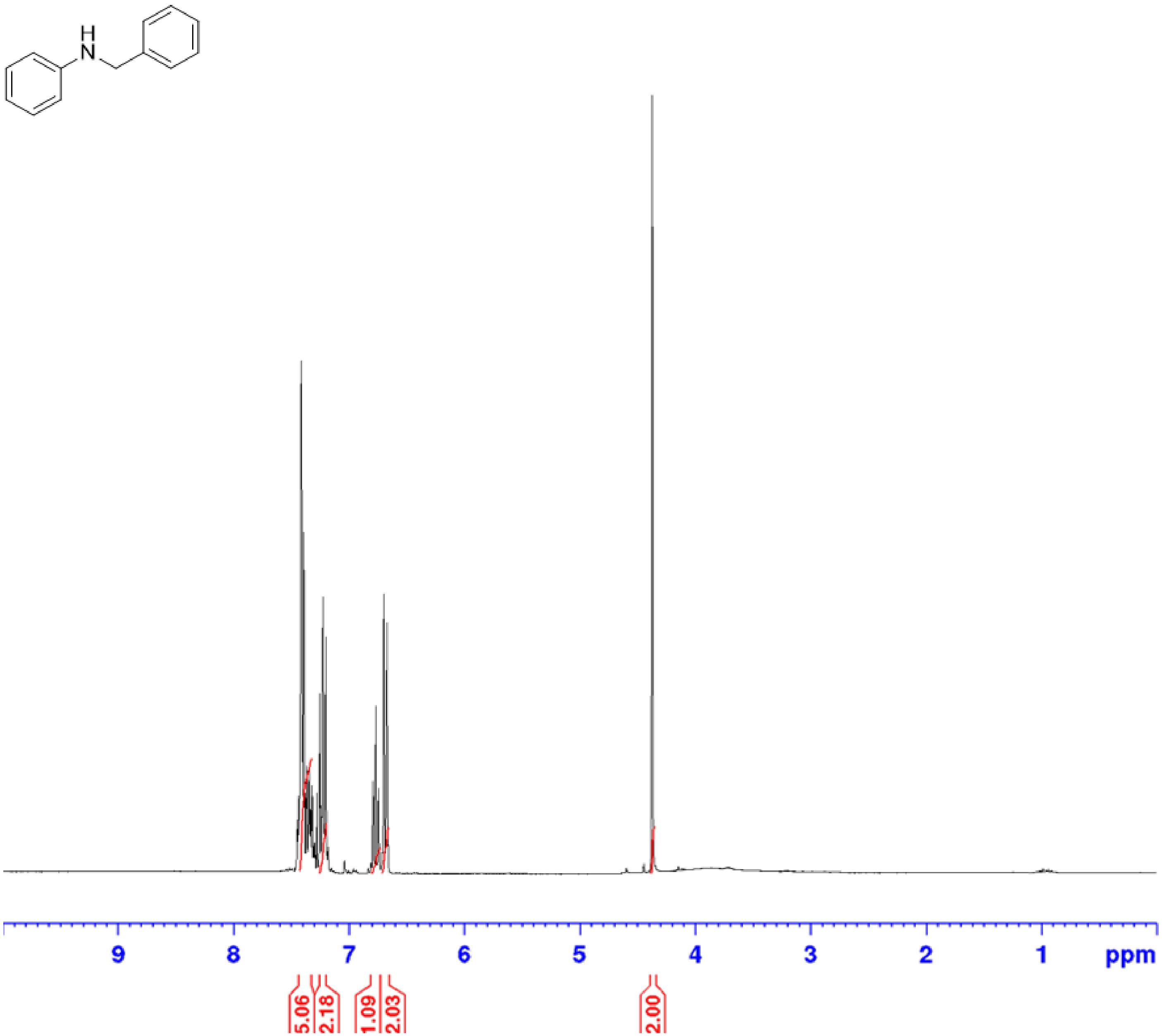
H
N
9
8
7
6
5
4
3
2
1
ppm
5.06
2.18
1.09
2.03
2.00

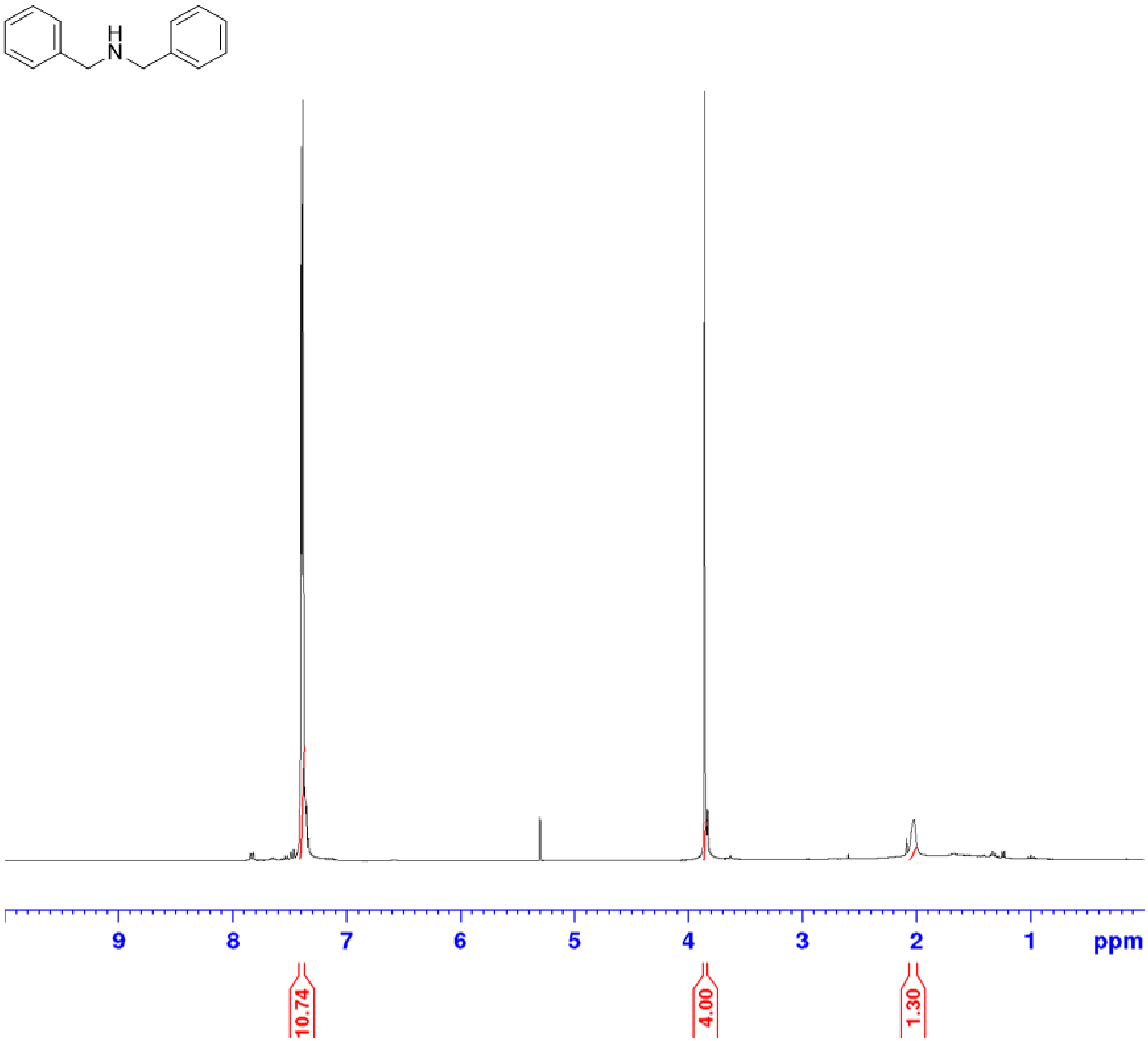
H
N
9
8
7
6
5
4
3
2
1
ppm
10.74
4.00
1.30

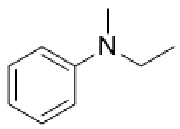

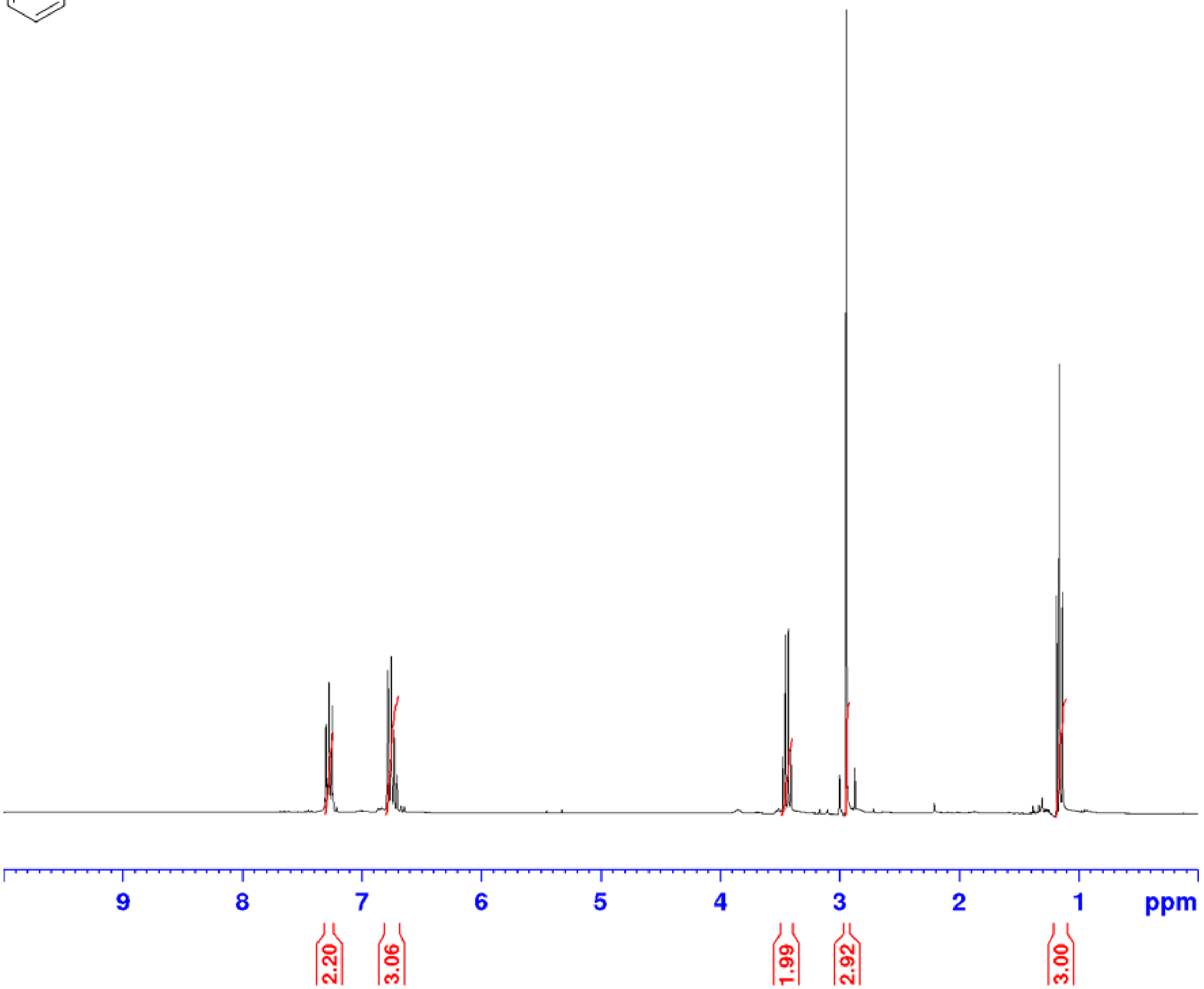

9
8
7
6
5
4
3
2
1
ppm
2.20
3.06
1.99
2.92
3.00

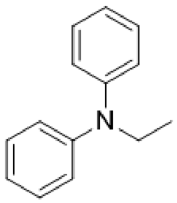

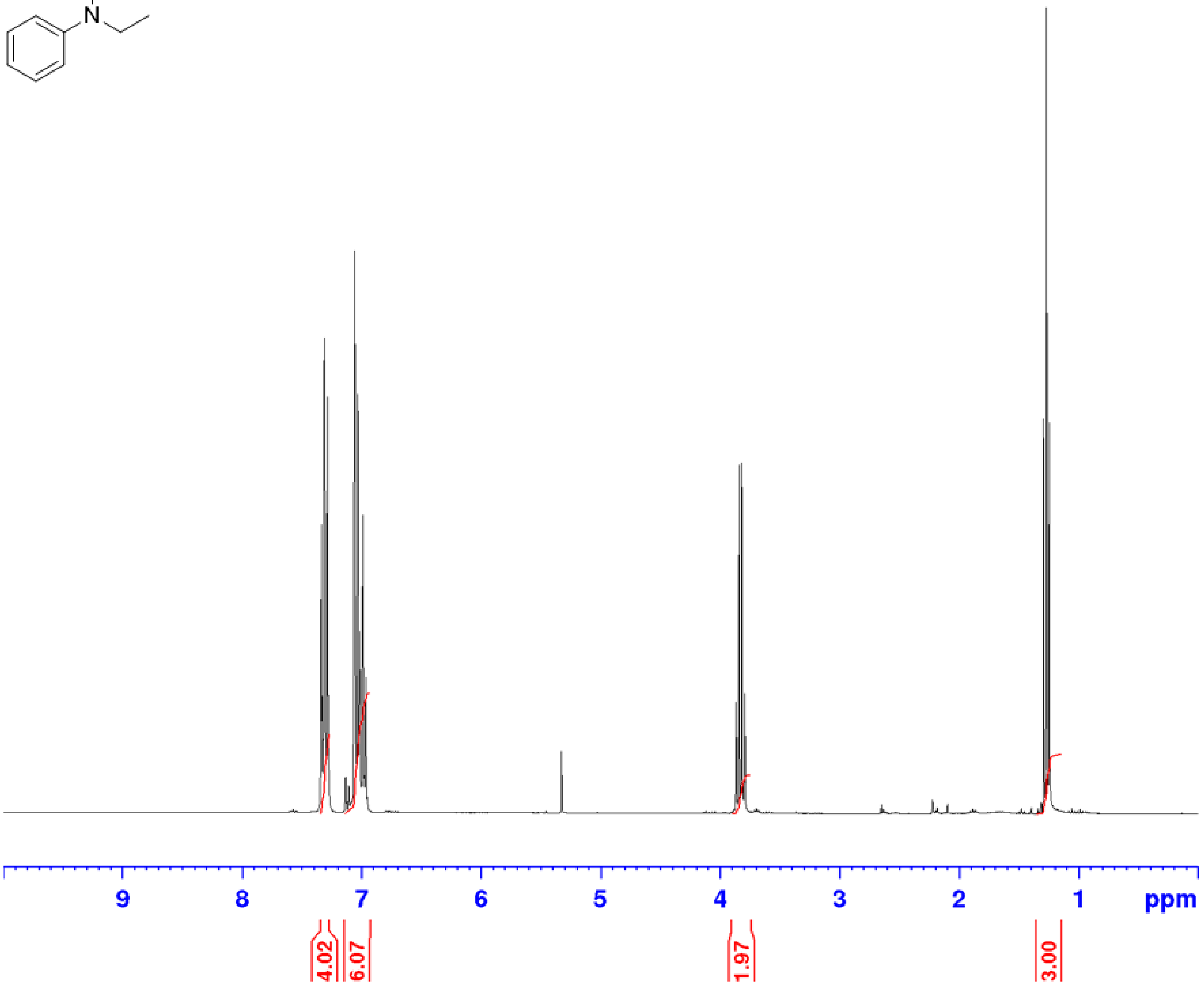
9
8
7
6
5
4
3
2
1
ppm
4.02
6.07
1.97
3.00

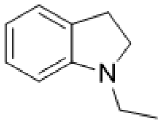

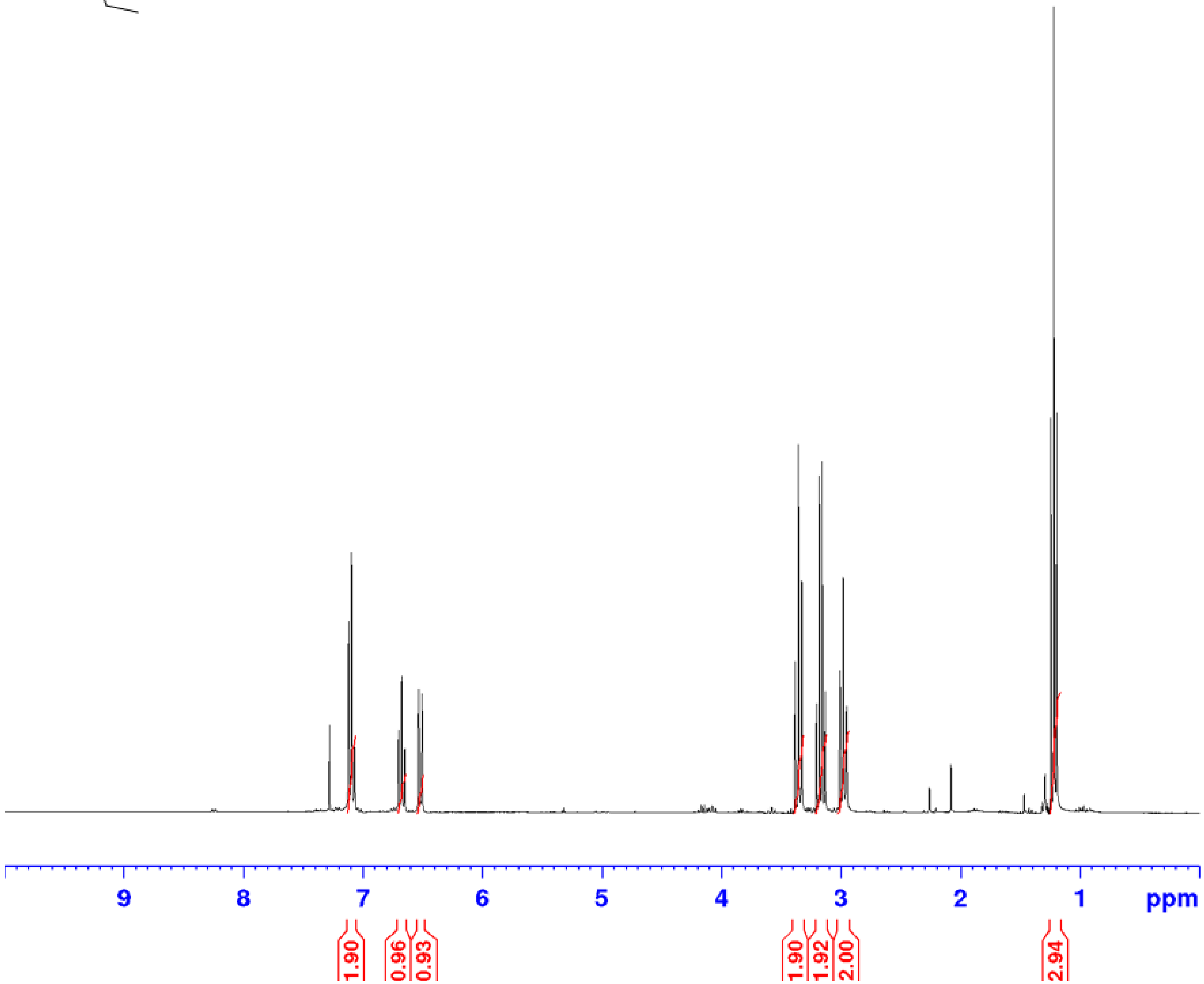

9
8
7
6
5
4
3
2
1
ppm
1.90
0.96
0.93
1.90
1.92
2.00
2.94

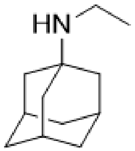
HN

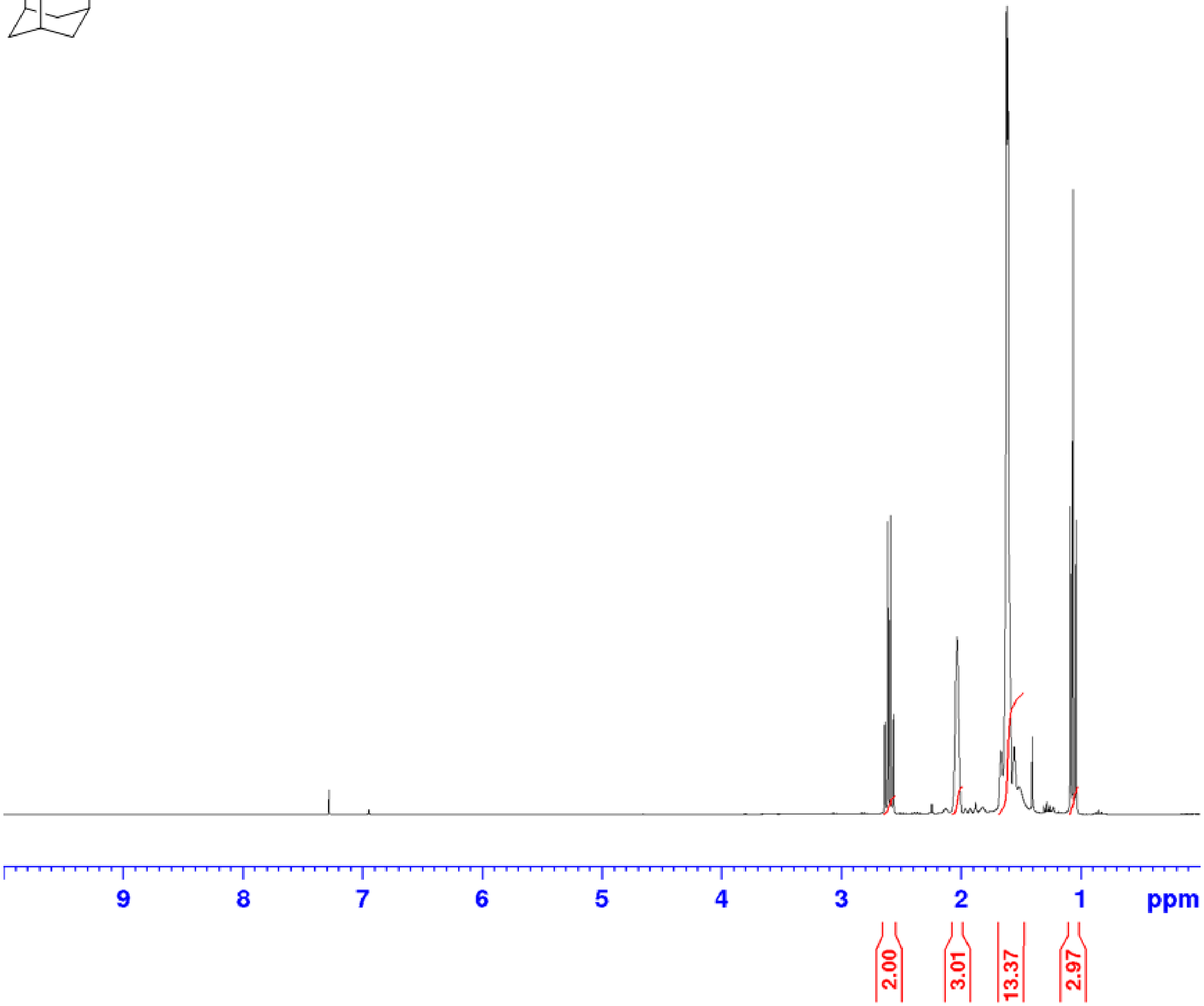
9
8
7
6
5
4
3
2
1
ppm
2.00
3.01
13.37
2.97

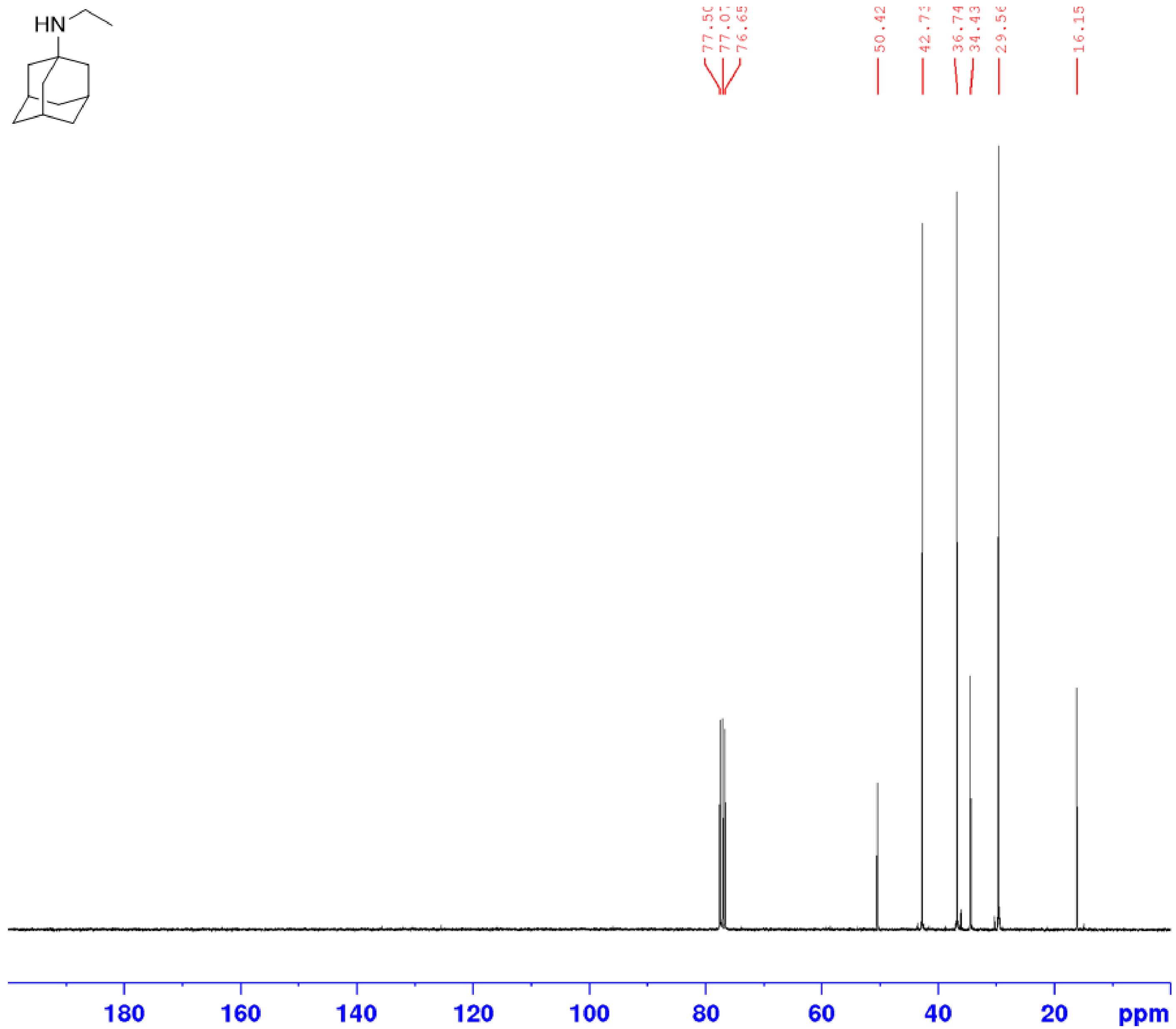
HN
50.42
36.74
34.43
16.15
180
160
140
120
100
80
60
40
20
ppm

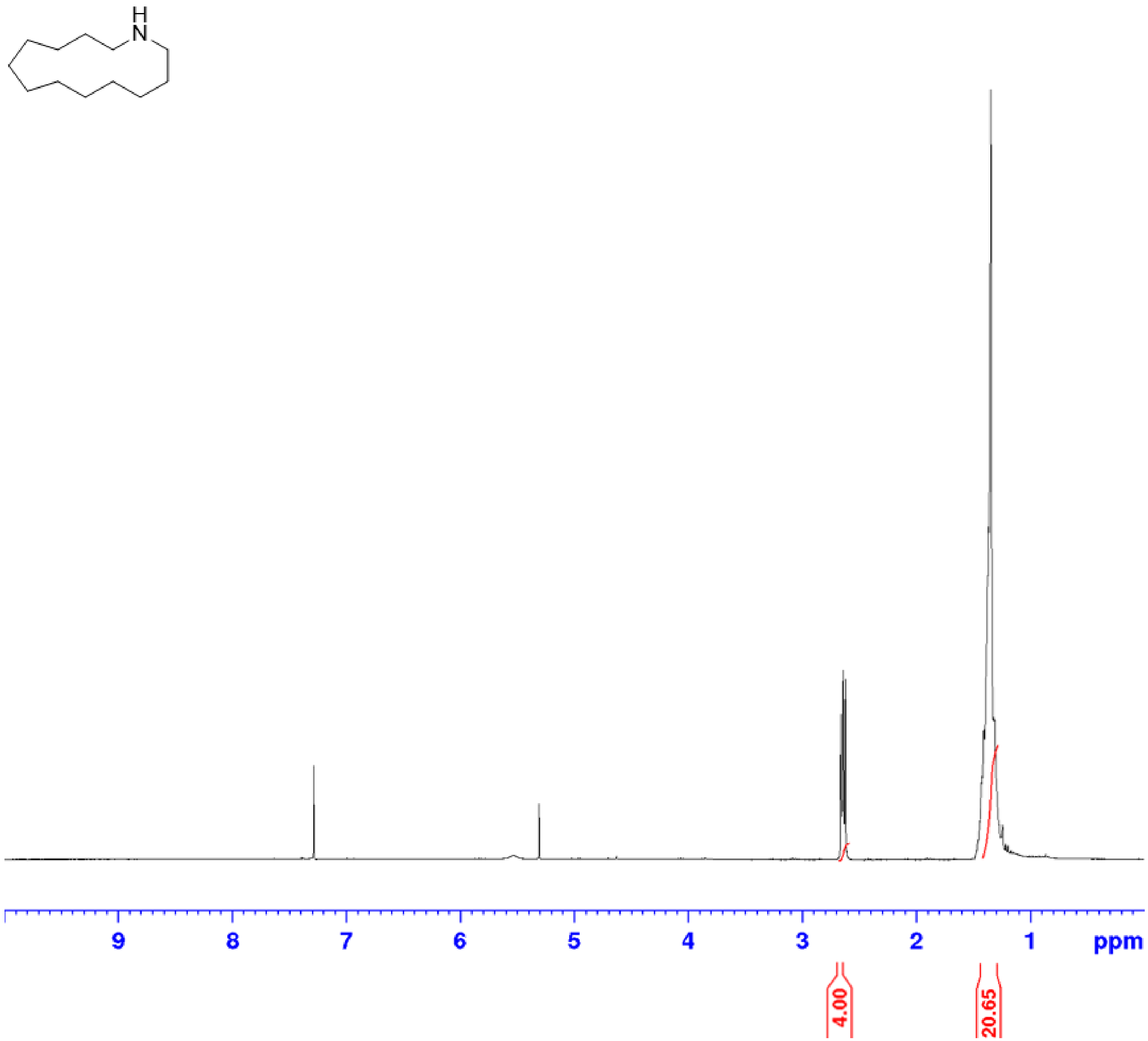

H
N
9
8
7
6
5
4
3
2
1
ppm
4.00
20.65

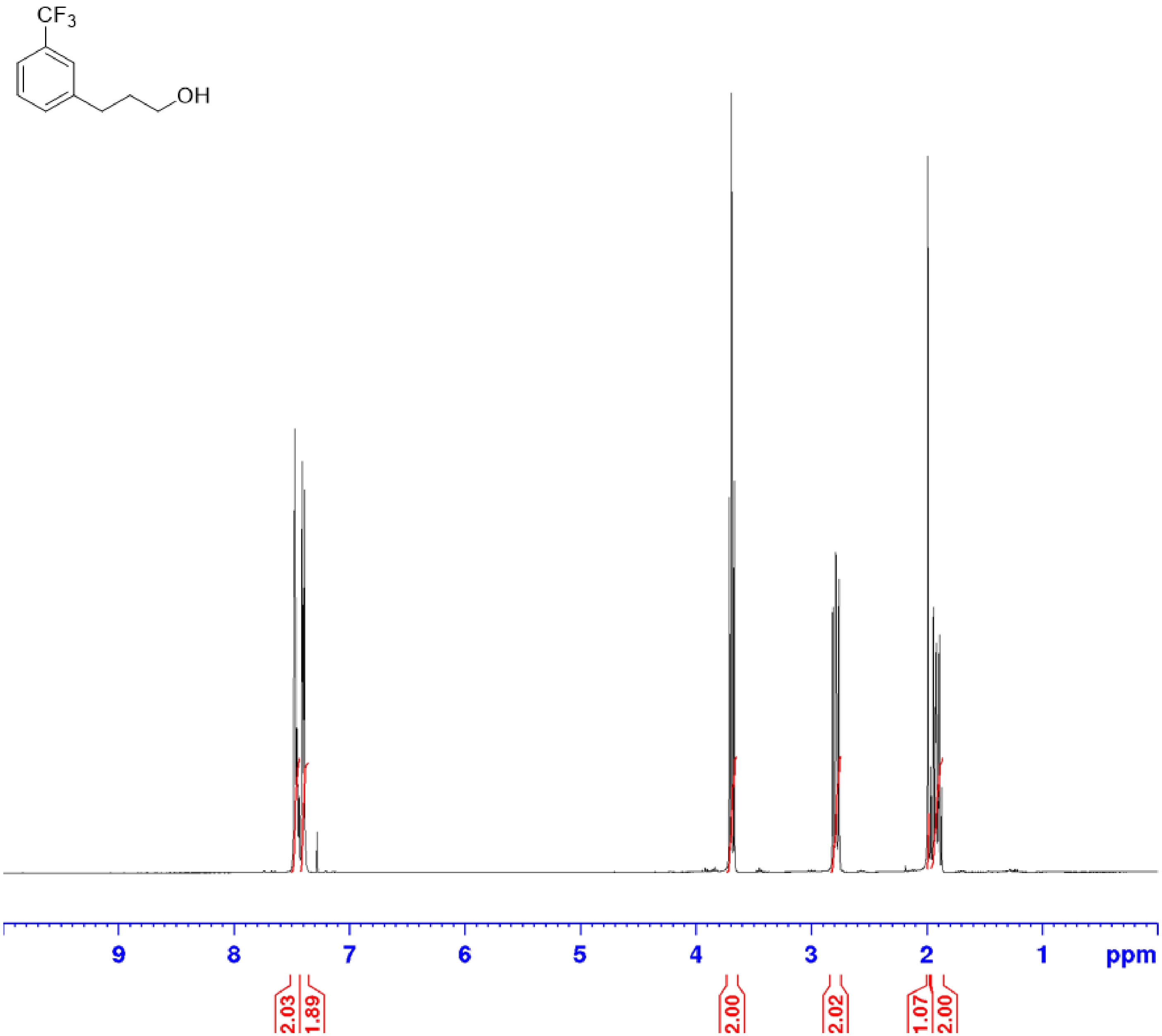

CF3
OH
9
8
7
6
5
4
3
2
1
ppm
2.03
1.89
2.00
2.02
1.07
2.00

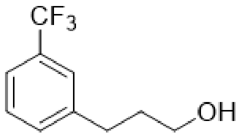
CF3
OH

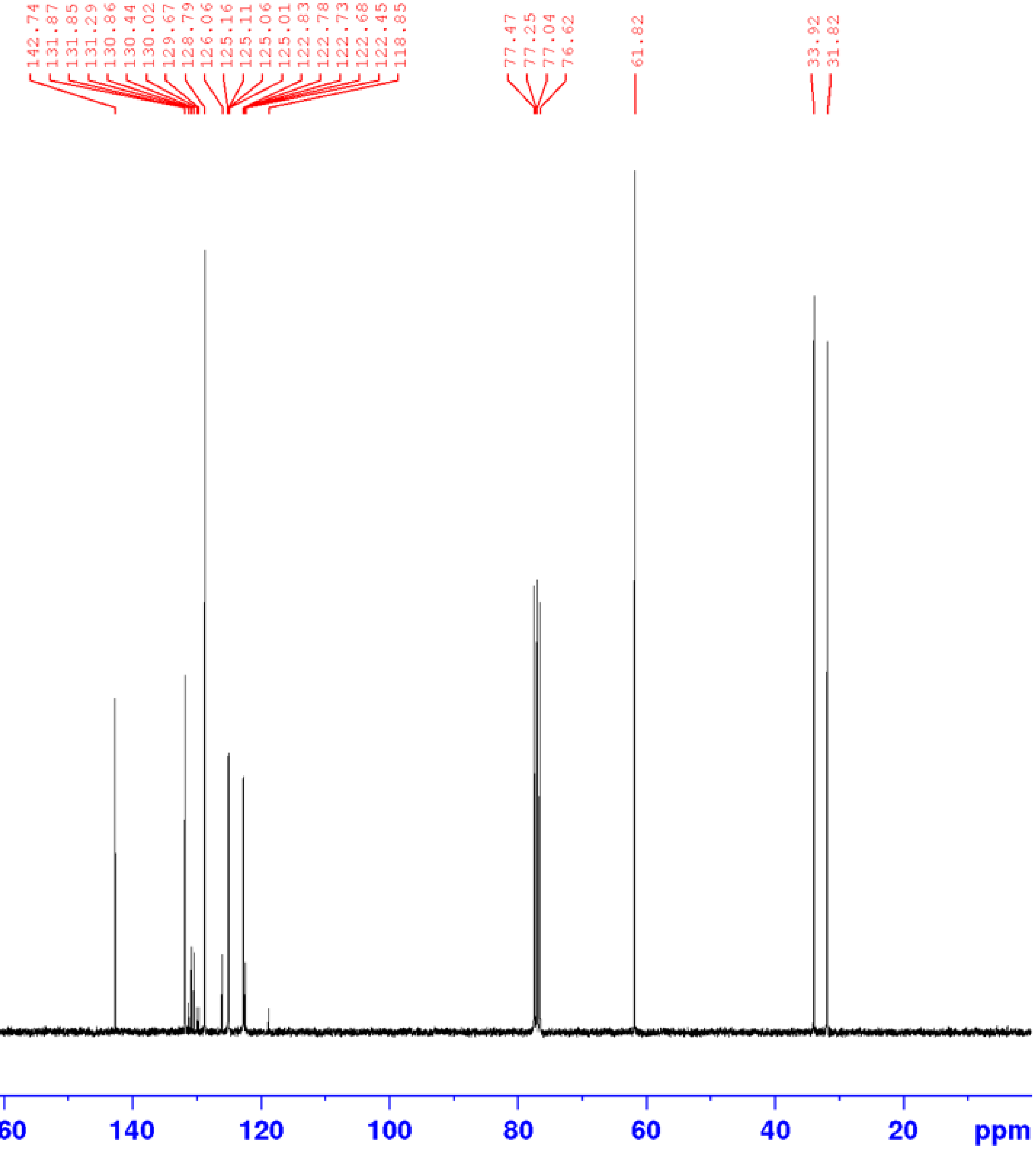
142.74
131.87
131.85
131.29
130.86
130.44
130.02
129.67
128.79
126.06
125.16
125.11
125.06
125.01
122.83
122.78
122.73
122.68
122.45
118.85
77.47
77.25
77.04
76.62
61.82
33.92
31.82
180
160
140
120
100
80
60
40
20
ppm

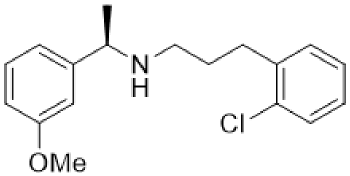

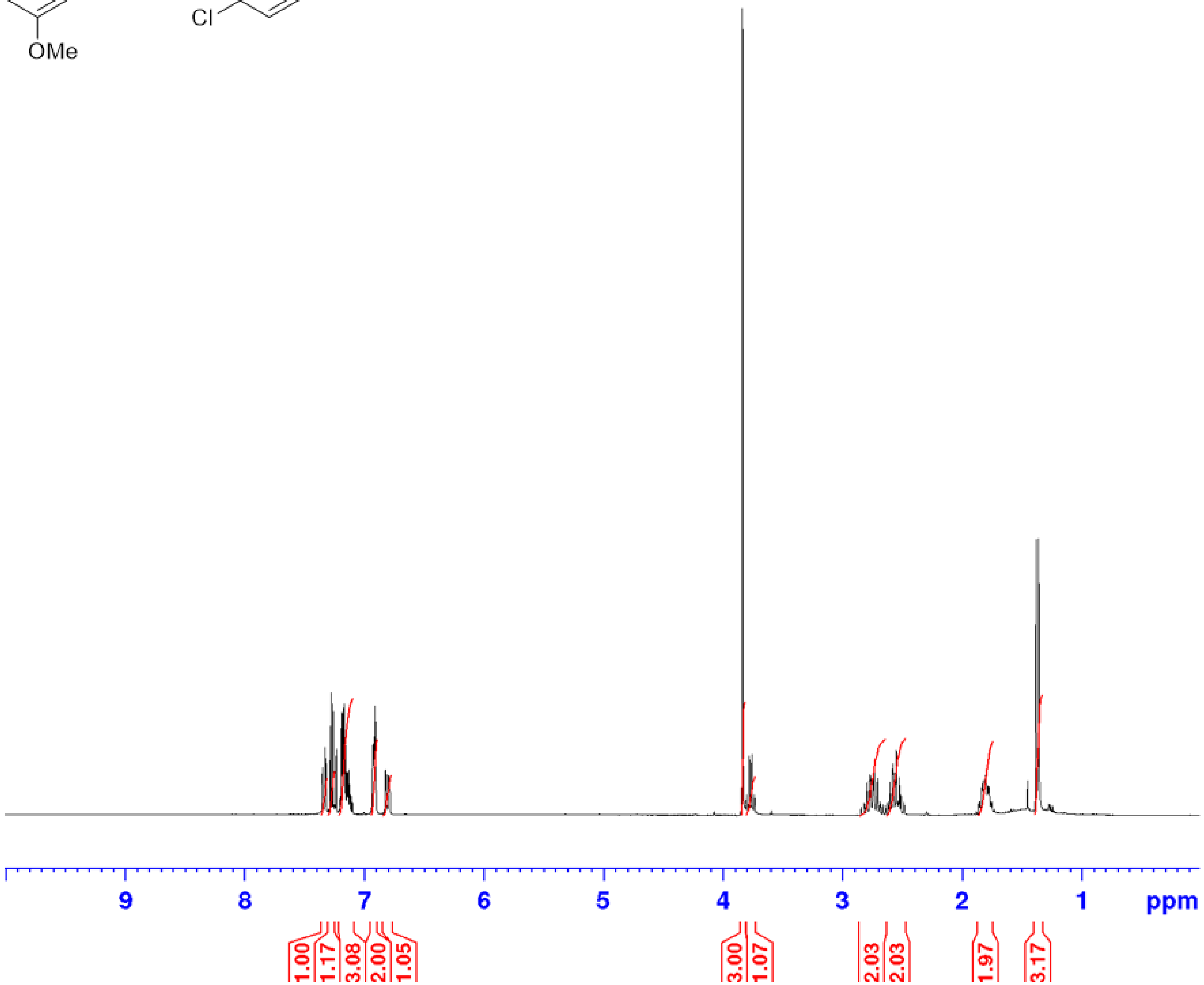
9
8
7
6
5
4
3
2
1
ppm
1.00
1.17
3.08
2.00
1.05
3.00
1.07
2.03
2.03
1.97
3.17

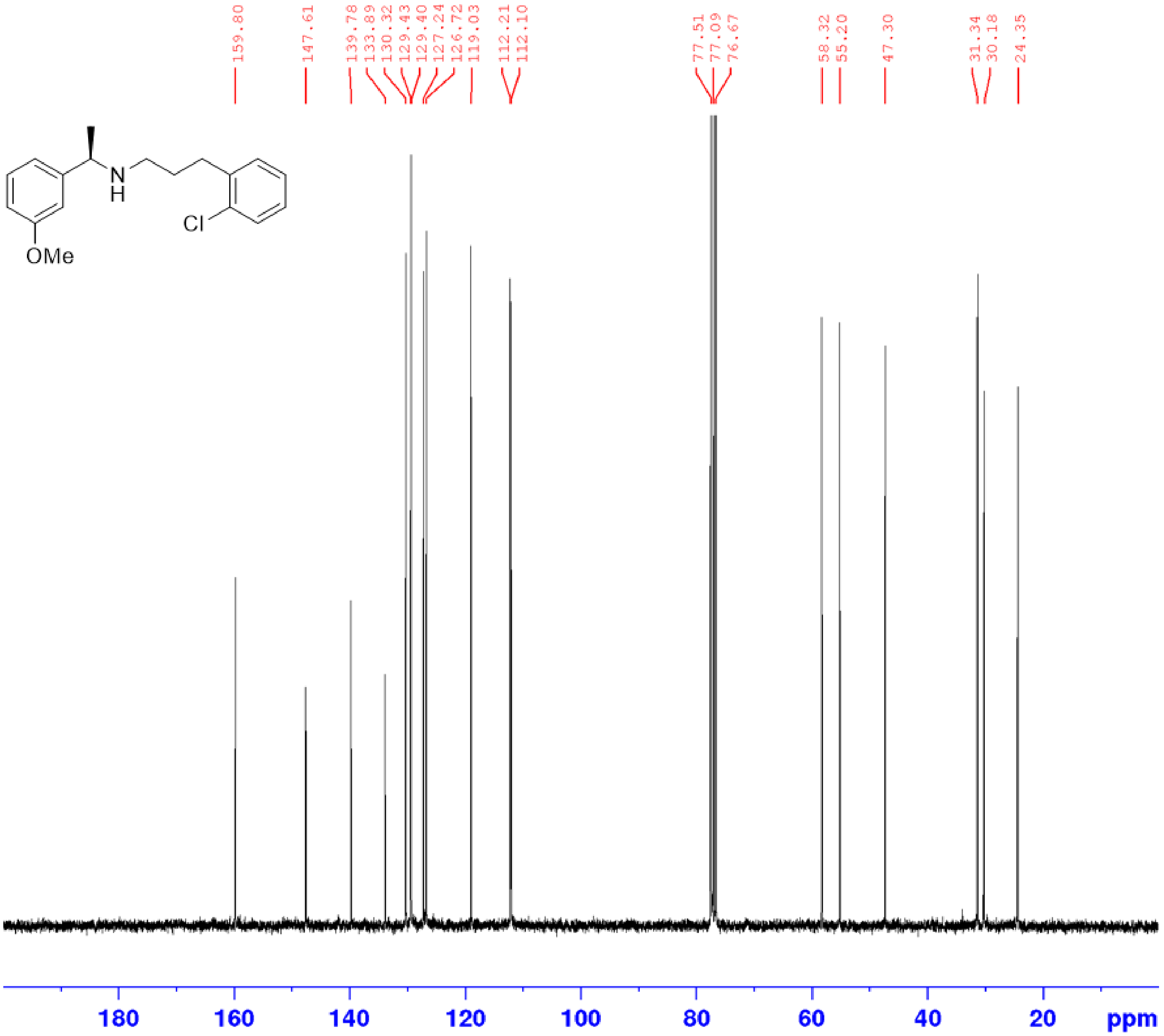
159.80
147.61
139.78
133.89
130.32
129.43
129.40
127.24
126.72
119.03
112.21
112.10
77.51
77.09
76.67
58.32
55.20
47.30
31.34
30.18
24.35
N
H
Cl
OMe
180
160
140
120
100
80
60
40
20
ppm

4. References

[1] N. S. Shaikh, K. Junge, M. Beller, *Org. Lett.* **2007**, *9*, 5429-5432.

[2] D. Y. Ong, Z. Yen, A. Yoshii, J. Revillo Imbernon, R. Takita, S. Chiba, *Angew. Chem. Int. Ed.* **2019**, *58*, 4992-4997.

[3] A. H. Aboo, E. L. Bennett, M. Deeprose, C. M. Robertson, J. A. Iggo, J. Xiao, *Chem. Commun.* **2018**, *54*, 11805-11808.

[4] Y. Corre, V. Rysak, X. Trivelli, F. Agbossou-Niedercorn, C. Michon, *Eur. J. Org. Chem.* **2017**, 4820-4826.

[5] S. Sharma, D. Bhattacherjee, P. Das, *Adv. Synth. Catal.* **2018**, *360*, 2131-2137.

[6] D. A. Everson, R. Shrestha, D. J. Weix, *J. Am. Chem. Soc.* **2010**, *132*, 920-921.

[7] K.-S. Song, S. H. Lee, M. J. Kim, H. J. Seo, J. Lee, S.-H. Lee, M. E. Jung, E.-J. Son, M. Lee, J. Kim, J. Lee, *ACS Med. Chem. Lett.* **2011**, *2*, 182-187.

[8] H. Yang, Y. Li, M. Jiang, J. Wang, H. Fu, *Chem. Eur. J.* **2011**, *17*, 5652-5660.

[9] S. R. Roy, S. C. Sau, S. K. Mandal, *J. Org. Chem.* **2014**, *79*, 9150-9160.

[10] M. K. Barman, K. Das, B. Maji, *J. Org. Chem.* **2019**, *84*, 1570-1579.

[11] J. J. Eisch, S. Dutta, J. N. Gitua, *Organometallics* **2005**, *24*, 6291-6294.

[12] S. Jana, V. A. Sarpe, S. S. Kulkarni, *Org. Lett.* **2018**, *20*, 6938-6942.

[13] Y. Pan, Z. Luo, X. Xu, H. Zhao, J. Han, L. Xu, Q. Fan, J. Xiao, *Adv. Synth. Catal.* **2019**, *361*, 3800-3806.

[14] A. B. Enyong, B. Moasser, *J. Org. Chem.* **2014**, *79*, 7553-7563.

[15] A. Lator, S. Gaillard, A. Poater, J.-L. Renaud, *Org. Lett.* **2018**, *20*, 5985-5990.

[16] Y. Pan, Z. Luo, J. Han, X. Xu, C. Chen, H. Zhao, L. Xu, Q. Fan, J. Xiao, *Adv. Synth. Catal.* **2019**, *361*, 2301-2308.

[17] G. Bar-Haim, M. Kol, *Org. Lett.* **2004**, *6*, 3549-3551.

[18] Y. Wei, Q. Xuan, Y. Zhou, Q. Song, *Org. Chem. Front.* **2018**, *5*, 3510-3514.

[19] S. S. Bhojgude, T. Kaicharla, A. T. Biju, *Org. Lett.* **2013**, *15*, 5452-5455.

[20] M. H. Weston, K. Nakajima, T. G. Back, *J. Org. Chem.* **2008**, *73*, 4630-4637.

[21] S. Kramer, *Org. Lett.* **2019**, *21*, 65-69.

[22] X. Xi, Y. Li, G. Wang, G. Xu, L. Shang, Y. Zhang, L. Xia, *Org. Biomol. Chem.* **2019**, *17*, 7651-7654.

[23] A. Chelouan, R. Recio, L. G. Borrego, E. Álvarez, N. Khiar, I. Fernández, *Org. Lett.* **2016**, *18*, 3258-3261.
